# Graminoids Increase Greenhouse Gas Emissions From Thawed Permafrost at the End of the Growing Season

**DOI:** 10.1111/gcb.70783

**Published:** 2026-03-09

**Authors:** Marie Mollenkopf, Katja Lenge, Sören Drabesch, Sylvain Monteux, Sigrid van Grinsven, Prachi Joshi, Ellen Dorrepaal, Birgit Wild, Andreas Kappler, E. Marie Muehe

**Affiliations:** ^1^ Geomicrobiology, Department of Geosciences University of Tübingen Tübingen Germany; ^2^ Plant Biogeochemistry Helmholtz Centre for Environmental Research‐UFZ Leipzig Germany; ^3^ Plant Biogeochemistry, Department of Geosciences University of Tübingen Tübingen Germany; ^4^ Department of Environmental Science Stockholm University Stockholm Sweden; ^5^ Tromsø Museum UiT the Arctic University of Norway Tromsø Norway; ^6^ Forest Soils and Biogeochemistry Swiss Federal Institute for Forest, Snow and Landscape Research Birmendorf Switzerland; ^7^ Department of Ecology and Environmental Science, Climate Impacts Research Centre University of Umeå Abisko Sweden; ^8^ Bolin Centre for Climate Research Stockholm University Stockholm Sweden; ^9^ Cluster of Excellence: EXC 2124: Controlling Microbes to Fight Infection Tübingen Germany

**Keywords:** plant‐mediated CH_4_ flux, root exudates, root traits, soil redox potential, spatio‐temporal variability, vegetation shifts

## Abstract

Amplified Arctic warming can induce strong ecosystem changes with adverse climate feedbacks through greenhouse gas (GHG) release. Shifting plant species and traits with permafrost thaw may contribute to the permafrost carbon feedback. How vegetation dynamics in thawing permafrost systems affect GHG release and how this varies with season, plant species, and soil conditions is poorly understood. Here, we assessed GHG emissions, redox potentials, and geochemical signatures as well as the carbon input in the form of root exudation along a vegetation density gradient and a permafrost thaw gradient over a growing season in Stordalen mire, Sweden. Ecosystem respiration and CH_4_ emissions increased along the thaw gradient from bog to fen, possibly due to high graminoid root carbon release rates into an anoxic soil, fuelling fast organic matter oxidation and lowering redox potentials to enhance methanogenesis. CH_4_ emissions increased seven‐fold with increasing graminoid cover compared to non‐vascular plant controls in the thawed soil. Plants may mediate CH_4_ transport, which was responsible for 80% of the graminoid‐induced increase in CH_4_ emissions in the bog environment. In the fen environment, graminoid root carbon release stimulated CH_4_ formation, which dominated by contributing 70% of the graminoid‐induced increase. Overall, photosynthesis‐related CO_2_ fixation was substantial in the early and peak growing season, but when expressed as CO_2_ equivalents, CH_4_ release offset this uptake, resulting in net positive radiative forcings from graminoid‐vegetated thawed soils throughout the growing season. Graminoids increased the net CO_2_‐equivalent flux up to 8.9‐fold compared to non‐vascular plant locations with the strongest forcing toward late season in graminoid‐vegetated fens. Our study showcases how fine‐scaled, plant‐mediated processes differently contribute to GHG emissions across a thawed bog and fen soil and how the time of growing season can overprint these effects to determine whether the system is a net GHG source or sink.

## Graphical abstract

Field study investigating whether and to which degree graminoids further reduce soil redox and lead to more greenhouse gas emissions from thawing permafrost soil on top of hydrology and anoxia effects. Graminoids, thus, contribute to thawed permafrost soils turning to C sources rather than sinks toward the end of the growing season.

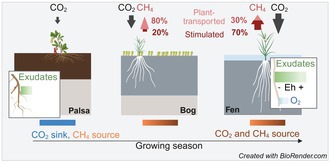

## 1 Introduction

Organic carbon‐rich permafrost peatlands are becoming increasingly vulnerable to destabilization due to Arctic warming. Thawing permafrost can drastically alter local hydrology site‐specifically, leading to either improved drainage or waterlogging and associated changes in water table position (Magnússon et al. 2020). In the latter case, oxic, drained, ice‐underlain permafrost peatland soils, such as palsas, thaw and transition into anoxic, ombrotrophic bogs, and minerotrophic fens (Christensen 2024; Johansson et al. 2006; Leppiniemi et al. 2022). Thaw exposes previously frozen organic carbon (OC) to microbial decomposition, potentially releasing potent greenhouse gases (GHG) CO_2_ and CH_4_ (Patzner et al. 2020). Despite more CO_2_ being emitted from soils, CH_4_ is a critical GHG in thawing peatland systems, given its 100‐year global warming potential of ~27 times the potential of CO_2_ (IPCC 2023). Recent advances in high‐temporal‐resolution monitoring have improved our understanding of CO_2_ and CH_4_ fluxes in permafrost ecosystems (Arora et al. 2019; Dafflon et al. 2017; Morin 2019; Treat et al. 2024). Nonetheless, mechanistic understanding of fine‐scale controls on GHG emissions across the growing season and particularly the contribution of vegetation‐microorganism interactions remains limited (Treat et al. 2024; Riquelme Del Río et al. 2024; Voigt et al. 2019).

Thaw and waterlogging can drive a shift in dominant plant species from slow‐growing, small shrubs to taller, more productive graminoids (Malhotra and Roulet 2015; Varner et al. 2022). These plant types differ in their phenological and physiological plant characteristics, hereafter plant traits (Iversen et al. 2015), which can directly and indirectly affect GHG production, consumption, and transport (Määttä and Malhotra 2024). These plant species‐specific traits include root exudation of carbon (C), oxygen (O_2_) release, and plant‐mediated CH_4_ transport (Jentzsch et al. 2024b; Williams et al. 2021; Määttä and Malhotra 2024).

Root exudates consist of labile C compounds which can easily be oxidized by microorganisms forming CO_2_, even in organic‐rich soils like permafrost peats (Waldo et al. 2019; Wild et al. 2023). Only a few studies have focused on how much and what type of C is released by different permafrost plants species at different time points during the growing season (Oburger and Jones 2018; Wegner et al. 2025). Both root‐released C and resulting CO_2_ serve as key substrates for CH_4_ production by methanogens (Waldo et al. 2019; Määttä and Malhotra 2024). This CH_4_ can either be oxidized by methanotrophs or be transported in the soil via three pathways: diffusion, ebullition and transport within plants (Bastviken et al. 2023). This is possible as graminoids growing in waterlogged bogs and fens feature adaptive root tissues, that is, aerenchyma tissues, enabling the bidirectional transport of gases between atmosphere and soil. For example, plant‐mediated CH_4_ transport allows CH_4_ produced in anoxic soils to move directly through plant tissues to the atmosphere, which was found to be an important process in waterlogged peatlands (Jentzsch et al. 2024b; Korrensalo et al. 2021). In the opposite direction, graminoids release photosynthetically derived and atmospheric O_2_ into their anoxic rhizosphere through radial O_2_ loss (ROL) (Laanbroek 2010; Iversen et al. 2015). Depending on local conditions, ROL affects microbial C utilization by modulating the balance between exudate‐fueled anaerobic processes and O_2_‐supported oxidation (Angle et al. 2017; Wilmoth et al. 2021; Mollenkopf et al. 2026). Collectively, these described plant traits should affect the redox potential of the soil, though this and impacts on GHG emissions have not been investigated systematically yet.

The soil redox potential (*E*_h_) reflects spatial and temporal variations in microbial metabolisms and geochemical processes. The redox ladder describes how microorganisms sequentially use different terminal electron acceptors (TEAs) from O_2_ to CO_2_ based on their relative energy yield under anoxic conditions (e.g., O_2_ > ${\mathrm{NO}}_{3}^{-}$ > Fe(III) > ${\mathrm{SO}}_{4}^{2-}$ > CO_2_). While CO_2_ production occurs across wide redox potentials (Yu et al. 2007), methanogenesis dominates under reduced soil conditions (Perryman et al. 2020). Overlapping redox zones make it difficult to predict CH_4_ emissions. Permafrost peatlands feature sharp redox gradients controlled by soil geochemistry (Milesi 2024; Perryman et al. 2020), TEA availability, OC complexity originating from dominating plant groups (Rupp et al. 2019), and hydrology (Perryman et al. 2020; Street et al. 2016). As such, the position of the water table holds primary control over soil redox conditions in peatlands (Seybold et al. 2002), as it governs O_2_ and substrate availability and the trade‐off between aerobic and anaerobic microbial processes (Fiedler and Sommer 2004).

Season also influences plant traits and GHG fluxes in permafrost peatlands (Bäckstrand et al. 2010; Christensen et al. 2003). During the growing season, increased temperatures and longer days stimulate photosynthesis and root C and O_2_ exudation, making peatlands strong CO_2_ sinks. This may cause a drop in redox, indicating that methanogenesis is fueled and aerenchyma pathways are active, leading to sharp CH_4_ emission peaks in mid‐ to late summer (Bäckstrand et al. 2010; Jentzsch et al. 2024b). Given the high warming potential of CH_4_, the active growing season is expected to become a net GHG source in the future (Bäckstrand et al. 2010; Varner et al. 2022). Plant activity was shown to correlate with CH_4_ fluxes year‐round (Järvi‐Laturi et al. 2025). Thus, the late growing season, where plants senesce, could be an important GHG‐emitting phase (Liu et al. 2022). Here, CO_2_ uptake drastically declines, yet respiration and methanogenesis could be sustained by decaying plant litter while passive gas transport through persistent aerenchyma contributes to sustained CH_4_ emissions (Jentzsch et al. 2024b). Thus, vegetation is debated to play a central role in seasonal GHG emission dynamics (Jentzsch et al. 2024b; Johansson et al. 2006). Knowing that all discussed plant traits undergo seasonality, and likely to a different extent and timing (Jentzsch et al. 2024b; Korrensalo et al. 2021), predictions of ecosystem‐level GHG responses to ongoing warming are difficult. Thus, a detailed understanding of how plant traits respond to environmental and seasonal changes and affect soil redox seasonally is needed to better predict the permafrost C feedback.

Previous studies have shown that vascular plant species can substantially influence C turnover and enhance CH_4_ emissions in wetland ecosystems, mainly through effects on labile substrate supply and plant‐mediated CH_4_ transport (Jentzsch et al. 2024b, 2024a; Korrensalo et al. 2021; Ström and Christensen 2007; Ström et al. 2003, 2005, 2012; Varner et al. 2022). Building on previous work, this study aims to extend the plant trait‐based perspective by directly quantifying natural root exudation across the growing season along a permafrost thaw gradient spanning palsa, bog, and fen. By integrating root exudation with depth‐resolved redox profiles, porewater and soil geochemical measurements, and plant‐mediated CH_4_ transport, this work provides a mechanistic framework linking vegetation change upon thaw to shifts in belowground C processing, redox potentials, and CH_4_ emission pathways.

Specifically, how graminoid expansion upon thaw (at the expense of shrub‐dominated palsas) shapes GHG fluxes across the growing season and how these effects are controlled by root carbon exudation and plant‐mediated CH_4_ transport were assessed. Thus, we hypothesized:

1. Graminoid presence in thawed soils, unlike shrubs in palsa soils, increases CO_2_ fluxes and soil CH_4_ emissions.
2. The relative contributions of graminoid root C exudation and plant‐mediated CH_4_ transport vary between bog and fen as well as with season.

At a representative peatland complex in Stordalen, Abisko, Sweden, thaw‐stage‐specific root C exudation, redox potential, and porewater geochemistry were quantified and correlated to CO_2_ and CH_4_ fluxes across the growing season. Sampling was conducted in shrub/graminoid‐vegetated and nearby non‐vascular plant locations along a palsa‐bog‐fen gradient, with measurements during early, peak, and late growing season. To control for CO_2_ fixation effects on GHG flux assessments during photosynthesis, opaque chamber GHG assessments were contrasted against transparent chamber systems. To separate plant‐transported from soil‐derived CH_4_ emissions, a dual‐chamber approach separating plant from soil GHG emissions was utilized. Together, this design allows us to attribute thaw stage and seasonal shifts in net CO_2_ exchange and CH_4_ emissions to specific plant and root traits, improving the understanding of vegetation‐mediated permafrost C feedbacks.

## 2 Materials and Methods

### 2.1 Field Site and Study Concept

Stordalen mire (68°20′ N, 19°03′ E) close to Abisko, Sweden, is a well‐studied permafrost peatland complex where thaw resulted in the formation of three sub‐habitats in close proximity: a well‐drained intact permafrost soil dominated by shrubs, that is, *Andromeda polifolia* L., *Betula nana* L., that upon thaw slopes down to a wet *Sphagnum* ssp. dominated bog and finally to a fully inundated fen covered by grasses and sedges, that is, *Eriophorum vaginatum* L., *Eriophorum angustifolium* Honck., *Carex* ssp. (Figure S1). Here, “thaw stages” refers to these three hydrologically contrasting habitat types (palsa, bog, fen) within the Stordalen mosaic. For each biological replicate, a co‐located palsa‐bog‐fen transition within the same local palsa complex (palsa → adjacent bog → adjacent fen) was selected, rather than sampling spatially unrelated patches across the mire. Detailed field‐site characteristics are described elsewhere (Freire‐Zapata et al. 2024). The plant groups differ in major phenological and physiological characteristics: shrubs emerge from snow cover earlier in the growing season, initiating photosynthesis before graminoids, but are slow‐growing with lower root turnover. In contrast, graminoids (grasses and sedges) typically have later seasonal growth initiation but higher plant productivity and greater root biomass (Blume‐Werry et al. 2019; Iversen et al. 2015).

**FIGURE 1:**
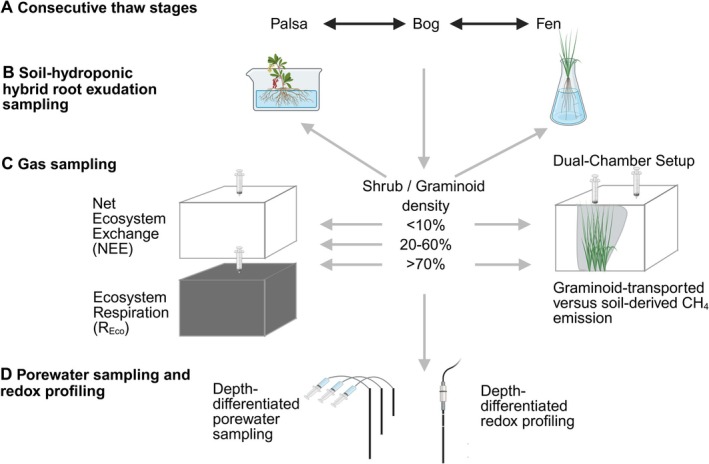
Conceptual overview of the study design and sampling workflow across thaw habitats and vegetation densities. (A) Consecutive thaw‐associated habitats at Stordalen mire (palsa, bog, fen). (B) Shrub and graminoid root exudation collected and quantified using a hydroponic (hybrid) root exudate collection approach. (C) Greenhouse gas fluxes measured across vegetation density classes using static chambers to quantify net ecosystem exchange (NEE; transparent chambers allowing photosynthesis) and ecosystem respiration (*R*_Eco_; opaque chambers stopping photosynthesis and its impact to the soil), and a dual‐chamber setup used to partition graminoid‐mediated gas transport (bag around plant) from soil‐mediated gas transport (bag above soil without the plant). The arrows indicate from which plant density class each type of greenhouse gas sampling was taken. (D) Depth‐resolved porewater sampling and redox (*E*_h_) profiling conducted at corresponding plots to link greenhouse gas fluxes to geochemical conditions. Created with biorender.com.

To study the role of different plant traits on soil biogeochemistry and the production and release of GHG, above‐ and below‐ground sampling was performed across a vegetation density gradient in each thaw stage (Figures S1 and S2) as done by others (Biasi et al. 2014). This allows for a relative comparison of plant trait‐related processes that are normalized to a non‐shrub/non‐sedge location within each stage. Locations were chosen based on the presence and absence of graminoids/shrubs. Within each habitat, adjacent microsites representing a vascular plant cover gradient as a proxy for variation in aboveground biomass/greenness were selected. Collars were assigned to three categories based on the estimated vascular plant cover within the collar area: non‐vascular plants (“baseline”; moss/lichen may be present in palsa and bog, waterlogged soil in fen), sparsely vegetated (20%–60%), and densely vegetated (> 70%). For clarity in the main text, we focused on the two endmembers, baseline and densely vegetated, and additional density information remaining in the Supporting Information (Figures S2–S4, S6, and S7; Tables S1 and S2). Belowground (geochemistry, soil redox potential, C input by root‐released C exudation) measurements were performed at the same locations after gas sampling. An overview of the sampling design and measurements across habitats and vegetation density classes is shown in Figure 1. Sampling was performed at three different timepoints (June/July, August, and September) within the 2023 season. Plant‐mediated gas transport was assessed in August 2024.

### 2.2 Field Sampling

Gas sampling across the vegetation gradient was performed in 2–3 biological (different locations) (Figures S1 and S2, Table S1) and 3 temporal replicates (same locations, but other times of the day), each using static gas flux chambers in the light (transparent chambers; addressing net ecosystem exchange [NEE]) and the dark (opaque chambers; addressing ecosystem respiration [*R*_Eco_]). For opaque chamber measurements, the chambers were covered with black plastic bags to avoid any light penetration and thus allow for a comparison of photosynthetically active and inactive vegetation. Each thaw stage was sampled within 1 day, and gas sampling was scheduled for three consecutive days with similar weather conditions. Chambers were placed on metal collars (~3 kg) to ensure gas‐tightness. Collars were inserted in the soil (5–10 cm deep) at least 3–16 h before gas flux measurements to ensure re‐equilibration. Collars were installed by gently pressing them into the peat (without excavating a trench) and remained in place during all replicate measurements that day. Data loggers (TFA Dostmann, AirCO_2_ntrol 5000) measuring CO_2_, temperature, and humidity every 30 s were installed in the flux chamber for high‐resolution, real‐time measurements, which also allowed checking gas‐tightness of the chambers. Gas flux through incubation was started by setting the chamber on top of the collar. Three gas samples (30 mL) were taken through the sampling port using a gas‐tight syringe within a 25 min incubation period and injected into pre‐N_2_‐flushed exetainers. Water table depth (relative to the peat surface) was measured inside each collar after GHG sampling to avoid disturbing flux measurements and is summarized in Table S1.

To separate plant‐mediated gas transport through the roots and plant itself from other effects of present vegetation on emitted gases, a dual‐chamber gas sampling design was used similar to Ge et al. (2024). For this, the static gas flux chambers were additionally equipped with a gas tight plastic bag that was encased around the plants. The bag was equipped with an additional sampling port leading through the flux chamber. Thus, it was possible to sample two ports simultaneously, one covering gas exchange of the soil around the plant and one covering the plant itself. These measurements were performed in August 2024.

Porewater sampling was performed using lysimeters (Makrorhizons, Rhizosphere Research Products, Netherlands) at three different depths (~20, ~35, and ~45 cm). After installing the Rhizons to the desired depths, pre‐N_2_‐flushed syringes were attached and porewater was pulled by applying a vacuum. Approximately 10 mL of porewater was pulled and aliquoted for dissolved OC (DOC), dissolved nitrogen (N) species, iron (Fe), and other elements (stabilized in 1 M HCl), and porewater gas. Porewater pH was measured in the field when possible or at the research station on the same day. Aliquoted samples were either cooled (4°C, Fe and other elements, stabilized by acidification in 1 M HCl) or frozen (−20°C, DOC and N). For porewater gas, 3 mL of porewater was injected into a pre‐N_2_‐flushed butyl‐stoppered 10 mL headspace vial and shaken for 3 min. 3 mL of gas was taken from the headspace and injected into a pre‐N_2_‐flushed exetainer. Given too dry soils in June/July, porewater sampling in palsa locations was only possible in August and September.

Redox potentials were measured in situ and simultaneously at four different depths (10, 20, 30, and 40 cm, temperature sensor at 25 cm) using an ORP‐40‐4‐A redox probe with an integrated Ag/AgCl reference electrode (SWAP Instruments, Netherlands). The quality of the probe was checked in advance using known standards. The redox probe was pushed into the soil and reads were taken after 15 min.

Representative plants ( *A. polifolia* for the palsa and *E. angustifolium/Carex* ssp. [hereafter termed graminoids] for the thawed soil) were taken from close to the sampling locations, immediately transported to the research station and used for sampling of root‐released C by an approach modified from Oburger and Jones (2018). In brief, after washing the roots and a 16‐h pre‐incubation in tap water, root‐released OC was collected for 3 h in a hydroponic solution (artificial rainwater containing 5.6 mg L^−1^ NaNO_3_, 2.4 mg L^−1^ NaHCO_3_, 2.8 mg L^−1^ K_2_SO_4_ and 136 mg L^−1^ CaSO_4_) under a plant lamp, and wet and dry plant and root biomass was determined afterward for normalization. The collected solution was sterile filtered (0.22 μm, Millipore Steritop, Merck) and frozen at −20°C.

### 2.3 Gas Analyses

CO_2_ and CH_4_ were analyzed using a gas chromatograph (TRACE 1310, Thermo Fisher Scientific, Waltham, Massachusetts, MA, USA) equipped with two pulsed discharge ionization detectors (PDD). Due to instrument issues, gas samples from the June/July campaign were measured on a different instrument (Bruker Daltonic, Germany) equipped with a flame ionization detector (FID) for CH_4_ concentration and an electron capture detector (ECD) for CO_2_ concentration using the same standards. Gas emission rates were calculated based on a linear change in chamber gas concentration over time and normalized to the chamber area. In the dual‐chamber gas sampling method, the area covered by the bag enclosing the plant was estimated by measuring two perpendicular diameters after placing the bag over the plant. The volume was then calculated using this area together with the known height of the chambers, as the bags were attached directly to the Plexiglas top. CO_2_‐eq is reported as a GWP100‐weighted CO_2_‐equivalent flux for comparing gas‐exchange magnitude and composition and is not interpreted as radiative forcing unless expressed as a change relative to a baseline or between treatments (Neubauer 2021). Measured parts per million (ppm) concentrations were corrected for temperature and atmospheric pressure using the ideal gas law. Gas fluxes were included when the *R* ^2^ of the linear change in headspace gas concentration over time was > 0.8. To compare CO_2_ and CH_4_ contributions, a weighted CO_2_‐equivalent flux (CO_2_‐eq) was calculated by converting CH_4_ using a factor of 27 (IPCC 2023) as a 100‐year global warming potential (GWP) factor and summing with CO_2_.

### 2.4 Root‐Released C Analyses

The amount of root‐released C and N was quantified at least four biological replicates and technical triplicates using a multi N/C 2100 (Analytic Jena, Germany) and normalized to the root dry weight. To obtain information on the amount of released C input via roots per g soil, Monteux et al. (2018) provided data on the mass of root per soil volume of the two representative plant groups in a neighboring peatland. The composition of the released C was characterized for major metabolites (Table S2) using gas chromatography coupled to mass spectrometry GC × MS (Shimadzu GC/MS TQ 8040). Metabolites were analyzed either by headspace injection or as liquid samples after derivatization and addition of octanol or ^13^C‐glucose as internal standard, respectively (see Methods S1 for details). Metabolite concentrations were quantified with an 8‐point external calibration containing standards of all targeted analytes, ranging from 20 to 10^7^ pmol (details see Text S1). R (Team 2024) was used to calculate relative peak areas and the concentrations of metabolites.

### 2.5 Field Porewater Analyses

Porewater pH was determined using a field pH probe (Multi 3430 Set, WTW, USA). Porewater DOC and N species (${\mathrm{NH}}_{4}^{+}$, ${\mathrm{NH}}_{3}^{-}$) were quantified using a multi N/C 2100 (Analytik Jena, Germany) and using continuous Flow Injection Analysis (AA3, Seal analytical; UK). Fe concentration and speciation were determined spectrophotometrically using the ferrozine assay (Stookey 1970). Acidified porewater was diluted in nitric acid (trace metal grade) and analyzed in technical triplicates for the various elements on an Agilent 7900 ICP‐MS (Agilent Technologies, USA). Certified quality controls at different concentrations were measured every 20th sample. Rhodium as an internal standard (product# 5188‐6525, Agilent Technologies, USA) was used for corrections of instrument drift.

### 2.6 Data Analyses and Presentation

Plot‐scale chamber GHG flux data were compared to ecosystem‐level Eddy Covariance (EC) flux data obtained from the Integrated Carbon Observatory System (ICOS) portal (ICOS, Sweden; the full dataset citations and persistent identifiers are provided in the References). EC fluxes were averaged across multiple years, and the plot‐scaled sampled timings for comparison. EC footprint relative fractions were determined based on a previously published footprint model (Laasonen et al. 2025). To assess how representative the plot‐scale chamber measurements are at the ecosystem scale, these flux measurements were multiplied by habitat‐specific footprint fractions and summed, resulting in a footprint‐weighted average chamber flux (Equation 1).

(1) $${\text{Flux}}_{\text{chamber},\text{weighted}}=\sum_{i}\left({\text{Flux}}_{\text{chamber},i}\times f_{i}\right)$$

where Flux_chamber,weighted_ is the footprint‐weighted chamber flux, directly comparable to ecosystem‐level Eddy Covariance (EC) flux, Flux_chamber,*i*_ is the measured plot‐scale chamber flux for each habitat type (*i*: palsa, bog, fen), *f*_*i*_ represents the corresponding EC footprint fraction for each habitat type. Finally, this weighted chamber flux was compared to EC fluxes (only including fluxes between 8 a.m. and 8 p.m.) averaged over the same sampling dates. For comparison, both the average EC fluxes across several years (2017, 2019, 2020, 2021), as well as only 2023, were used.

Geochemical data are presented in the supplement (Figures S5–S7, Table S2). Changes in redox potential and porewater chemistry are shown across different vegetation densities in the bog. Palsa and fen had no substantial difference based on vegetation density (see data in Table S2), in the case of fen, likely due to hydrological conditions resulting in strong porewater mixing (Olefeldt and Roulet 2014).

Mean values and one standard deviation (1SD) or standard error (SE) were calculated for all data sets. Changes were calculated for most datasets and presented in percent, and when exceeding 100% as fold change. Absolute CO_2_ and CH_4_ flux values are provided in Tables S3 and S4 and are additionally available in the associated data repository. Negative fluxes are indicated as negative values and referred to as net uptake, whereas positive fluxes are referred to gas emissions. A univariate general linear model (GLM) was used to assess the effects of soil type (Palsa, Bog, Fen), vegetation condition (baseline, vegetated), and season (June, August, September) on gas emissions, including all main effects and interactions. Pairwise comparisons were performed using estimated marginal means with least significant difference (LSD) post hoc tests. Significance was set at *α* = 0.05. GLMs were calculated using IBM SPSS Statistics (Version 27). Additionally, unpaired significance tests were conducted to evaluate specific hypotheses between selected group combinations using R (Team 2024). Significance was set to *α* = 0.05. Normality of the distribution was assessed with a Shapiro–Wilk test. Student's *t*‐tests were then applied when both groups were distributed normally. Otherwise, a Wilcoxon rank‐sum test was used.

Principal component analysis (PCA) was performed to identify key factors influencing peatland biogeochemistry across varying plant density and sampling seasons. Analyses for PCA were conducted using R (Team 2024). Variables were standardized (scaled and centered) before PCA. Water table depth was excluded from the PCA because it is related to redox potential and including both could overweigh the same hydrology redox gradient (Fiedler and Sommer 2004; Seybold et al. 2002). PCA calculations and visualizations, including confidence ellipses (80%) and variable loadings, were implemented using the *prcomp* function and the *ggplot2*, *ggrepel*, and *factoextra* packages (Kassambara and Mundt 2016; Slowikowski et al. 2021; Wickham 2016).

## 3 Results

In the following, vegetated plots refer to shrub‐dominated palsas and graminoid‐dominated bog/fens (> 70% cover), while baseline plots lack vascular plants but feature mosses and lichen. Data for a sparsely vegetated location (20%–60% cover) is presented in the Supporting Information (Figures S3, S4, S6, and S7). Additionally, opaque chamber CO_2_ flux refers to ecosystem respiration (*R*_Eco_) excluding plant photosynthetic effects and enabling process‐based understanding of root influences. Transparent chamber CO_2_ fluxes refer to net ecosystem exchange (NEE), including plant photosynthesis, enabling understanding of the net effects of plants on GHG fluxes. The three habitats also differed systematically in hydrology: palsa sites were drained, bog sites had near‐surface water tables, and fen sites were persistently inundated (Table S1). These hydrological differences provide the boundary conditions for redox zonation and CH_4_ cycling evaluated below.

### 3.1 Greenhouse Gas Dynamics Affected by Thaw‐Specific Plant Communities

**FIGURE 2:**
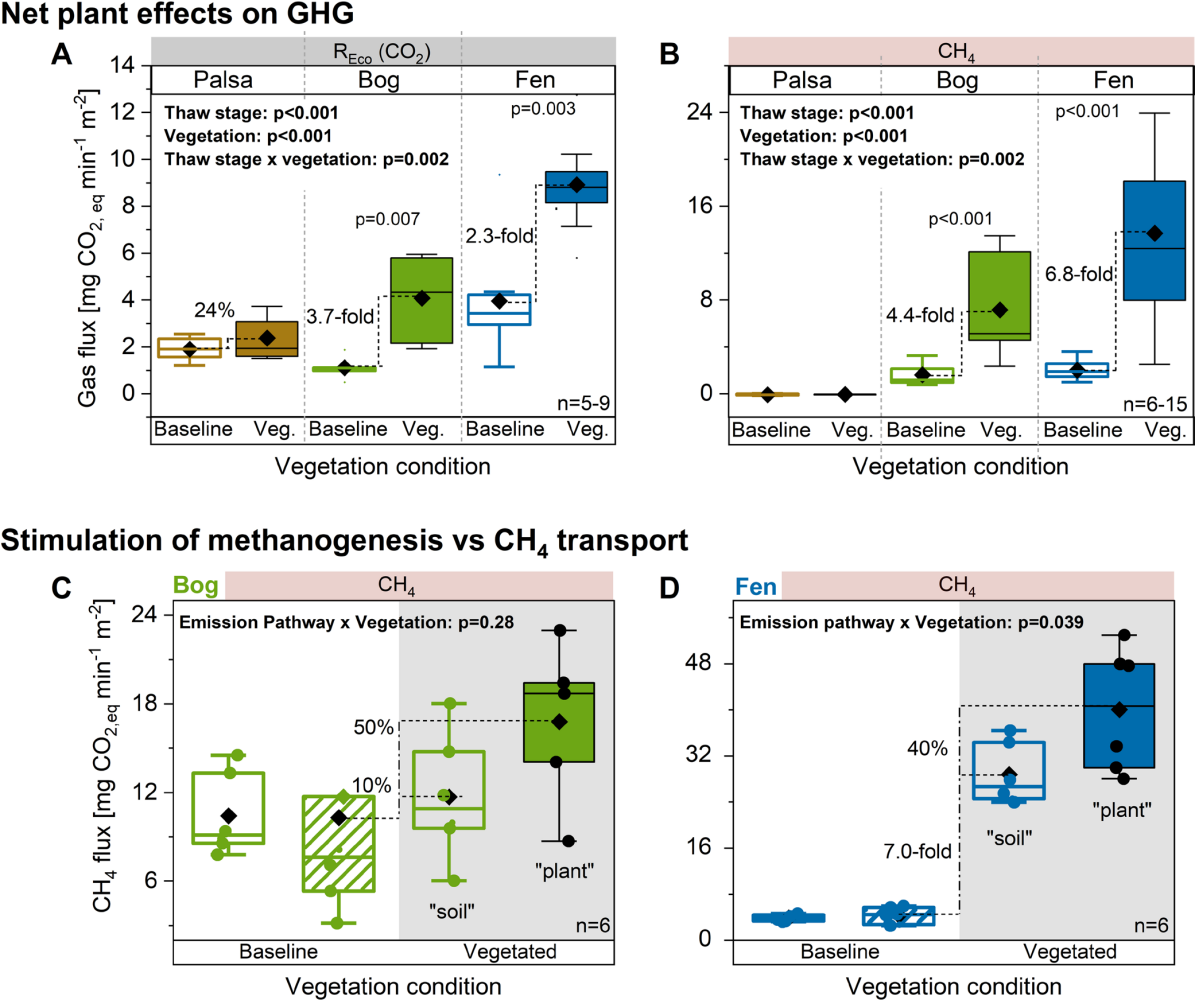
Greenhouse gas dynamics from baseline (non‐vascular plant reference) and vegetated plots along permafrost thaw stages. Baseline plots were dominated by non‐vascular vegetation (mosses/lichens) or waterlogged soil. (A) CO_2_ (*R*_Eco_) and (B) CH_4_ emissions across baseline and shrub/graminoid vegetated palsa, bog and fen stages in June/July and August. To isolate soil and root respiration in the presence of plants, photosynthetic CO_2_ fixation was excluded using opaque chambers. The contribution of plant‐mediated CH_4_ transport versus soil‐derived CH_4_ emissions was examined in (C) bog and (D) fen stages using a dual‐chamber approach: CH_4_ diffusion through the soil was measured with gas flux chamber (empty boxes), while plant‐transported CH_4_ was captured in gas‐tight bags placed over vegetation within the chambers (filled box). Bags were also placed in baseline plots as controls (empty and striped box). Statistical significance is assessed with a two‐sided *t*‐test and included in the graph when *p* < 0.1. General linear model assessing the effect of thaw stage/emission pathway, vegetation density, and their interaction, statistical analysis is detailed in Tables S6–S11. Black diamonds represent the mean, the central box line the median, boxes indicate interquartile range (25th–75th percentiles), whiskers denote ±1.5× IQR.

To distinguish the impact of vegetation presence on *R*_Eco_ (CO_2_) and CH_4_ emissions, GHG fluxes were quantified in baseline and shrub/graminoid‐vegetated locations across palsa, bog, and fen (Figure 2A,B). Univariate generalized linear models confirmed significant effects of thaw stage, vegetation presence, and their interaction on *R*_Eco_ and CH_4_ emissions (Tables S5 and S6). For the *R*_Eco_, graminoids increased emissions 3.7‐fold in bog and 2.3‐fold in fen but shrubs had no significant impact in palsa (Figure 2A). CH_4_ emissions were minimal in baseline bog and fen. The presence of graminoids significantly increased CH_4_ emissions 4.4‐fold in bog and 6.8‐fold in fen (Figure 2B).

To assess the contribution of plant‐mediated CH_4_ transport versus gas diffusion through the soil (collectively termed the emission pathway), a dual‐chamber method (see Section 2) was employed. A significant interaction between vegetation presence and emission pathway was found in fen, but not in bog (Tables S8 and S9). In detail, for fen, graminoid presence increased soil‐diffused CH_4_ emissions 7‐fold and plant‐transported CH_4_ emissions 9.8‐fold (Figure 2D). In contrast, for bog, graminoid presence moderately increased plant‐transported CH_4_ emissions by 62%, while soil‐diffused CH_4_ stimulation remained largely unaffected (Figure 2C). For CO_2_, no distinct transport‐related effects were observed (Figure S8, Table S10).

Seasonal CO_2_ to CH_4_ ratios were used to determine shifts in the relative contribution of CH_4_ compared to CO_2_ emissions during thaw progression (Figure S3F,G, Table S11). In fen baseline, CO_2_:CH_4_ ratios declined by 86% from June to September. Ratios did not significantly differ between baseline and graminoid vegetated plots at individual time points.

Porewater GHG concentrations increased consistently with soil depth (Figure S4). Porewater CO_2_ concentrations followed the order bog > fen > palsa, whereas porewater CH_4_ was most concentrated in fen, followed by bog and palsa. Neither graminoid presence nor season significantly affected porewater CH_4_ concentrations, whereas graminoid presence decreased and season increased porewater CO_2_ concentrations.

### 3.2 Season‐Dependent Influence of Vascular Plant Functional Type on Net Ecosystem Exchange and CH_4_ Emission

**FIGURE 3:**
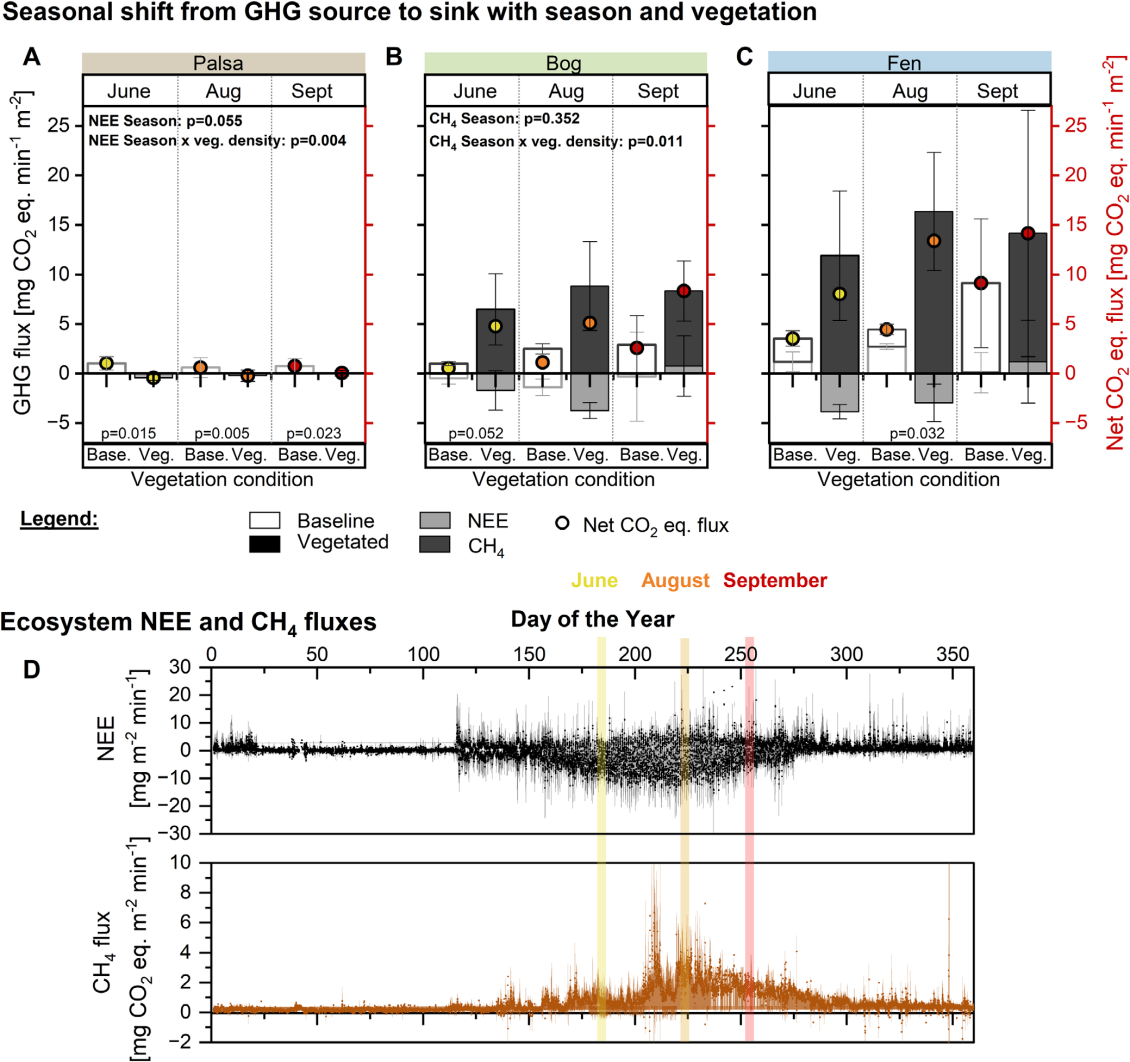
Seasonal greenhouse gas fluxes with vegetation impacts across thaw stages. (A) Palsa, (B) bog, and (C) fen CO_2_ and CH_4_ emissions are shown for June/July, August, and September, measured in transparent chambers at baseline (non‐vascular plant reference, empty bars) and vegetated (filled bars) sites. Baseline plots were dominated by non‐vascular vegetation (mosses/lichens) or waterlogged soil. Corresponding net CO_2_ equivalent fluxes, considering both CO_2_ and CH_4_ contributions, are depicted with colored circles on the right *y* axis, *n* = 4–9. Statistical significance is assessed with a two‐sided t‐test and included in the graph when *p* < 0.1. Full statistical analysis in Tables S12 and S13. (D) Half‐hourly flux tower data of CO_2_ and CH_4_ emission spanning multiple years (2017, 2019, 2020, 2021, 2023) (data obtained from Integrated Carbon Observatory System, Sweden [ICOS Sweden] 2019, 2021, 2022, 2023; Lundin et al. 2025). Colored bars indicate the timing of sampling campaigns corresponding to panels A–C. Bar plots show the mean ± 1SD.

Flux data differentiating baseline and shrub/graminoid vegetated areas were collected at specific growing season time points (Figure 3A–C). Negative NEE indicates net CO_2_ uptake by the ecosystem from the atmosphere, whereas positive NEE indicates net CO_2_ release to the atmosphere. A univariate generalized linear model indicated that (i) season affected NEE but not CH_4_ emissions, and (ii) vascular plant presence (shrubs in palsa and graminoids in bog/fen) and season significantly interacted, affecting both NEE and CH_4_ emissions (Figure 2A,B, Tables S12 and S13). At the ecosystem scale, EC flux tower data from Stordalen mire provided by ICOS Sweden illustrate typical summer negative NEE and positive CH_4_ fluxes (Figure 3D). Weighting plot‐scale chamber fluxes by the habitat fractions within the EC tower footprint derived from Laasonen et al. (2025) produced a footprint‐integrated flux estimate that better matched tower‐scale NEE and CH_4_ observations than either endmember scenario alone (Figure S9).

In palsa, NEE at baseline sites became less positive across the growing season, and CH_4_ emissions remained negligible (Figure 3A). Shrub vegetation caused a small but significant shift toward less negative NEE to net‐zero NEE by September.

Baseline bog sites, always moss‐covered, consistently had a negative NEE (Figure 3B). Graminoid presence enhanced CO_2_ uptake (more negative NEE) 2.7‐fold in August. In September, graminoid presence turned bogs to positive NEE. CH_4_ emissions in baseline sites increased ~3‐fold from early to late growing season. Graminoid presence consistently amplified CH_4_ emissions, especially in June, where fluxes increased 6.4‐fold relative to baseline bog. Overall, graminoid vegetation resulted in a positive radiative balance in the early and mid‐growing season and turned to an overall GHG source in September.

Baseline fen sites had positive NEE in the early and mid‐growing season, peaking in August at more than twice the June levels, and were net zero NEE in September (Figure 3C). Graminoid presence significantly transformed the fen into negative NEE during June and August only, whereas NEE became positive in September. CH_4_ fluxes from baseline fen increased through the season, with September emissions being 3.9‐fold higher than June emissions. Graminoid presence caused significant 5.1‐fold and 9.4‐fold increases in CH_4_ emissions in June and August, and 40% higher CH_4_ emissions in September. Collectively, these patterns caused the radiative balance to substantially increase in the presence of graminoids.

### 3.3 Thaw Stage Responses to Plants: Root‐Released C, Soil Redox Potentials, and Porewater Geochemistry

**FIGURE 4:**
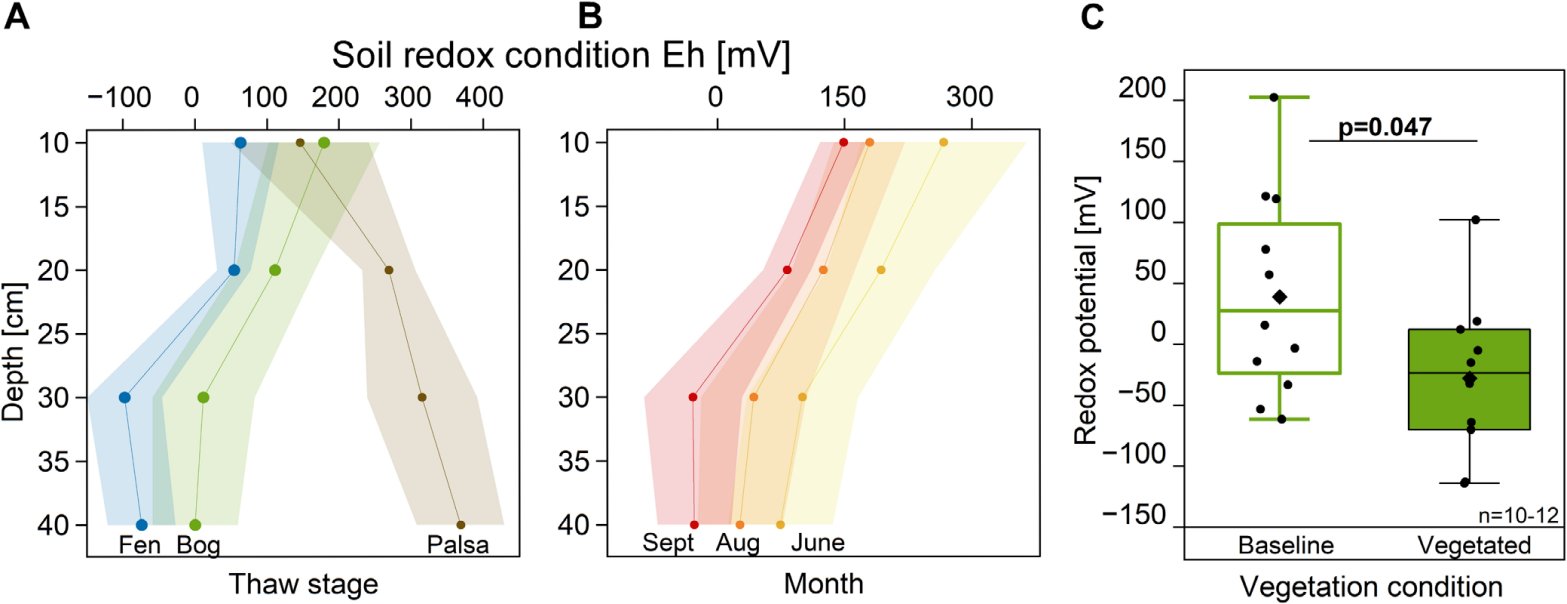
Redox potentials in permafrost thaw stages across growing season and impacted by the presence of vegetation. (A) Depth‐resolved redox potentials in palsa (brown), bog (green), fen (blue). (B) Bog redox potential in June (yellow), August (orange), and September (red). (C) Redox potentials in baseline (non‐vascular plant reference) bog (empty box) and vegetated bog (filled box). *N* = 15–32. Baseline plots were dominated by non‐vascular vegetation (mosses/lichens) or waterlogged soil. Full statistics are provided in detail in Tables S15 and S16. Circles in A and B represent the mean; the shaded area represents 1SD and is interpolated with depths. Black diamonds in C represent the mean, the central box line the median, Boxes indicate interquartile range (25th–75th percentiles), whiskers denote ±1.5× IQR. Statistical significance is assessed with a two‐sided *t*‐test (*p* < 0.05).

Redox potentials differed among thaw stages, seasons, and vegetation (Figure 4, Tables S15 and S16). In palsa, the redox potential remained stable around +300 mV (Figure 4A). In the bog, potentials were significantly lower compared to palsa, decreasing notably with depth. Fen soils had the lowest redox potential overall, reaching minimum values at 30 cm depth. In bog, redox potential significantly declined with advancing growing season (Figure 4B). Graminoid presence in bogs significantly reduced redox potentials from an average of +39 mV in baseline plots to −28 mV in vegetated plots (Figure 4C).

**FIGURE 5:**
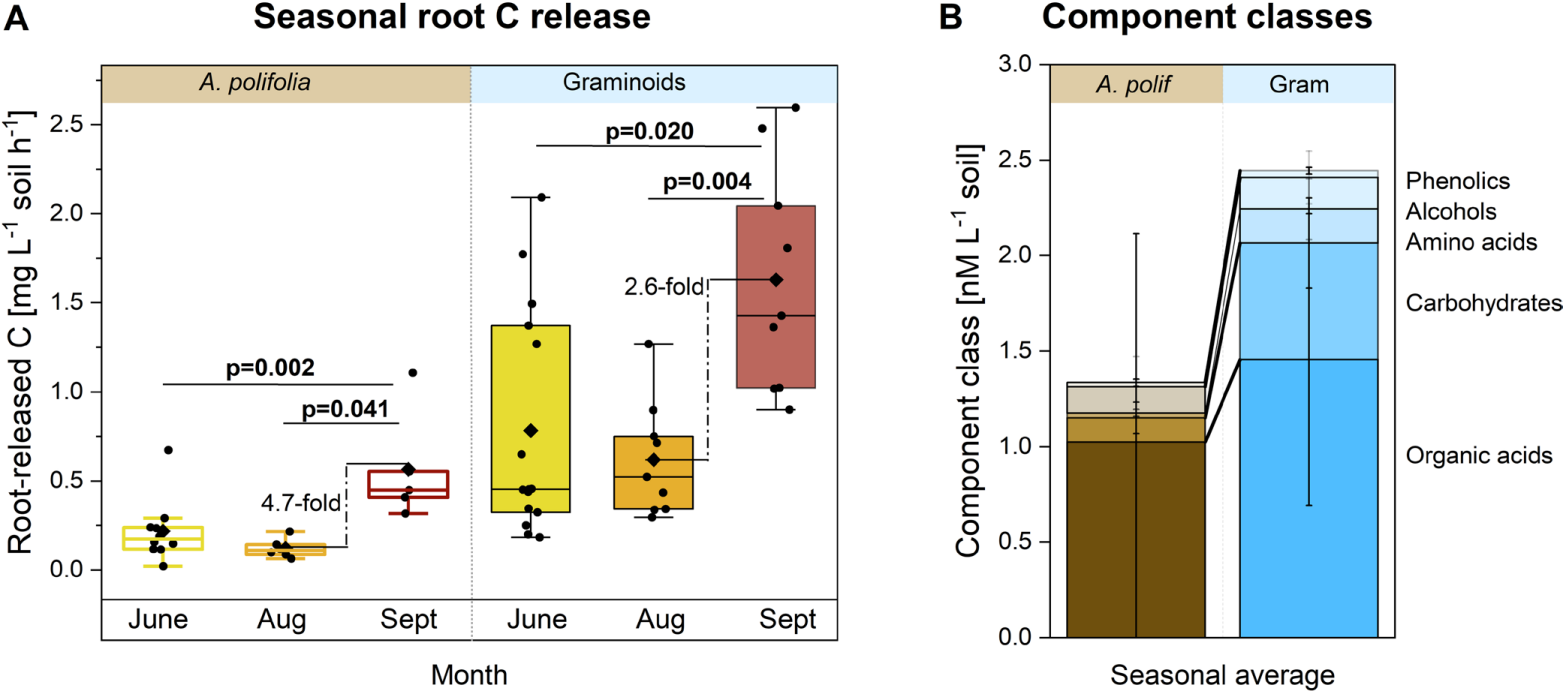
Amounts and composition of root‐released C substrates released from representative plants of palsa and fen. Root‐released C substrates were collected by placing *Andromeda polifolia* sampled from palsa and graminoids sampled from the fen in a hydroponic solution. (A) Amounts of root‐released C collected from *A. polifolia* (brown) and graminoids (blue) in June (yellow), August (orange), and September (red) of the growing season. (B) Component classes of *A. polifolia* and graminoids averaged across the growing season. Mean ± 1SD, *n* = 3–4. A complete list of measured metabolites for each growing season and plant is presented in Figure S10. Statistical analysis is detailed in Table S17. Black diamonds in A represent the mean, the central box line the median, Boxes indicate interquartile range (25th–75th percentiles), whiskers denote ±1.5× IQR. Stacked bar plots in B show mean ± 1SD.

Root C release rates differed significantly between fen graminoids and palsa shrubs (Figure 5A, Table S17). In June, fen graminoids released 3.6 times more C than palsa shrubs. From June to August, C release rates did not change for either graminoids or shrubs, though graminoids consistently released 5 times more C than shrubs. However, by September, C release rates significantly increased 2.6‐fold for graminoids and 4.7‐fold for shrubs compared to August. The composition of released metabolites also significantly differed between plant types (Figure 5B, Figure S10). Graminoid released C was enriched in carbohydrates relative to shrubs.

Soil temperature varied among thaw stages (Table S2). In palsa soils, temperatures averaged 8.3°C ± 1.2°C at 20–30 cm depth with a maximum of 10.1°C ± 3.98°C in August. Bogs exhibited pronounced seasonal variation (8.1°C ± 1.8°C) with the highest temperatures of 11°C ± 1.2°C in August compared to June and September. Fen soil temperature averaged 10.4°C ± 1.0°C at 25 cm, reaching a maximum of 11.7°C ± 0.8°C in August. Water tables were consistently shallow in bogs (0–11 cm below surface) and were slightly shallower in the later growing season (Table S1). Fen sites had deeper water tables (12–23 cm), and baseline locations were generally deeper than vegetated plots, especially in August–September (baseline: 16–20 cm; graminoid vegetated: 12–16 cm). Porewater pH differed between thaw stages (Table S2). Palsa pH decreased from 4.9 to 4.6, bog pH increased from 4.3 to 4.8, and fen pH remained constant around 6.0 across the growing season.

Porewater nutrient stoichiometry differed among thaw stages (Figure S5). Palsa had distinctly higher C:P and N:P ratios compared to the other stages. Bog porewater exhibited the highest C:N ratio, whereas its N:P ratio was comparably low and similar to fen. Fen had the lowest porewater C:N and C:P ratios overall.

Fe and DOC concentrations varied with thaw stage, depth, vegetation density, and season (Figures S6 and S7). Porewater Fe_tot_, Fe(II), and Fe(II)/Fe_tot_ increased with soil depths and were lowest in palsa, highest in bog, and intermediate in fen (Figure S6). The presence of vegetation and increasing growing season increased Fe_(tot)_ and Fe(II).

DOC concentrations tended to increase with depth and were intermediate in palsa, highest in bog, and lowest in fen (Figure S7). Graminoid presence in bogs elevated DOC by 58% compared to baseline sites. Additionally, DOC significantly increased by 69% from June to September.

**FIGURE 6:**
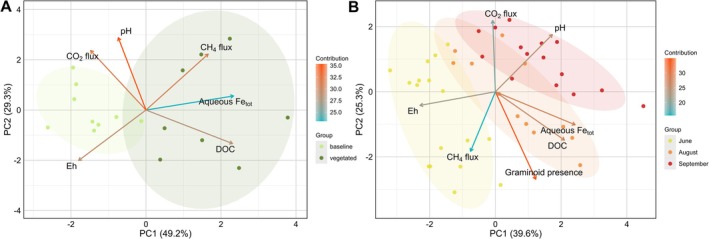
Principal component analysis (PCA) biplots to explain vegetation and growing season impacts on bog peatland biogeochemistry. (A) Separation of bog sites based on graminoid cover (baseline [non‐vascular reference] vs. vegetated) highlights vegetation effects on geochemical properties, and (B) separation of bog sites based on season (June, August, September) distinguishes growing season effects on geochemical properties. Baseline plots were dominated by non‐vascular vegetation (mosses/lichens) or waterlogged soil. Ellipses show 80% confidence intervals around clusters. Loadings represent the direction and strength of each environmental variable's influence on the principal components. Arrow colors reflect the variable's contribution percentage. PCAs for other stages are shown in Figure S11.

To integrate multivariate relationships among GHG fluxes and porewater/redox variables in the bog, a principal component analysis (PCA) was conducted (Figure 6). Baseline and graminoid‐vegetated samples separated primarily along PC1 (49.2% of the variance) (Figure 6A). Response variables DOC, aqueous Fe_tot_, and CH_4_ flux loaded toward positive PC1, while *E*_h_ loaded toward negative PC1/PC2. Separation of growing season months mainly clustered along PC1 (39.6%) with a meaningful contribution of PC2 (25.3%) (Figure 6B). June samples occurred mainly at negative PC1 scores and September samples at positive PC1 scores. DOC and aqueous Fe_tot_ loaded toward positive PC1, whereas Eh and CH_4_ flux loaded toward negative PC1. PCAs for palsa and fen are provided in Figure S11.

## 4 Discussion

Our study demonstrates that graminoids in thawed permafrost soils are primary drivers of seasonal biogeochemical dynamics and GHG emissions at Stordalen mire, Sweden. Functional plant traits, such as root‐released C input and plant‐mediated CH_4_ transport, intensify with thaw and change with the season. Vegetation effects are interpreted within the hydrological setting of each habitat: water table position sets the dominant redox framework, while plant traits (root C inputs and plant‐mediated transport) alter nutrient and electron acceptor availability and supply, redox dynamics, and emission pathways within that redox framework. Together with differences in water table position, these plant traits can maintain thawing permafrost soils as strong CO_2_ sinks in early and peak summer while increasing net CO_2_‐equivalent emissions across the growing season through enhanced CH_4_ emissions, with the strongest forcing often toward late summer.

The discussion is structured around two integrated themes: (1) mechanisms of plant‐mediated GHG dynamics, and (2) the seasonal modulation of these mechanisms.

### 4.1 Mechanisms of Plant‐Mediated GHG Dynamics

Graminoids released more OC into fen and bog soils than shrubs into palsa soil (Figure 5A). Typically, around 20% of photosynthetically fixed C is released as root C exudation (Hütsch et al. 2002). Since fen graminoids are known to exhibit higher photosynthetic activity than shrubs (Ward et al. 2009), graminoids are expected to contribute higher amounts of root‐derived C into the soil. This higher C input stimulated soil microbial activity (Hough et al. 2020), as supported by higher *R*_Eco_ observed in bogs and fens (Laurent et al. 2025) (Figure 2A,B).

Soil properties along the thaw gradient influence the fate of this plant‐released C. In palsa soils, shrub presence *R*_Eco_ by only 24%, as microbial turnover remained low due to lower temperatures and a lower pH, resulting in limited mineralization of released C and SOC to CO_2_ (Wang and Kuzyakov 2024; Wei et al. 2021). Bogs are dominated by *Sphagnum* mosses producing microbial inhibitors like phenolics and uronic acids (Fudyma et al. 2019; Hough et al. 2022), maintained lower pH (4.0–4.5), and exhibited an unfavorable nutrient stoichiometry to microorganisms, higher content of oxidized Fe, and increased DOC concentration (Figures S5–S7). Thus, DOC likely accumulated due to inhibited microbial utilization rather than enhanced enzymatic hydrolysis, reflecting overall low microbial activity (Hough et al. 2020; Wilson et al. 2022). Nonetheless, in the presence of graminoids, bogs showed the highest increase in *R*_Eco_ (up to 3.7‐fold increase relative to baseline; Figure 2A). In fens, where soils were warmer (9°C–13°C at 10 cm), near neutral in pH (~6.5), and richer in N (Figure S10), graminoid presence increased *R*_Eco_ 2.3‐fold compared to baseline sites (Figure 2A). Despite soil conditions in fens being more favorable for microbial activity than in bogs, less of the released C and SOC was respired to CO_2_, likely because lower redox conditions promote CH_4_ production over respiration. Indeed, seasonal CO_2_:CH_4_ emission ratios were significantly lower in fens compared to bogs (Figure S3), suggesting that CO_2_ produced during respiration could have been further reduced to CH_4_ under strongly reducing soil conditions. Consistently, graminoid presence decreased porewater CO_2_ concentrations, supporting enhanced CO_2_ consumption under more reducing conditions, even if porewater CH_4_ concentrations did not increase (Figure S4C,F).

The balance between CH_4_ production and oxidation in soil also determines how much CH_4_ is emitted along the thaw gradient. Oxic, acidic, cold palsa soils emitted negligible amounts of CH_4_. In bogs, microbial activity, as well as methanogens, was reduced (AminiTabrizi et al. 2020), not only due to acidic pH and *Sphagnum*‐derived metabolites. Methanogenesis was also suppressed under higher redox potentials compared to fen, further supported by an inverse relationship between CH_4_ emissions and redox potential in the PCA (Figures 4A and 6A). Higher redox potential typically corresponds to higher availability of TEAs, enabling CH_4_ oxidation (Koskinen et al. 2020). Active methanotrophs were likely present, decreasing CH_4_ (Basiliko et al. 2004; Raghoebarsing et al. 2005). This is supported by lower porewater CH_4_ and higher porewater CO_2_ concentrations compared to fen (Figure S4A,D). In contrast, more reducing fens were susceptible to CH_4_ production, while lower redox potentials decreased CH_4_ oxidation (Kludze and DeLaune 1994) in the deeper soil layers (> 30 cm). As a result, less CH_4_ could have been consumed in fen soils, increasing the pool of CH_4_ available for emission. These processes shape the soil‐derived CH_4_ dynamics before considering the additional effects of substrate‐driven stimulation of methanogenesis and plant‐mediated CH_4_ transport.

Graminoids increased CH_4_ emissions from bogs and fens 4.4‐fold and 6.8‐fold, as they not only stimulated CH_4_ production through root‐released C but also transported CH_4_ efficiently to the atmosphere via their aerenchyma tissues (Korrensalo et al. 2021; Ström et al. 2003, 2015, 2012). The dual chamber approach used here revealed that up to 80% of CH_4_ emissions in bogs, and about 30% in fens, can be attributed to plant‐mediated transport (Figure 2C,D). The remaining CH_4_ was emitted through soil diffusion or ebullition. A previous study reported higher plant‐mediated CH_4_ transport in bogs than in fens, likely due to effective transport at low graminoid biomass (Korrensalo et al. 2021). Lower redox potentials were consistently associated with graminoid presence and enhanced CH_4_ emissions (Figure 6A), reflecting distinct geochemical conditions driven by graminoid root processes. The concurrent decrease in porewater CO_2_ (Figure S4C) with graminoid presence is consistent with a shift toward more reduced C processing, where CO_2_ reduction to CH_4_ becomes increasingly important (Laanbroek 2010). These patterns indicate that graminoid root activity shapes redox conditions, influencing CH_4_ dynamics across the thaw gradient, likely due to microbial consumption of O_2_ released from roots during accelerated electron acceptor cycling (Turner et al. 2020). In support, higher concentrations of reduced Fe in graminoid vegetated sites (Figure S6E) indicated accelerated electron acceptor cycling under O_2_‐limited conditions (Fiedler and Sommer 2004). Thus, our results support the hypothesis that plant functional types and their plant traits, through their effects on soil redox and C availability, are key controls on peatland GHG dynamics by influencing soil redox potentials. This is consistent with earlier Arctic wetland studies showing that graminoids (including *Eriophorum* ssp.) enhance CH_4_ emissions and can increase labile substrate availability (Ström and Christensen 2007; Ström et al. 2003, 2005, 2012; Varner et al. 2022). Our results extend this work by partitioning the graminoid effect into plant‐mediated transport versus soil emission pathways across bog and fen habitats and by linking these fluxes to seasonal root‐released C inputs and in situ redox/geochemical conditions.

### 4.2 Seasonal Modulation of Plant Effects on GHG Emissions

Both ecosystem‐level and plot‐scale flux measurements indicate that graminoid‐vegetated locations served as substantial CO_2_ sinks early in the growing season. When expressed as net CO_2_ equivalents they acted as sources throughout the growing season, with the largest radiative forcing occurring in mid‐to‐late growing season in both bogs and fens (Figure 3B,C). The improved alignment between plot‐scale GHG fluxes and EC tower fluxes (Figure S9) highlights ecosystem scale GHG exchange is likely best represented as an integration from the two endmembers (baseline, densely vegetated), highlighting the importance of accurately capturing the spatial distribution of vegetation when scaling plot‐scale measurements tower footprints (Davidson et al. 2017; Schrier‐Uijl et al. 2010). Because graminoids contribute disproportional to CH_4_ emissions relative to their surface area, incorporating their footprint is important to reproduce ecosystem‐scale CH_4_ fluxes (Juutinen et al. 2022; Davidson et al. 2017). This scaling sensitivity is consistent with the documented landcover change in Stordalen mire amplifying CH_4_‐driven climate forcing (Varner et al. 2022), emphasizing that graminoid distribution is critical for upscaling CH_4_ fluxes under ongoing thaw. Differences in absolute flux magnitudes between EC flux averages and plot‐scale chamber observations likely result from differences in gas sampling approaches (Bäckstrand et al. 2010).

In the PCA, CH_4_ flux was negatively correlated with the growing season months, contradicting the observed peak CH_4_ fluxes in August (Figure 6B). This discrepancy suggests that warmer soil temperatures later in the growing season enhanced CH_4_ production (Figure 3B, Table S2). More consistent with measured data was the negative correlation between redox potential and season (Figures 4B and 6B). The mismatch between CH_4_ flux and redox potential indicates the presence of additional mechanisms influencing CH_4_ emissions, such as plant‐mediated CH_4_ transport. September samples shifted toward more positive NEE, higher DOC, and higher aqueous Fe (Figures S3B and S6E; Figure S7C). This seasonal transition to September likely reflects reduced redox conditions associated with warmer temperatures and ongoing plant senescence (Li et al. 2023; Street et al. 2016; Jentzsch et al. 2024b). Shrub‐dominated palsa sites showed clearer growing seasonal separation along PC2, though explanatory power remains limited by fewer replicates and available variables (e.g., no CH_4_ emissions; Figure S11).

The observed seasonal effects may be attributed to various mechanisms. CO_2_ and CH_4_ emissions are differently affected by season, highlighting a gas‐specific effect of season (Jentzsch et al. 2024b; Li et al. 2023). CH_4_ production is more sensitive to soil temperature and soil redox conditions (Jugsujinda and Patrick Jr 1996; Rath et al. 2002). Decreasing CO_2_:CH_4_ ratios with the season thus reflect higher soil temperatures and reduced redox potentials (Figure 4B, Figure S3). This was observed in baseline soils and in graminoid‐rich fens, which exhibited an increase in CH_4_ emissions during the growing season (Figure S3G). This might indicate the importance of plant traits affecting CH_4_ emissions in the late growing season, possibly because plant senescence reduces photosynthetic O_2_ production and ROL, favoring more reducing conditions. Although plant‐mediated CH_4_ transport typically declines when photosynthesis is suppressed, passive transport through graminoid aerenchyma can persist, particularly when porewater CH_4_ accumulates (Ge et al. 2025; Jentzsch et al. 2024b). Consistently, sustained late‐season porewater CH_4_ concentrations support this mechanism, indicating sustained passive plant‐mediated transport of CH_4_ (Figure S4E). Additionally, increased and steady late‐season porewater CO_2_ and CH_4_ concentrations, combined with increased positive NEE at graminoid‐vegetated sites relative to baseline locations, suggest continued microbial stimulation through plant‐mediated C inputs (Figure 3, Figure S4). This could stem from decaying roots acting as a C source to soil microorganisms, as found before (Jentzsch et al. 2024b). Increased root‐released C from graminoids in September represents stored C in roots known for winter preparation (Heldt and Piechulla 2011) as it does not translate to significantly increased positive NEE or CH_4_ emissions compared to non‐vegetated locations (Figures 3 and 5A). Perennial palsa shrubs are also actively fixing CO_2_ in September, though not as efficiently as in August.

### 4.3 Conclusions

By analyzing a vegetation density gradient across thawing stages and seasons, our study demonstrates how vegetation controls intensify with thaw (Hough et al. 2020; Varner et al. 2022), manifest in peatlands (Järvi‐Laturi et al. 2025; Jentzsch et al. 2024b), and changes across a growing season (Järvi‐Laturi et al. 2025; Jentzsch et al. 2024b). We highlight the strong C sink potential of graminoid‐dominated peatlands, which sequester CO_2_ for most of the growing season. This effect is most pronounced with highly productive graminoids within microbially active and versatile minerotrophic fens with the highest GHG exchanges, followed by graminoids in microbially constrained bogs. In contrast, CO_2_ fixation is weakest in cold, spatially constrained palsas dominated by low‐producing shrubs. Our findings reveal that although graminoids drove strong CO_2_ fixation in both bogs and fens during June and August, CH_4_ emissions (expressed as CO_2_ equivalents) offset this uptake, resulting in net positive radiative forcing across the growing season. In September, when plants decayed, the CO_2_‐equivalent flux remained high and often increased further due to rising CO_2_ and CH_4_ emissions in graminoid‐rich areas compared to non‐graminoid sites. These findings provide critical insights into the role of plant‐specific processes, including root‐released C, microbial stimulation, and plant‐mediated GHG transport, and vegetation‐driven changes in soil redox conditions in shaping the permafrost C feedback. They also highlight the strong seasonal shift in these plant‐mediated influences. Placed in a longer‐term context, this helps explain why the ongoing transition toward wetter, graminoid‐rich fens can increase landscape‐scale CH_4_ emissions and net radiative forcing (Varner et al. 2022), even if CO_2_ uptake remains substantial during parts of the growing season. Considering the global implications of northern peatland GHG emissions (Christensen 2024) and the importance of shoulder‐season fluxes (Yuan et al. 2024), extending similar set‐ups to capture year‐round dynamics of these plant controls is essential. Further investigations into how vegetation‐driven soil conditions vary beyond the growing season could substantially advance our understanding of peatland responses to ongoing permafrost thaw.

## Author Contributions

Funding for this work was acquired by M.M., E.M.M., and A.K. This work was conceptualized by E.M.M. and M.M. M.M., E.M.M., and A.K. designed the project, interpreted the data, and wrote the manuscript. M.M., E.M.M., K.L., and S.D. collected the samples and gathered the data. S.M., E.D., and B.W. advised on fieldwork and data interpretation. P.J. and S.G. helped with fruitful discussions and fieldwork. All authors contributed to the preparation of the manuscript.

## Funding

This study was supported by the Deutsche Forschungsgemeinschaft (DFG, German Research Foundation) and proposal number 465839667. E.M.M. is supported by the Helmholtz Young Investigator Project RhizoThreats. B.W. additionally acknowledges ERC StG PRIMETIME (grant number 101039588) and the Swedish Research Council VR (grant number 2022‐03940).

## Conflicts of Interest

The authors declare no conflicts of interest.

## Supporting information


**Data S1:** gcb70783‐sup‐0001‐Supinfo.docx.

## Data Availability

The datasets generated during and/or analyzed during the current study are also available on zenodo (https://doi.org/10.5281/zenodo.18363380).
