# Supplementary material for: Graminoids Increase Greenhouse Gas Emissions From Thawed Permafrost at the End of the Growing Season
Source: Glob Chang Biol. 2026 Mar 9;32(3):e70783. doi: 10.1111/gcb.70783 (PMC12969541; doi:10.1111/gcb.70783)
Supplement: Supplementary file 1 — Data S1: gcb70783‐sup‐0001‐Supinfo.docx. [file GCB-32-e70783-s001.docx]

**Graminoids increase greenhouse gas emissions from thawed permafrost at the end of the growing season**

Marie Mollenkopf^1,2,3^, Katja Lenge^2^, Sören Drabesch^1,2^, Sylvain Monteux^4,5^, Sigrid van Grinsven^1^, Prachi Joshi^1,6^, Ellen Dorrepaal^7^, Birgit Wild^4,8^, Andreas Kappler^1,9^, E. Marie Muehe^2,3^

*^1^Geomicrobiology, Department of Geosciences, University of Tübingen, Tübingen, Germany*

*^2^Plant Biogeochemistry, Helmholtz Centre for Environmental Research -UFZ, Leipzig, Germany*

*^3^Plant Biogeochemistry, Department of Geosciences, University of Tübingen, Tübingen, Germany*

*^4^Department of Environmental Science, Stockholm University, Stockholm, Sweden*

*^5^Tromsø Museum, UiT The Arctic University of Norway, Tromsø, Norway"*

*^6^Forest Soils and Biogeochemistry, Swiss Federal Institute for Forest, Snow and Landscape Research, Birmendorf, Switzerland*

*^7^Department of Ecology and Environmental Science, Climate Impacts Research Centre, University of Umeå, Abisko, Sweden*

*^8^Bolin Centre for Climate Research, Stockholm University, Stockholm, Sweden*

*^9^Cluster of Excellence: EXC 2124: Controlling Microbes to Fight Infection, Tübingen, Germany*

**Supplements**

**Methods S1 | Quantification of organic carbon metabolites by gas chromatography coupled to mass spectrometry (GC-MS).**

Organic carbon metabolites were quantified using GC-MS (Shimadzu GC/MS TQ 8040, Japan) in Electron Ionization mode (EI). Metabolites were quantified either by headspace injection (MS acquisition mode: Scan) or as a liquid sample after derivatization (MS acquisition mode: MRM).

For derivatization, samples were thawed on ice and 3000 pmol ^13^C-glucose was added as an internal standard to 250 µL sample prior to freeze-drying. Re-dissolution of solids was done with 50 µL methoxamine (20 mg mL^-1^ pyridine), ultrasonicated for 10 min and then incubated at 30°C for 90 min. Subsequently, 70 µL N-methyl-N-(trimethylsilyl)-trifluor-acetamid (MSTFA) was added and samples were incubated at 40°C for 60 min and thereafter kept at room temperature for 2 h (adjusted to Fiehn et al. 2016). A volume of 1 µL was used for injection in split mode on a Restek SH-Rxi-5SIL MS column (30 m; film 0.25 µm; diameter 0.25 mm). The injection port was heated to 280°C. For chromatographic separation, a helium flow rate of 1.1 mL min^-1^ and a temperature was increased by 10°C min^-1^ from 100 to 320°C.

For the headspace measurement (Fiorini, Boarelli, Gabbianelli, Ballini, & Pacetti, 2016; Zhang et al., 2018), 250 µL of thawed sample together with 2500 pmol octanol as an internal standard were added into glass vials with closed lids. A volume of 1 ml of headspace gas was injected onto the column Stabilwax-DA from Restek. The helium flow rate was 1.1 mL min^-1^ and the column temperature increased by 10°C min^-1^ from 40 to 240°C. Concentrations were quantified with an 8-point external calibration using standards of all targeted analytes, ranging from 20 to max. 10^7^ pmol per 250 µL. Given metabolite-specific losses during sample preparation and derivatization extents, metabolite concentrations should be considered as semi-quantitative.

**Fig. S1 ǀ Sampling locations and meteorological data at the field site Stordalen Mire, Abisko, Sweden.** (A) Soil and plant samples were taken from palsa (brown), bog (green), and fen (blue) locations. (B, C) Meteorological data of the sampling season in 2023 (black) and the average of 2016-2023 (red). Sampling campaigns are indicated by bars: June/July in yellow, August in orange, red in September. Data provided by the Integrated Carbon Observatory System (ICOS) portal (ICOS Sweden, 2021a, 2021b, 2021c, 2021d, 2022, 2023, Lundin, et al., 2025).


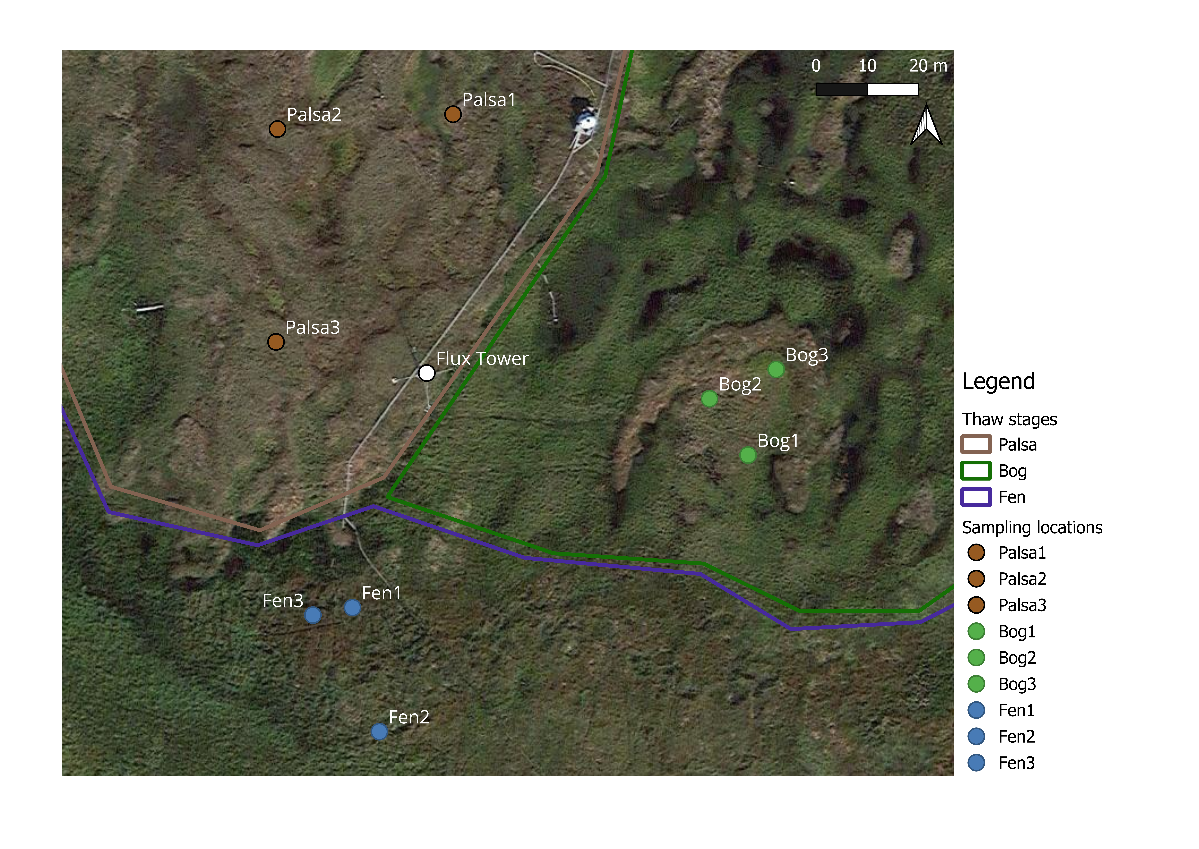

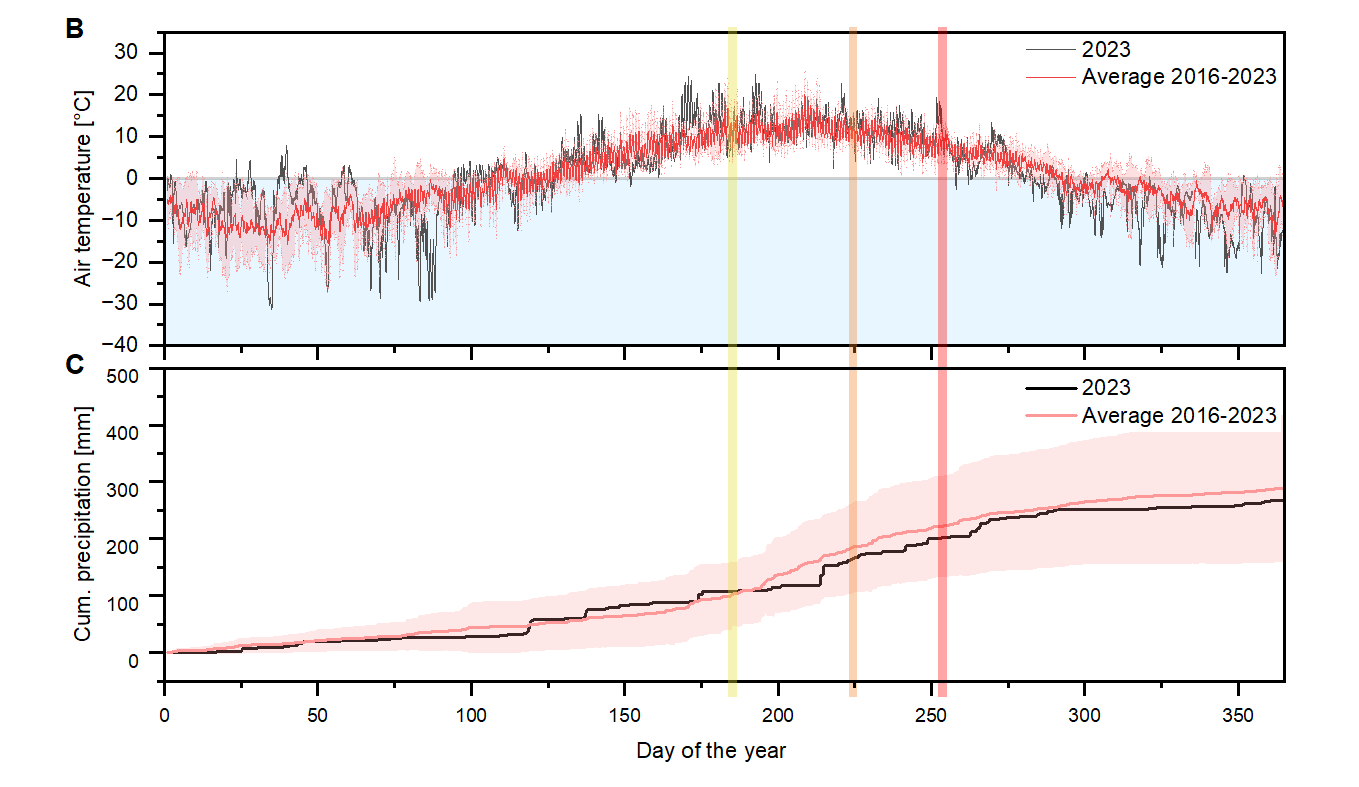

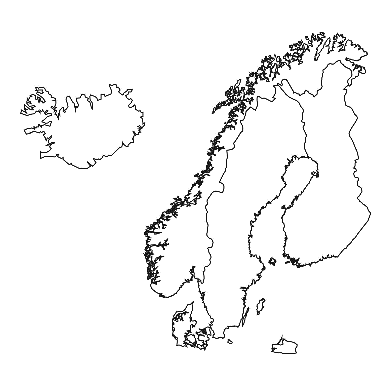


**A**


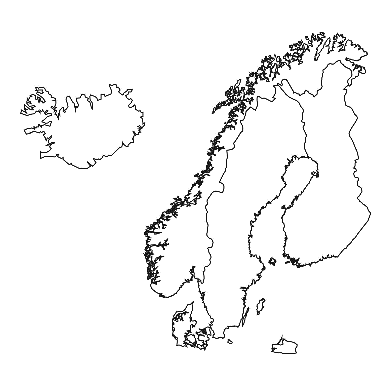
**Fig. S3 ǀ Greenhouse gas emissions across increasing degree of vegetation along permafrost thaw gradients at three seasonal timepoints.** Net ecosystem exchange (NEE; net CO_2_ flux) from (A) Palsa, (B) Bog, and (C) Fen and CH_4_ fluxes from (D) Bog and (E) Fen in baseline (non-vascular plant reference; empty boxes), sparsely vegetated (striped boxes), and densely vegetated (filled boxes) locations. Baseline plots were dominated by non-vascular vegetation (mosses/lichens) or waterlogged soil. Boxes indicate interquartile range (25th–75th percentiles)*,* whiskers denote ±1.5× IQR. (F) Bog and (G) Fen CO_2_:CH_4_ ratios within the season were calculated from dark chamber CO_2_ and light chamber CH_4_ (Bog CO_2_:CH_4_ ratio for June missing due to missing bog dark chamber data in June). Mean ± 1SD. N(palsa)=3-5, N(bog)= 3-9, N(fen)=6-8.


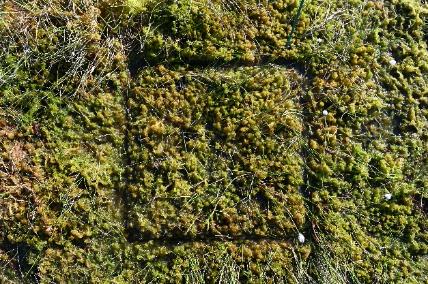

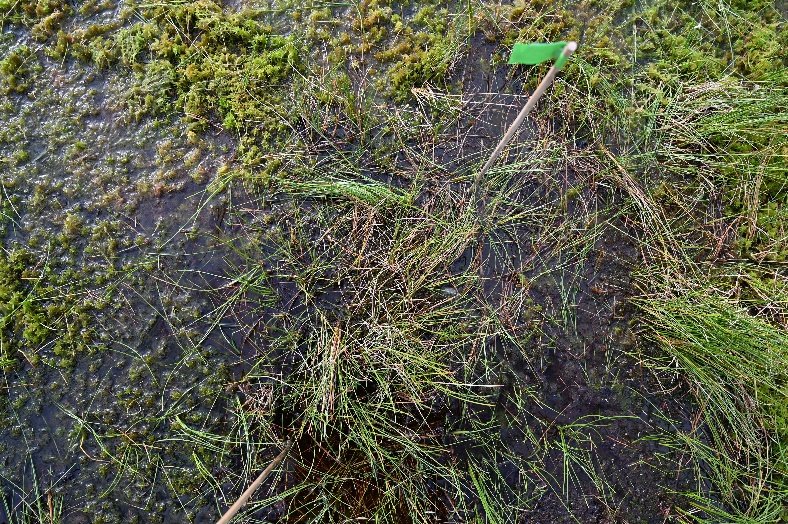

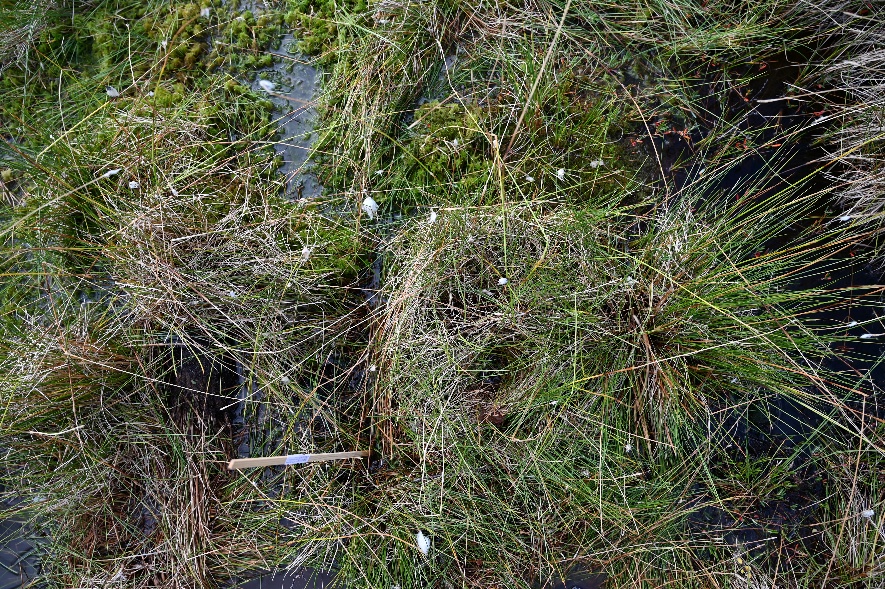

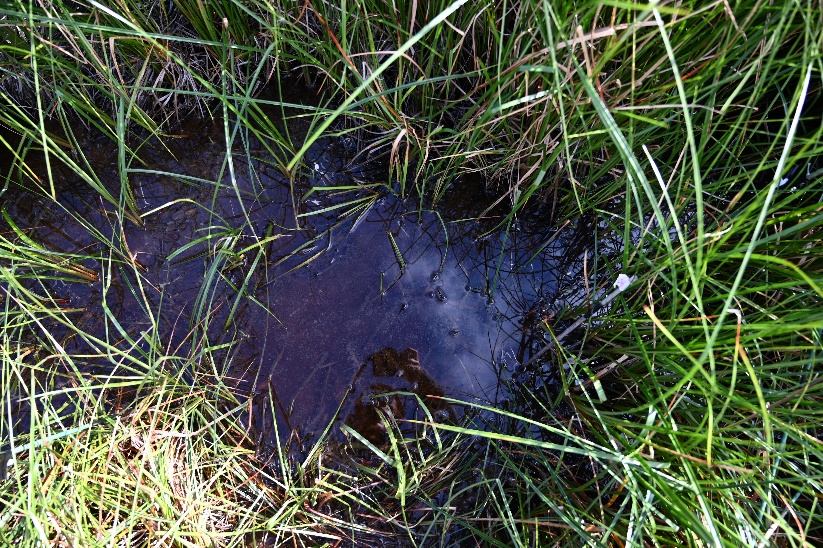

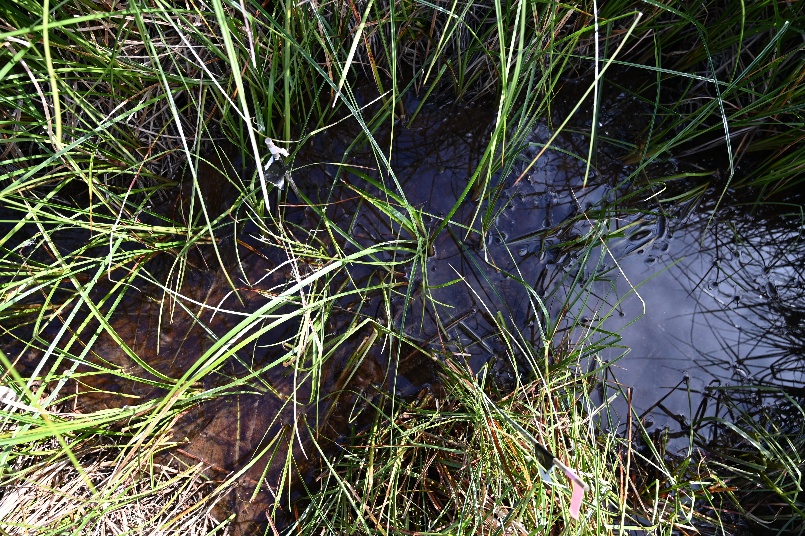

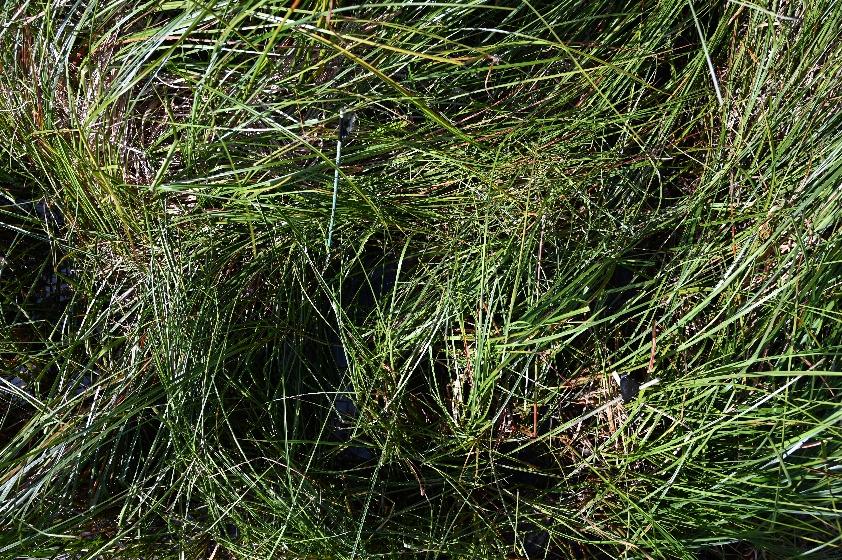

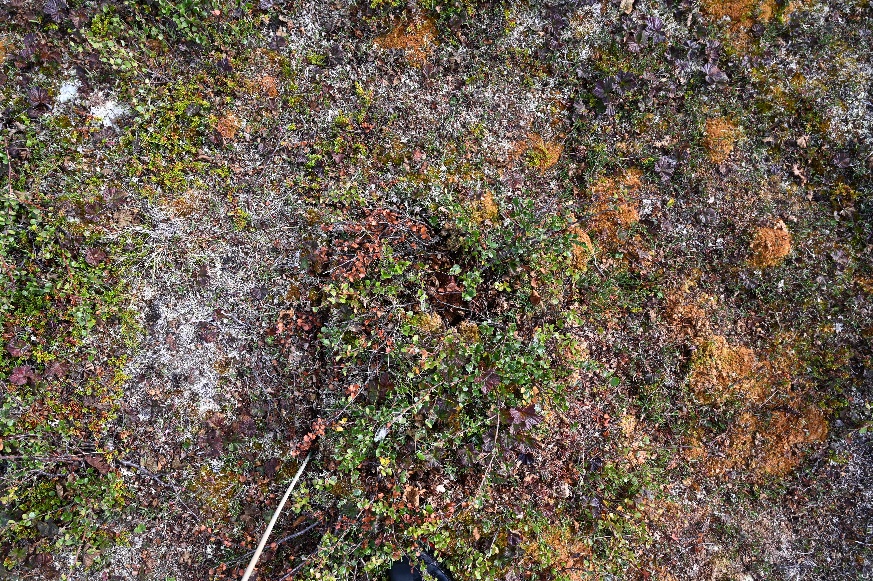

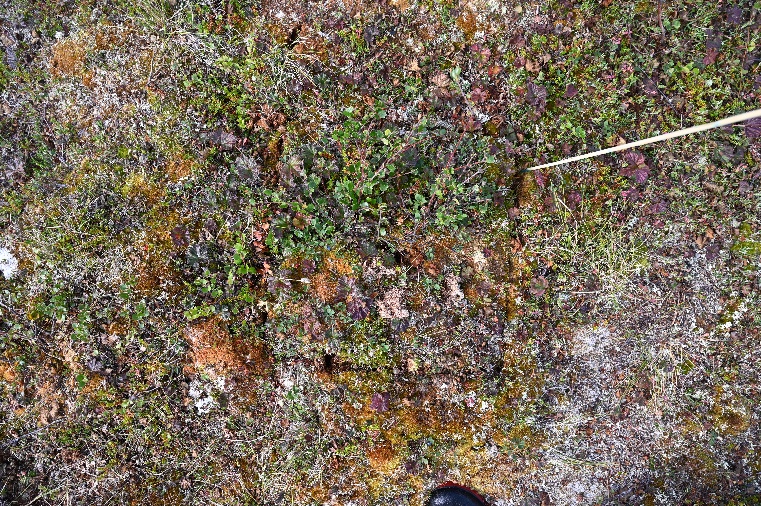

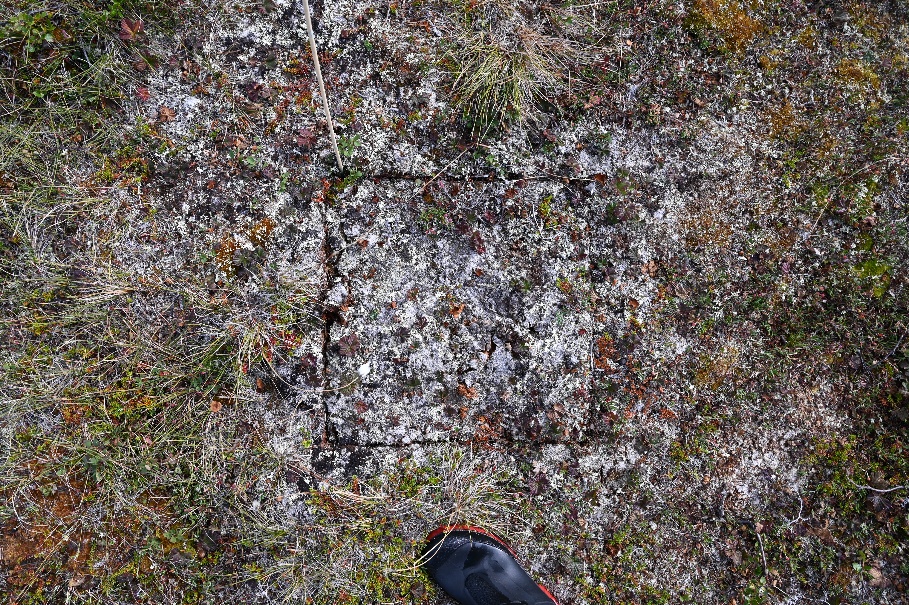


**A**

**B**

**C**

**D**

**E**

**F**

**G**

**H**

**I**

**Fig. S2 ǀ Representative sampling locations along the thaw gradient.** Palsa (A) baseline (no shrubs, but lichen/mosses), (B) sparsely shrub vegetated, and (C) densely shrub vegetated. Bog (D) baseline (Sphagnum ssp.)**,** (E) sparsely graminoid vegetated, and (F) densely graminoid vegetated. Fen (G) baseline (waterlogged soil), (H) sparsely graminoid vegetated, and (I) densely graminoid vegetated locations.


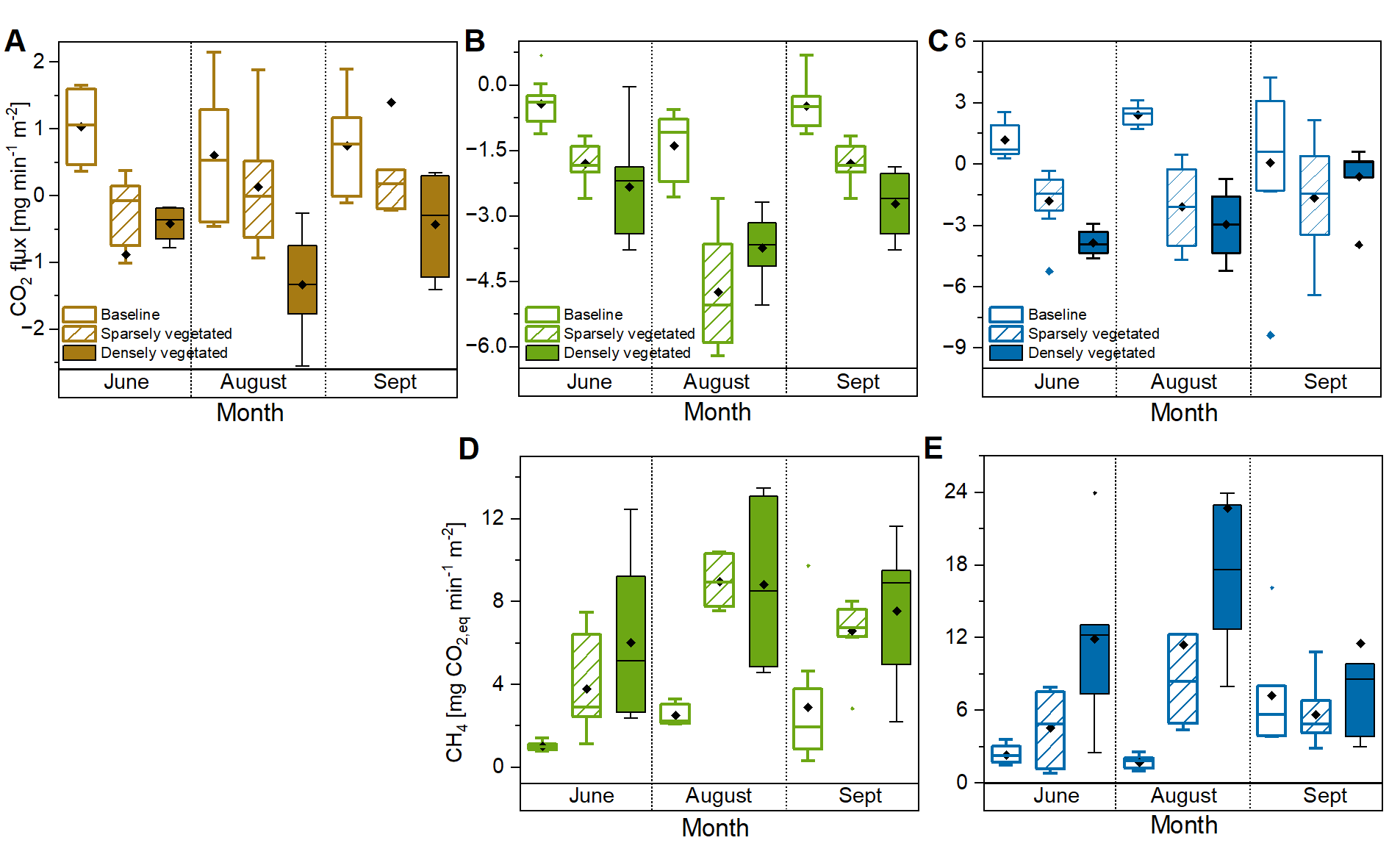

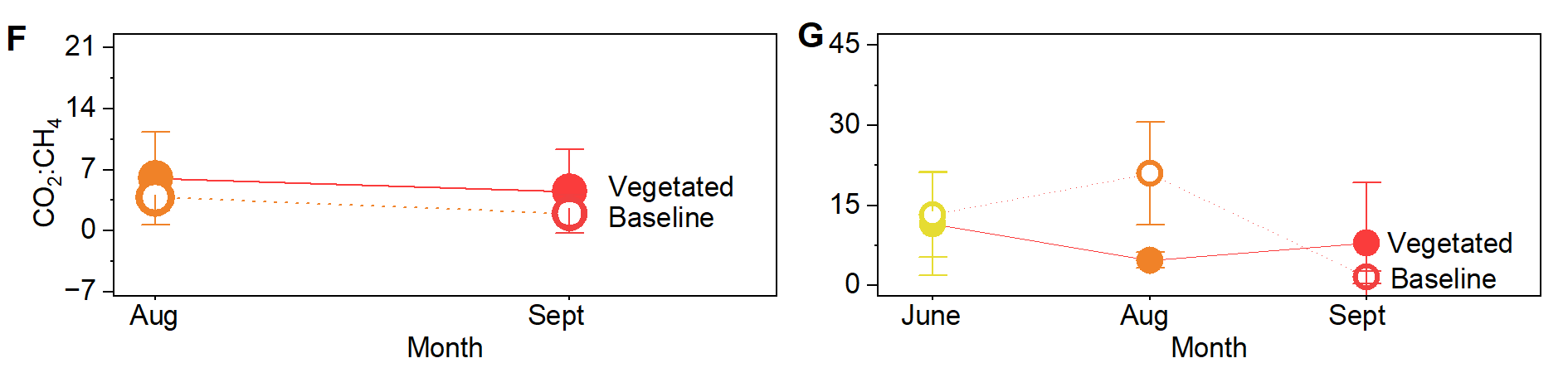


**Fig. S4 ǀ Porewater dissolved gases resolved to soil depth, growing season and vegetation density.** Porewater CO_2_ (A) in a depth profile per thaw stage, (B) across a growing season, (C) depending on vegetation density. Porewater CH_4_ (D) in a depth profile per thaw stage, (E) across a growing season, (F) depending on vegetation density. Circles in A and D represent the mean, shaded areas represent the 1SE and is interpolated with depths, N=8-10. Black diamonds in B, C, E, F represent the mean, the central box line the median, boxes indicate interquartile range (25th–75th percentiles), whiskers denote ±1.5× IQR. Boxplots show data from porewater gas extracted at 30 cm depth at the bog site.


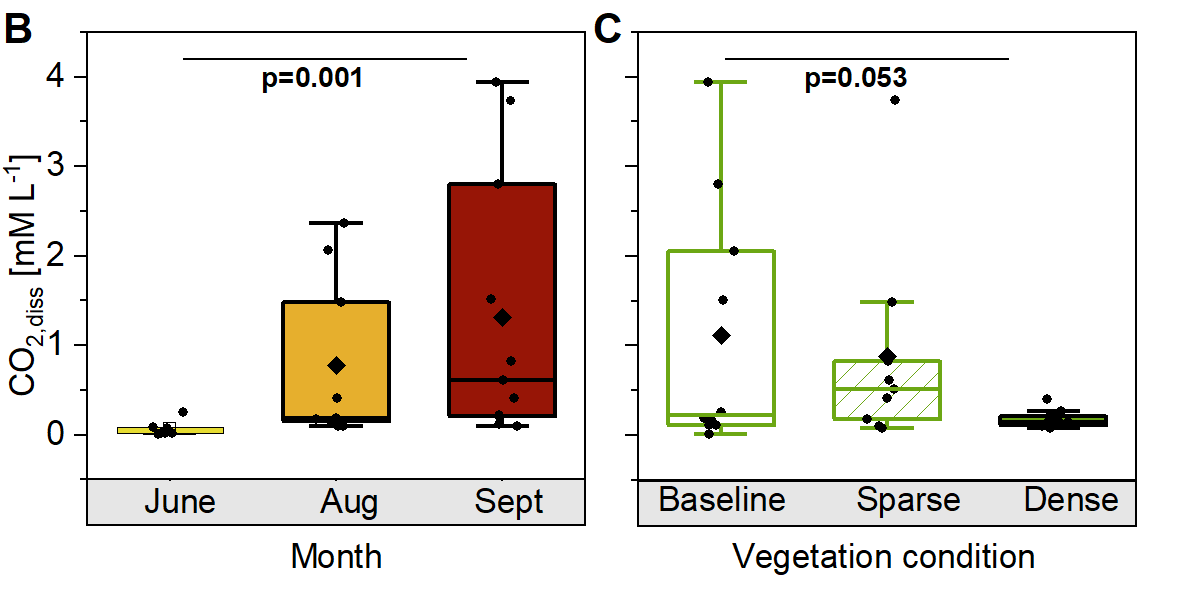

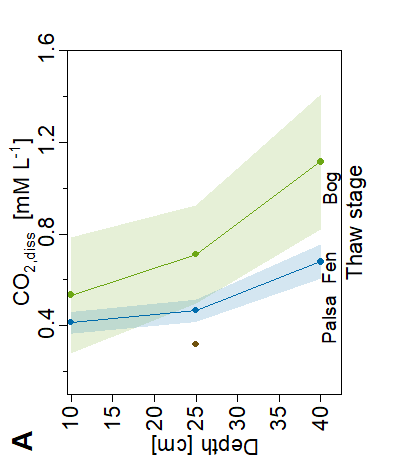

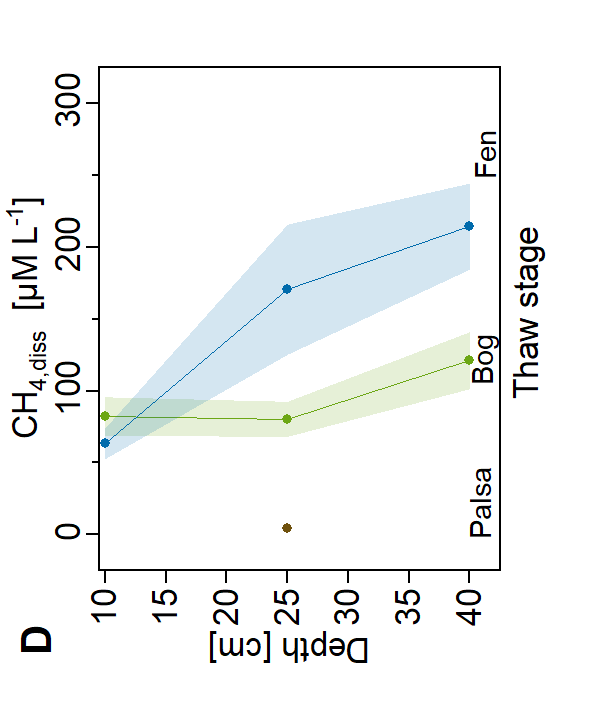

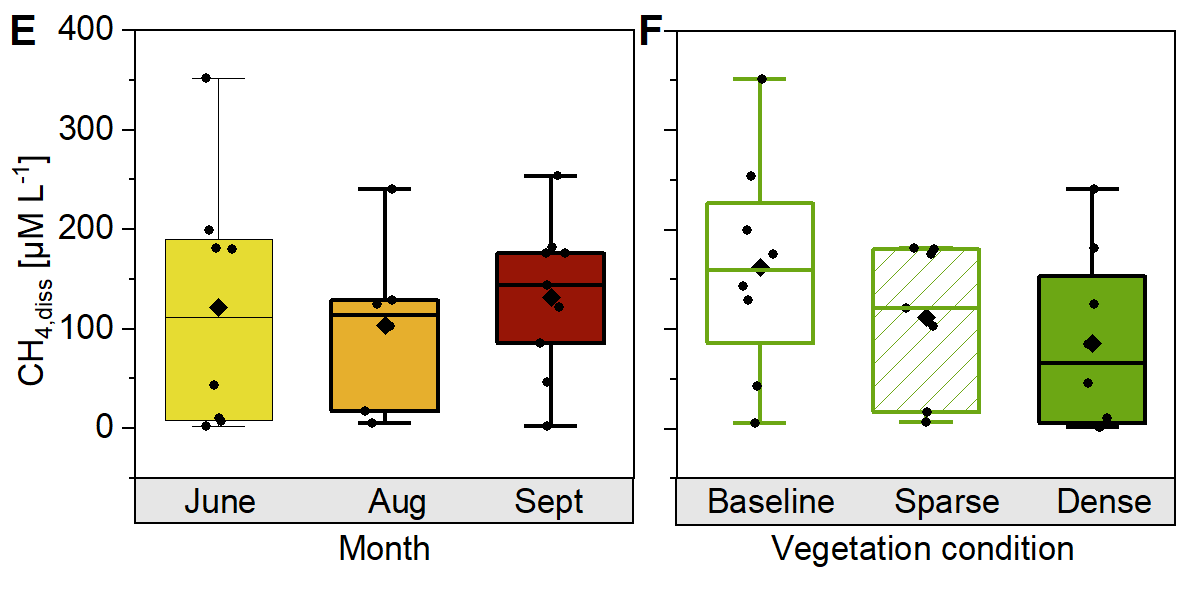


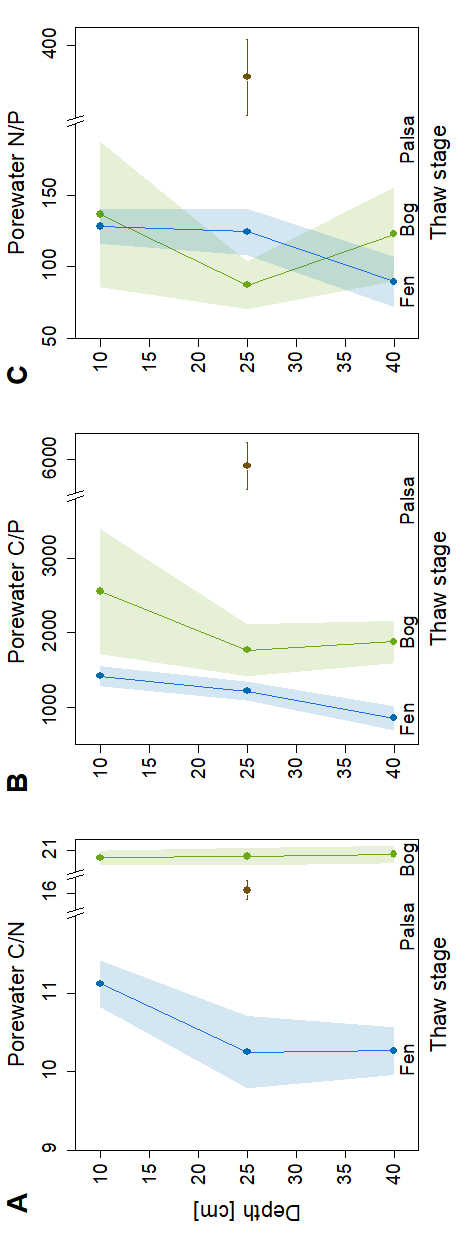
**Fig. S5 ǀ Porewater nutrient stoichiometry as affected by progressive thaw and soil depth.** (A) Porewater C/N, (B) C/P, and (C) N/P with depth. Circles represent the mean; the shaded area represents 1SD and is interpolated with depths.

**Fig. S6 ǀ Porewater iron as affected by soil depth, degree of vegetation, and growing season.** Porewater (A) Fe_tot_, (B) Fe(II), circles represent the mean, the shaded area represents 1SD and is interpolated with depths. N=3-9. (C) Fe_tot_ and (D) Fe(II) with increasing graminoid density in the bog and (E) Fe_tot_ within a growing season. Boxes indicate interquartile range (25th–75th percentiles), whiskers denote ±1.5× IQR. Statistical significance was assessed using a two-sided t-test (p<0.05). Boxplots show data from porewater gas extracted at 30 cm depth at the bog site.


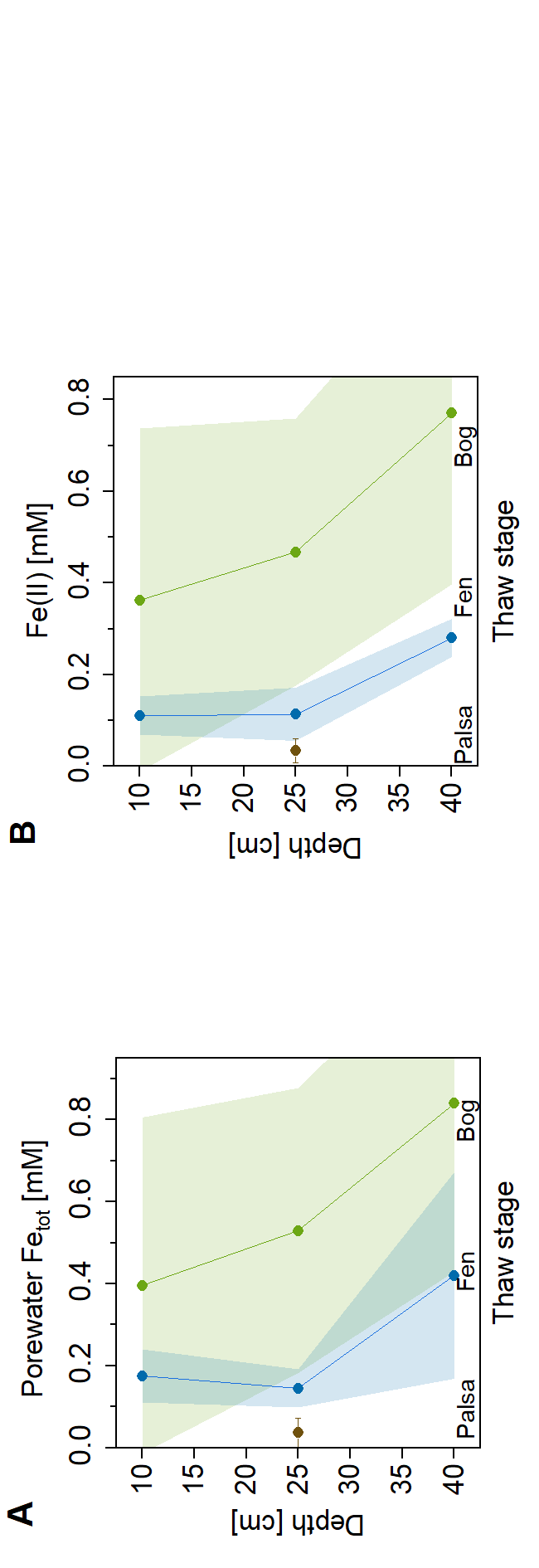

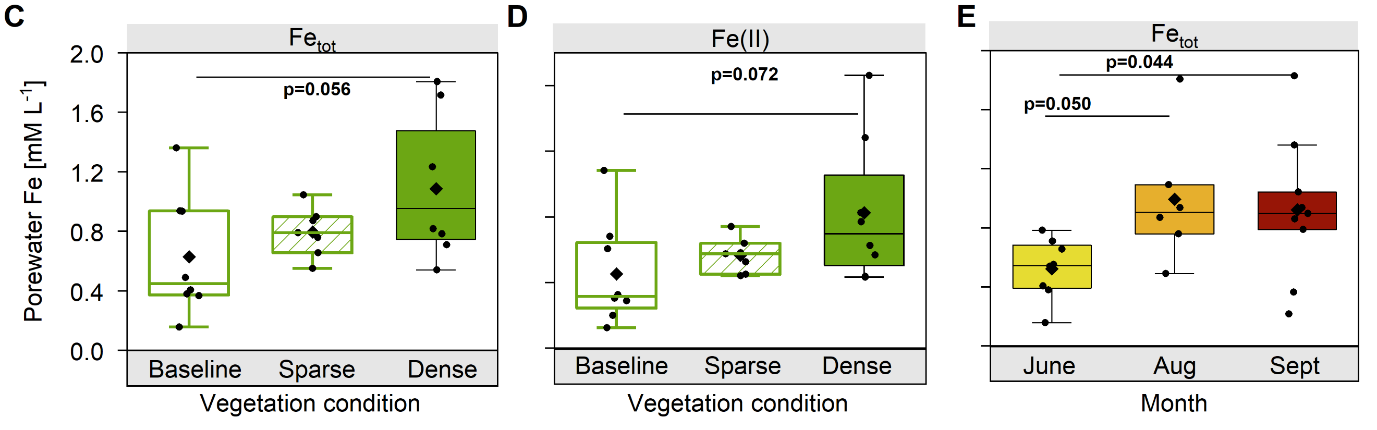

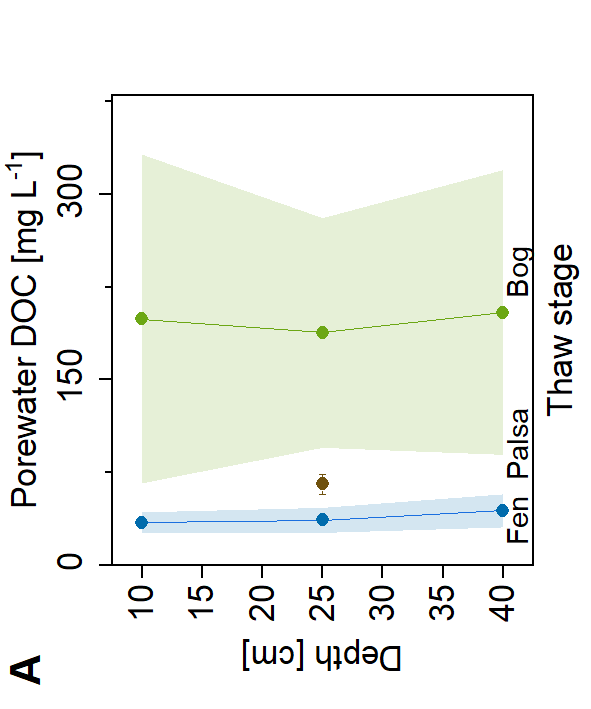

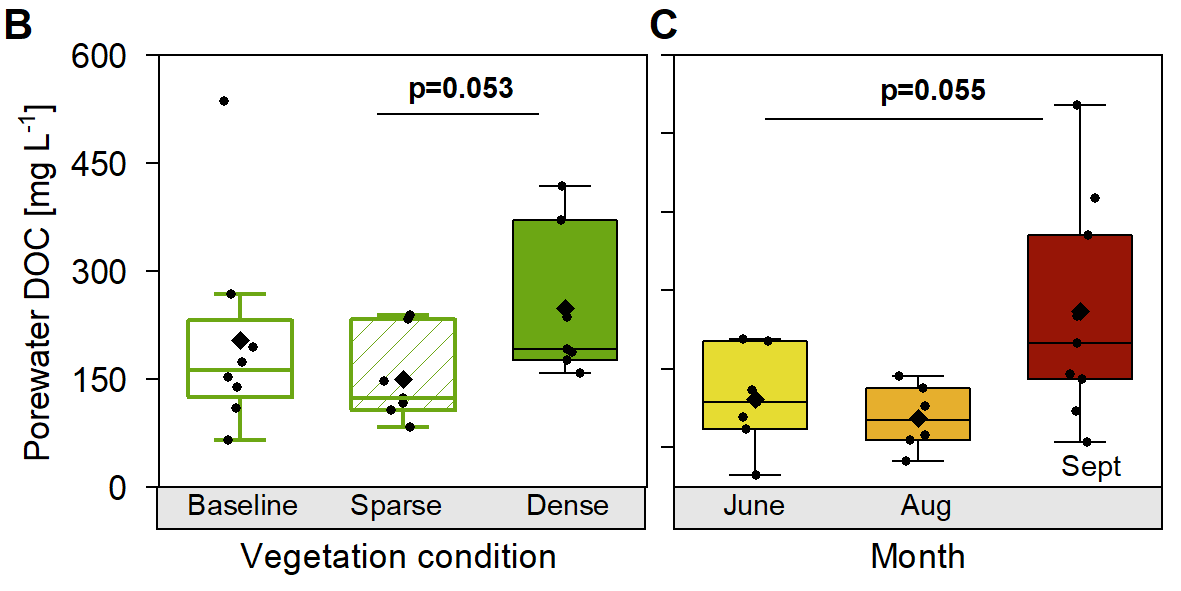


**Fig. S7 ǀ Porewater DOC as affected by progressive thaw, degree of vegetation, and growing season.** (A) DOC with depths and different thaw stages. Circles represent the mean; the shaded area represents 1SD and is interpolated with depths. N=3-8. (B) DOC with increasing graminoid density in the bog and (C) within a growing season. Boxes indicate interquartile range (25th–75th percentiles), whiskers denote ±1.5× IQR. Statistical significance is assessed with a two-sided t-test (p<0.05). Boxplots show data from porewater gas extracted at 30 cm depth at the bog site.


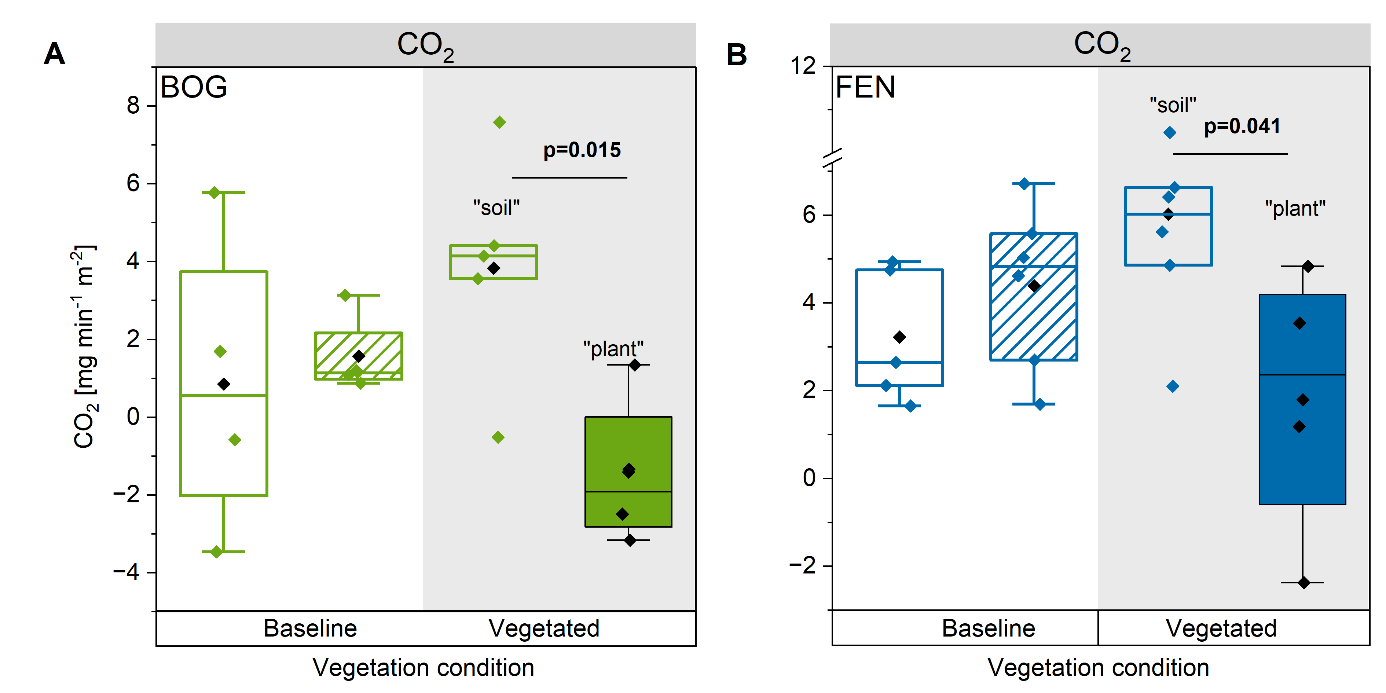
**Fig. S8 ǀ CO_2_ emissions from baseline (non-vascular plant reference) and vegetated permafrost thaw stages, distinguishing between direct soil diffused and plant-mediated transport fluxes.** Baseline plots were dominated by non-vascular vegetation (mosses/lichens) or waterlogged soil. A dual-chamber approach was used to separate emissions directly from the soil (“soil”, depicted as empty boxplots) and those transported through the plant (“plant”, depicted as filled boxplots), with gas samples collected from a chamber and a gas-tight bag around the plant (or non-vascular plant spots as control, the dual-chamber control depicted as empty and striped boxplots). Measurements were taken from (A) bog (green) and (B) fen (blue) environments in both baseline and vegetated locations. Boxes indicate interquartile range (25th–75th percentiles), whiskers denote ±1.5× IQR. N=4-6. Statistical significance was assessed using a two-sided t-test (p≤0.05).


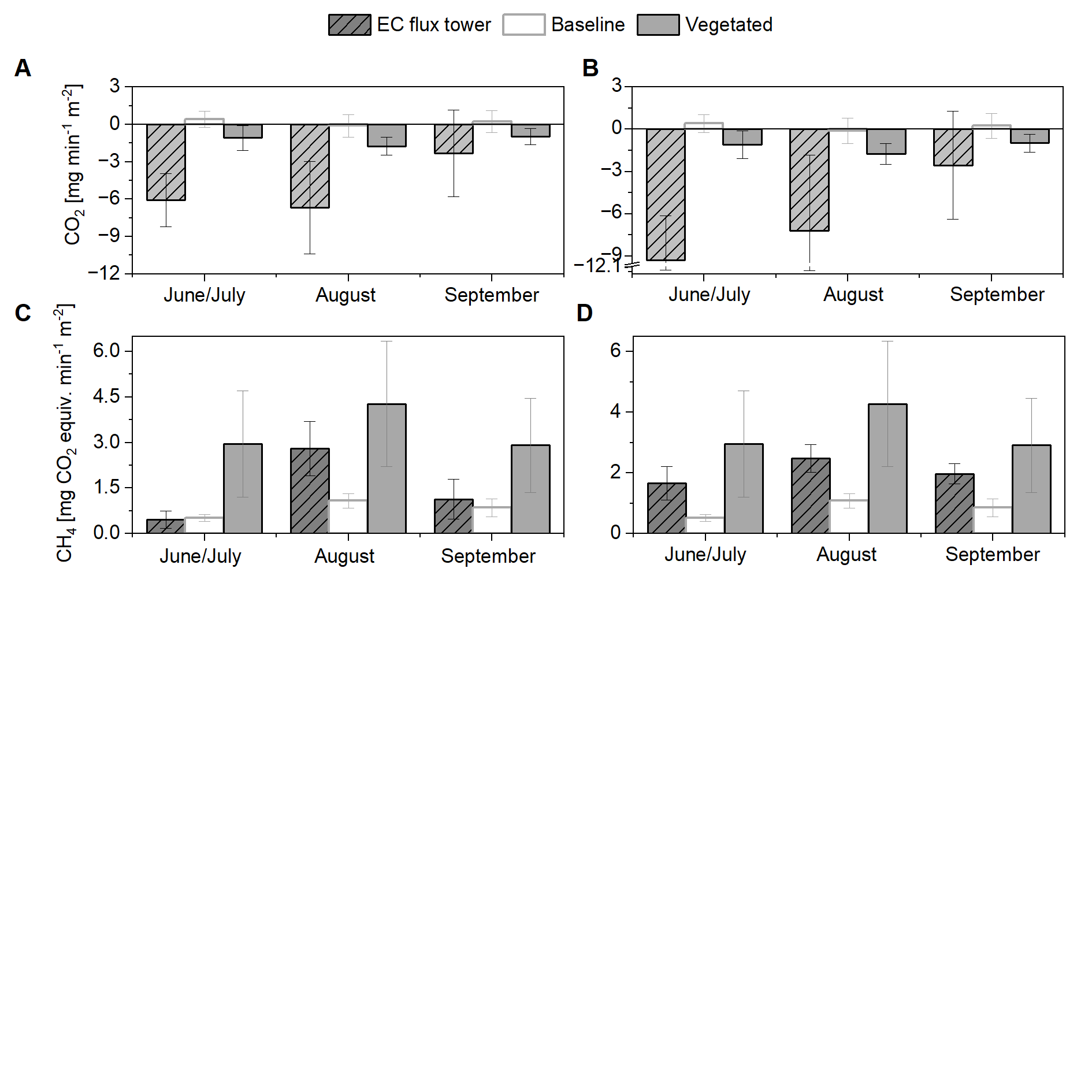
**Fig. S9 ǀ Eddy Covariance (EC) flux tower footprint approach comparing the ecosystem-level net ecosystem exchange (NEE) and CH_4_ fluxes to plot-scale chamber fluxes.** Plot-scale flux data were weighed based on the footprint habitat areas of the flux tower and then compared to EC NEE and CH_4_ fluxes (EC data obtained from the Integrated Carbon Observatory System, Sweden (ICOS Sweden, 2019, 2021, 2022, 2023; Lundin et al., 2025) during the time of sampling campaigns. (A, B) NEE and (C, D) CH_4_ flux comparison for the palsa-dominating scenario. (A, C) plot-scale fluxes compared to averaged EC fluxes across 4 years, (B, D) compared to EC fluxes of 2023, only accounting for the day (8 am to 8 pm). Footprint data from Laasonen et al. (2025)^.^ Mean ± 1SD.

*
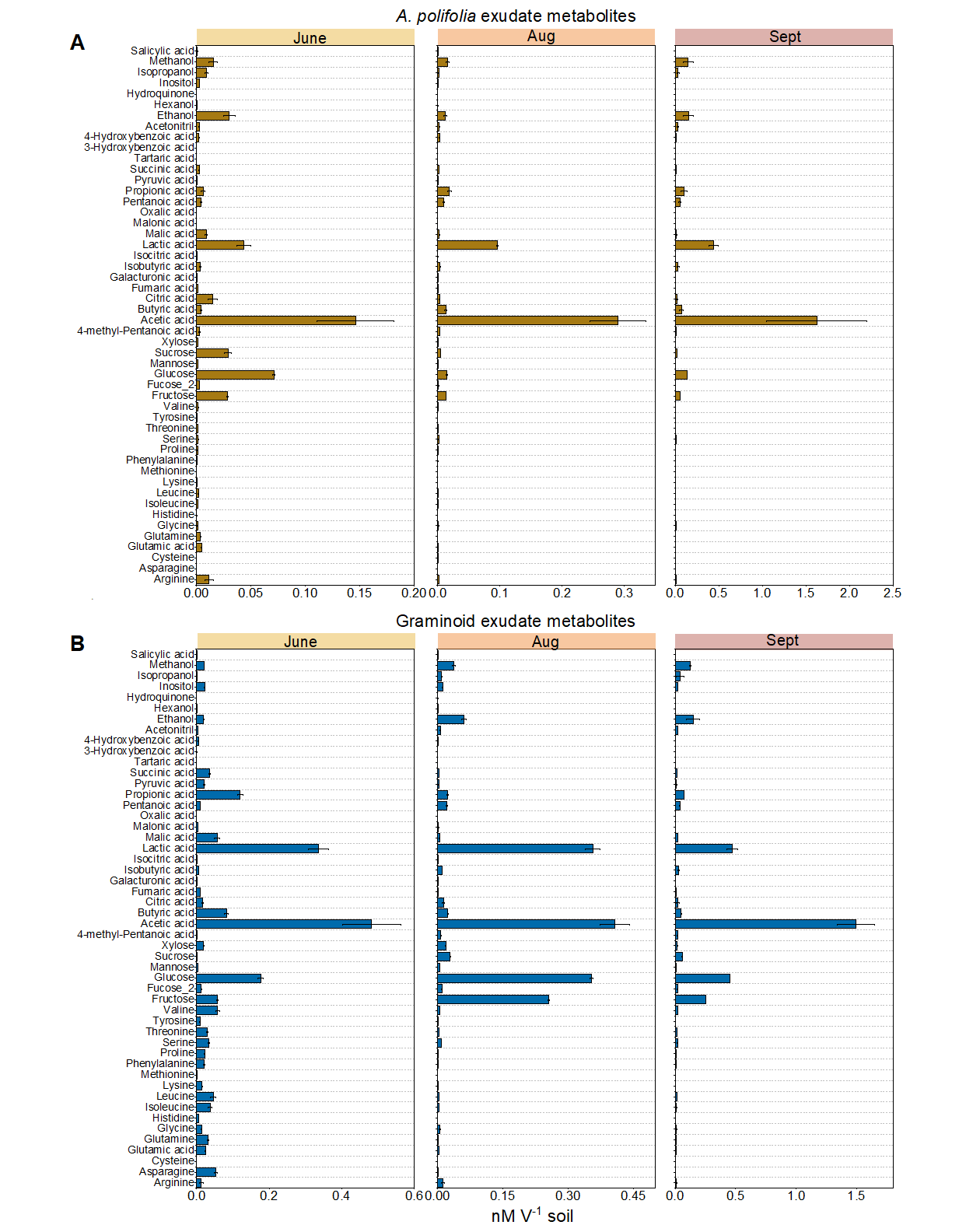
*

**Fig. S10 ǀ Root-released metabolites identified through targeted GCxMS analysis.** Targeted metabolites commonly found in root exudation and soils (AminiTabrizi et al., 2020, Williams et al., 2021, Tab. S2) were analyzed in (A) A. polifolia exudates (brown) collected from palsa, and (B) graminoid exudates (blue), collected from thawed soil (bog and fen). Samples were collected in June (yellow), August (orange), and September (red). Mean ± 1SD.


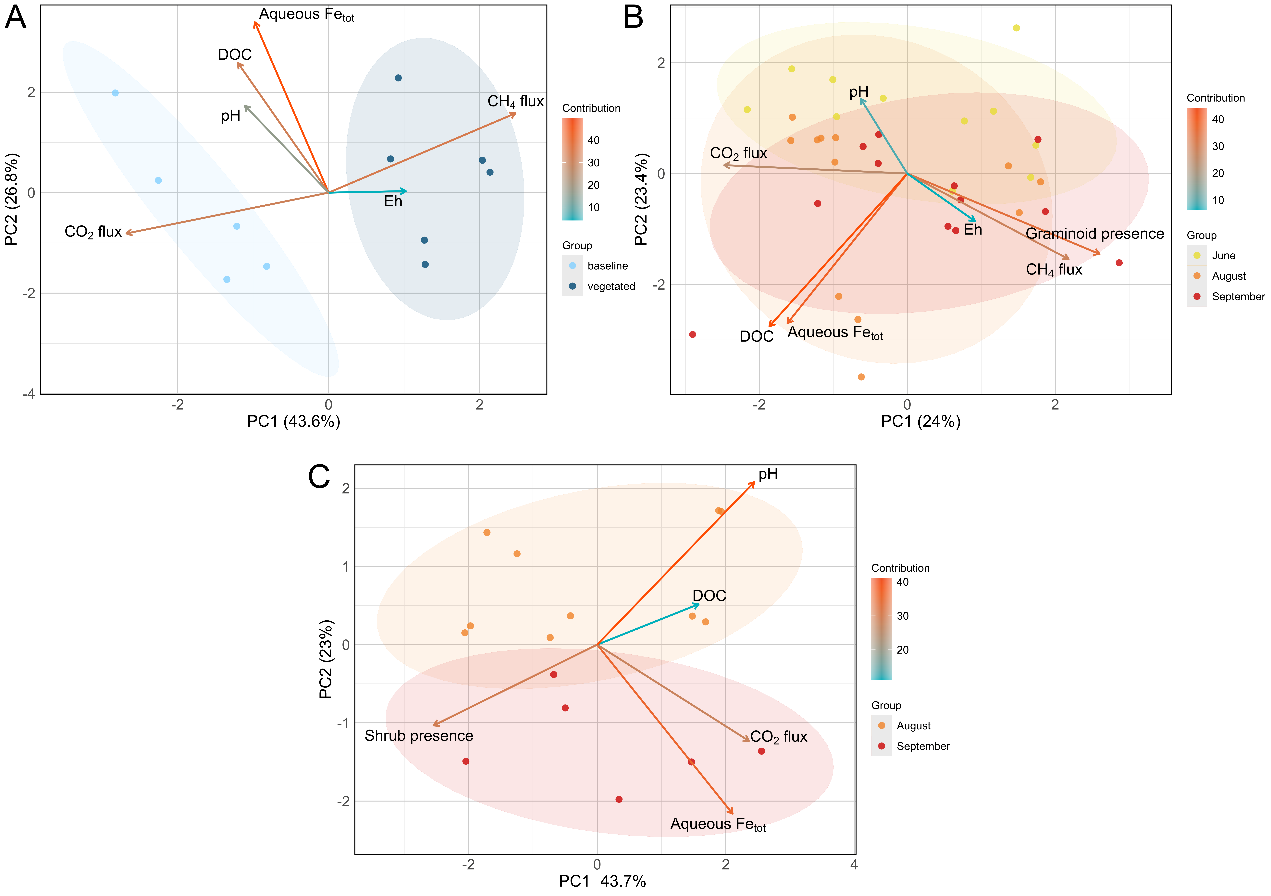
**Fig. S11 ǀ Principal component analyses (PCA) illustrating how soil geochemistry, greenhouse gas fluxes, and vegetation characteristics respond to growing season timepoints and vegetation densities.** (A) Separation of fen sites based on graminoid presence (baseline (non-vascular plant reference, shrub/graminoid vegetated) highlights vegetation effects on geochemical properties, (B) fen sites and (C) palsa sites resolved for sampling season (June, August, September). Baseline plots were dominated by non-vascular vegetation (mosses/lichens) or waterlogged soil. Ellipses show 80% confidence intervals around clusters. Loadings indicate the direction and contribution (color-coded in percentage) of key environmental variables.

**Tab. S1 | Overview of environmental and spatial characteristics of the sampling points.** The table includes information on growing season timepoint, thaw stage, weather conditions, location, active layer depth, water table depth, plant density, sampling date, and geographic coordinates (latitude and longitude) for each sampling point or vegetation density profile (e.g., active layer depth).

| **Season** | **Thaw stage** | **Weather** | **Location** | **Active layer depth [cm]** | **Water table depth [cm]** | **Plant density [%]** | **Sampling date** | **Latitude** | **Longitude** |
| --- | --- | --- | --- | --- | --- | --- | --- | --- | --- |
| June/July | Bog | sunny | Bog1 | 43 | 9 | >70 | 27.06.2023 | 68°21'18"N | 19°02'44"E |
| June/July | Bog | sunny | Bog1 | 43 | 11 | 20-60 | 27.06.2023 | 68°21'18"N | 19°02'44"E |
| June/July | Bog | sunny | Bog1 | 43 | 7 | <10 | 27.06.2023 | 68°21'18"N | 19°02'44"E |
| June/July | Bog | sunny | Bog2 | 47 | 2 | >70 | 28.06.2023 | 68°21'19"N | 19°02'44"E |
| June/July | Bog | sunny | Bog2 | 47 | 9 | 20-60 | 28.06.2023 | 68°21'19"N | 19°02'44"E |
| June/July | Bog | sunny | Bog2 | 47 | 3 | <10 | 28.06.2023 | 68°21'19"N | 19°02'44"E |
| June/July | Bog | sunny | Bog3 | 44 | 3,25 | >70 | 29.06.2023 | 68°21'19"N | 19°02'45"E |
| June/July | Bog | sunny | Bog3 | 44 | 6 | 20-60 | 29.06.2023 | 68°21'19"N | 19°02'45"E |
| June/July | Bog | sunny | Bog3 | 44 | 0 | <10 | 29.06.2023 | 68°21'19"N | 19°02'45"E |
| August | Bog | cloudy | Bog1 | >60 | 3 | >70 | 13.08.2023 | 68°21'18"N | 19°02'44"E |
| August | Bog | cloudy | Bog1 | >60 | 3,5 | 20-60 | 13.08.2023 | 68°21'18"N | 19°02'44"E |
| August | Bog | cloudy | Bog1 | >60 | 3 | <10 | 13.08.2023 | 68°21'18"N | 19°02'44"E |
| August | Bog | cloudy | Bog2 | >60 | 4 | >70 | 14.08.2023 | 68°21'19"N | 19°02'44"E |
| August | Bog | cloudy | Bog2 | >60 | 5 | 20-60 | 14.08.2023 | 68°21'19"N | 19°02'44"E |
| August | Bog | cloudy | Bog2 | >60 | 3 | <10 | 14.08.2023 | 68°21'19"N | 19°02'44"E |
| September | Bog | cloudy | Bog1 | >60 | 5 | >70 | 06.09.2023 | 68°21'18"N | 19°02'44"E |
| September | Bog | cloudy | Bog1 | >60 | 5 | 20-60 | 06.09.2023 | 68°21'18"N | 19°02'44"E |
| September | Bog | cloudy | Bog1 | >60 | 7 | <10 | 06.09.2023 | 68°21'18"N | 19°02'44"E |
| September | Bog | cloudy | Bog2 | >60 | 1 | >70 | 06.09.2023 | 68°21'19"N | 19°02'44"E |
| September | Bog | cloudy | Bog2 | >60 | 7 | 20-60 | 06.09.2023 | 68°21'19"N | 19°02'44"E |
| September | Bog | cloudy | Bog2 | >60 | 1 | <10 | 06.09.2023 | 68°21'19"N | 19°02'44"E |
| September | Bog | cloudy | Bog3 | >60 | 1 | >70 | 08.09.2023 | 68°21'19"N | 19°02'45"E |
| September | Bog | cloudy | Bog3 | >60 | 3 | 20-60 | 08.09.2023 | 68°21'19"N | 19°02'45"E |
| September | Bog | cloudy | Bog3 | >60 | 0 | <10 | 08.09.2023 | 68°21'19"N | 19°02'45"E |
| June/July | Fen | sunny | Fen2 | >60 | 12 | >70 | 30.06.2023 | 68°21'17"N | 19°02'38"E |
| June/July | Fen | sunny | Fen2 | >60 | 14 | 20-60 | 30.06.2023 | 68°21'17"N | 19°02'38"E |
| June/July | Fen | sunny | Fen2 | >60 | 21 | <10 | 30.06.2023 | 68°21'17"N | 19°02'38"E |
| June/July | Fen | sunny | Fen1 | >60 | 14,5 | >70 | 30.06.2023 | 68°21'17"N | 19°02'38"E |
| June/July | Fen | sunny | Fen1 | >60 | 24 | 20-60 | 30.06.2023 | 68°21'17"N | 19°02'38"E |
| June/July | Fen | sunny | Fen1 | >60 | 23 | <10 | 30.06.2023 | 68°21'17"N | 19°02'38"E |
| August | Fen | sunny-cloudy | Fen1 | >60 | 12 | >70 | 11.08.2023 | 68°21'17"N | 19°02'38"E |
| August | Fen | sunny-cloudy | Fen1 | >60 | 17 | <10 | 11.08.2023 | 68°21'17"N | 19°02'38"E |
| August | Fen | sunny-cloudy | Fen1 | >60 | 9 | 20-60 | 11.08.2023 | 68°21'17"N | 19°02'38"E |
| August | Fen | cloudy | Fen2 | >60 | 16 | >70 | 12.08.2023 | 68°21'17"N | 19°02'38"E |
| August | Fen | cloudy | Fen2 | >60 | 14 | 20-60 | 12.08.2023 | 68°21'17"N | 19°02'38"E |
| August | Fen | cloudy | Fen2 | >60 | 20 | <10 | 12.08.2023 | 68°21'17"N | 19°02'38"E |
| September | Fen | cloudy | Fen2 | >60 | 12 | >70 | 05.09.2023 | 68°21'17"N | 19°02'38"E |
| September | Fen | cloudy | Fen2 | >60 | 23 | 20-60 | 05.09.2023 | 68°21'17"N | 19°02'38"E |
| September | Fen | cloudy | Fen2 | >60 | 17 | <10 | 05.09.2023 | 68°21'17"N | 19°02'38"E |
| September | Fen | cloudy | Fen3 | >60 | 15 | >70 | 08.09.2023 | 68°21'17"N | 19°02'37"E |
| September | Fen | cloudy | Fen3 | >60 | 16 | 20-60 | 08.09.2023 | 68°21'17"N | 19°02'37"E |
| September | Fen | cloudy | Fen3 | >60 | 16 | <10 | 08.09.2023 | 68°21'17"N | 19°02'37"E |
| June/July | Palsa | sunny | Palsa2 | 31 | - | >70 | 01.07.2023 | 68°21'20"N | 19°02'36"E |
| June/July | Palsa | sunny | Palsa2 | 31 | - | 20-60 | 01.07.2023 | 68°21'20"N | 19°02'36"E |
| June/July | Palsa | sunny | Palsa2 | 31 | - | <10 | 01.07.2023 | 68°21'20"N | 19°02'36"E |
| June/July | Palsa | sunny | Palsa2 | 32 | - | >70 | 01.07.2023 | 68°21'20"N | 19°02'36"E |
| June/July | Palsa | sunny | Palsa2 | 33 | - | 20-60 | 01.07.2023 | 68°21'20"N | 19°02'36"E |
| June/July | Palsa | sunny | Palsa2 | 32 | - | <10 | 01.07.2023 | 68°21'20"N | 19°02'36"E |
| August | Palsa | cloudy | Palsa1 | 46 | - | >70 | 12.08.2023 | 68°21'20"N | 19°02'39"E |
| August | Palsa | cloudy | Palsa1 | 46 | - | 20-60 | 12.08.2023 | 68°21'20"N | 19°02'39"E |
| August | Palsa | cloudy | Palsa1 | 46 | - | <10 | 12.08.2023 | 68°21'20"N | 19°02'39"E |
| August | Palsa | sunny | Palsa2 | 48 | - | >70 | 13.08.2023 | 68°21'20"N | 19°02'36"E |
| August | Palsa | sunny | Palsa2 | 48 | - | 20-60 | 13.08.2023 | 68°21'20"N | 19°02'36"E |
| August | Palsa | sunny | Palsa2 | 48 | - | <10 | 13.08.2023 | 68°21'20"N | 19°02'36"E |
| September | Palsa | sunny-cloudy | Palsa1 | 46 | - | >70 | 07.09.2023 | 68°21'20"N | 19°02'39"E |
| September | Palsa | sunny-cloudy | Palsa1 | 46 | - | 20-60 | 07.09.2023 | 68°21'20"N | 19°02'39"E |
| September | Palsa | sunny-cloudy | Palsa1 | 46 | - | <10 | 07.09.2023 | 68°21'20"N | 19°02'39"E |
| September | Palsa | cloudy | Palsa2 | 50 | - | >70 | 07.09.2023 | 68°21'20"N | 19°02'36"E |
| September | Palsa | cloudy | Palsa2 | 50 | - | 20-60 | 07.09.2023 | 68°21'20"N | 19°02'36"E |
| September | Palsa | cloudy | Palsa2 | 50 | - | <10 | 07.09.2023 | 68°21'20"N | 19°02'36"E |

**Tab. S2 | Overview of pH and soil temperature of palsa, bog and fen locations affected by vegetation density and growing season.** Soil pH and soil temperature averaged across biological replicates and depths**.** Mean ± 1SD. Porewater data of palsa in June missing due to dry soil conditions. *Measured with integrated temperature sensor of the redox probe at 25 cm depth.

| **Season** | **Stage** | **Vegetation density [%]** | **pH** | **SD** | **Soil temperature**  **[°C]** | **SD**  **[°C]** |
| --- | --- | --- | --- | --- | --- | --- |
| June/July | Palsa | <10 | x |  | 7.7* |  |
| June/July | Palsa | 20-60 | x |  | 9.8* | 0.71* |
| June/July | Palsa | >70 | x |  | 7.6* | 0.99* |
| June/July | Bog | <10 | 4.33 | 0.50 | 6.46 | 0.89 |
| June/July | Bog | 20-60 | 4.48 | 0.60 | 6.43 | 0.39 |
| June/July | Bog | >70 | 4.40 | 0.49 | 6.49 | 0.79 |
| June/July | Fen | <10 | 6.05 | 0.21 | 10.93 | 0.34 |
| June/July | Fen | 20-60 | 5.98 | 0.49 | 10.65 | 0.25 |
| June/July | Fen | >70 | 6.01 | 0.11 | 10.59 | 0.45 |
| August | Palsa | <10 | 5.41 | 0.45 | 7.34 | 0.93 |
| August | Palsa | 20-60 | 4.54 | 0.00 | 10.05 | 3.89 |
| August | Palsa | >70 | 4.62 | 0.31 | 7.20 | 0.29 |
| August | Bog | <10 | 4.67 | 0.56 | 9.02 | 1.62 |
| August | Bog | 20-60 | 4.83 | 0.31 | 10.73 | 0.55 |
| August | Bog | >70 | 4.87 | 0.23 | 11.00 | 1.22 |
| August | Fen | <10 | 6.02 | 0.18 | 11.03 | 0.50 |
| August | Fen | 20-60 | 6.04 | 0.17 | 11.12 | 0.08 |
| August | Fen | >70 | 6.00 | 0.27 | 11.65 | 0.78 |
| September | Palsa | <10 | 4.91 | 0.13 | 8.20 | 0.14 |
| September | Palsa | 20-60 | 4.63 | 0.29 | 8.00 | 0.71 |
| September | Palsa | >70 | 4.35 | 0.33 | 8.60 | 0.00 |
| September | Bog | <10 | 4.85 | 0.37 | 6.67 | 1.47 |
| September | Bog | 20-60 | 4.91 | 0.40 | 8.23 | 0.81 |
| September | Bog | >70 | 4.71 | 0.36 | 8.00 | 0.51 |
| September | Fen | <10 | 5.99 | 0.12 | 9.22 | 0.16 |
| September | Fen | 20-60 | 5.95 | 0.13 | 9.08 | 0.07 |
| September | Fen | >70 | 5.96 | 0.19 | 9.27 | 0.11 |

**Tab. S3 | Net ecosystem exchange (NEE) and ecosystem respiration (Reco) across thaw stages, vegetation conditions, and growing seasons.** Vegetation conditions include baseline (non-vascular plant reference), sparsely vegetated (“sparse”), and densely vegetated (“dense”) locations. Baseline plots were dominated by non-vascular vegetation (mosses/lichens) or waterlogged soil. R² indicates the goodness-of-fit of the linear regression applied to the concentration change during chamber incubation.

| **Location** | **Veg. density** | **R^2^** | **Season**  **month** | **NEE [mg/(minxm^2^)]** | **Location** | **Veg. density** | **R^2^** | **Season**  **month** | **NEE [mg/(minxm^2^)]** |
| --- | --- | --- | --- | --- | --- | --- | --- | --- | --- |
| Bog1 | sparse | >0.8 | August | -2.603 | Bog1 | sparse | >0.8 | June/July | -1.991 |
| Bog1 | sparse | >0.8 | August | -3.631 | Bog1 | sparse | >0.8 | June/July | -1.166 |
| Bog1 | sparse | >0.8 | August | -4.243 | Bog2 | baseline | >0.8 | June/July | -0.607 |
| Bog1 | baseline | >0.8 | August | -1.307 | Bog2 | baseline | >0.8 | June/July | 0.024 |
| Bog1 | baseline | >0.8 | August | -2.205 | Bog2 | baseline | >0.8 | June/July | -0.243 |
| Bog1 | baseline | >0.8 | August | -2.559 | Bog2 | dense | >0.8 | June/July | -1.870 |
| Bog1 | dense | >0.8 | August | -2.677 | Bog2 | dense | >0.8 | June/July | -2.186 |
| Bog1 | dense | >0.8 | August | -3.154 | Bog2 | dense | >0.8 | June/July | -2.016 |
| Bog1 | dense | >0.8 | August | -3.705 | Bog2 | sparse | >0.8 | June/July | -2.283 |
| Bog2 | baseline | >0.8 | August | -0.564 | Bog2 | sparse | >0.8 | June/July | -1.846 |
| Bog2 | baseline | >0.8 | August | -0.856 | Bog2 | sparse | >0.8 | June/July | -1.336 |
| Bog2 | baseline | >0.8 | August | -0.786 | Bog3 | baseline | >0.8 | June/July | -0.826 |
| Bog2 | sparse | >0.8 | August | -5.892 | Bog3 | baseline | >0.8 | June/July | 0.680 |
| Bog2 | sparse | >0.8 | August | -6.188 | Bog3 | baseline | >0.8 | June/July | -0.389 |
| Bog2 | sparse | >0.8 | August | -5.820 | Bog3 | dense | <0.8 | June/July | 1.117 |
| Bog2 | dense | >0.8 | August | -4.136 | Bog3 | dense | >0.8 | June/July | -0.049 |
| Bog2 | dense | >0.8 | August | -5.033 | Bog3 | dense | >0.8 | June/July | 1.457 |
| Bog2 | dense | >0.8 | August | -3.613 | Bog3 | sparse | <0.8 | June/July | -1.554 |
| Fen1 | baseline | >0.8 | August | 1.742 | Bog3 | sparse | >0.8 | June/July | -1.967 |
| Fen1 | baseline | >0.8 | August | 1.847 | Bog3 | sparse | >0.8 | June/July | -2.598 |
| Fen1 | baseline | >0.8 | August | 2.030 | Fen1 | baseline | >0.8 | June/July | 0.300 |
| Fen1 | sparse | >0.8 | August | -0.246 | Fen1 | baseline | >0.8 | June/July | 0.503 |
| Fen1 | sparse | >0.8 | August | 0.461 | Fen1 | baseline | >0.8 | June/July | 1.900 |
| Fen1 | sparse | >0.8 | August | -0.664 | Fen1 | dense | >0.8 | June/July | -4.584 |
| Fen2 | baseline | >0.8 | August | 2.607 | Fen1 | dense | >0.8 | June/July | -2.890 |
| Fen2 | baseline | >0.8 | August | 2.429 | Fen1 | sparse | <0.8 | June/July | -0.986 |
| Fen2 | baseline | >0.8 | August | 2.863 | Fen1 | sparse | >0.8 | June/July | -5.224 |
| Fen2 | dense | >0.8 | August | -3.448 | Fen1 | sparse | >0.8 | June/July | -1.891 |
| Fen2 | dense | >0.8 | August | -5.190 | Fen1 | sparse | >0.8 | June/July | -2.645 |
| Fen2 | sparse | >0.8 | August | -3.458 | Fen1 | sparse | >0.8 | June/July | -1.232 |
| Fen2 | sparse | >0.8 | August | -3.985 | Fen2 | baseline | >0.8 | June/July | 0.726 |
| Fen2 | sparse | >0.8 | August | -4.648 | Fen2 | baseline | >0.8 | June/July | 2.564 |
| Fen3 | baseline | >0.8 | August | 2.540 | Fen2 | dense | >0.8 | June/July | -4.105 |
| Fen3 | baseline | >0.8 | August | 3.132 | Fen2 | dense | >0.8 | June/July | -3.730 |
| Fen3 | dense | >0.8 | August | -0.704 | Fen2 | sparse | >0.8 | June/July | -0.308 |
| Fen3 | dense | >0.8 | August | -2.453 | Fen2 | sparse | >0.8 | June/July | -0.544 |
| Palsa1 | baseline | >0.8 | August | 0.281 | Fen2 | sparse | >0.8 | June/July | -1.612 |
| Palsa1 | baseline | >0.8 | August | 0.769 | Palsa1 | baseline | >0.8 | June/July | 0.569 |
| Palsa1 | baseline | >0.8 | August | 1.289 | Palsa1 | baseline | >0.8 | June/July | 1.541 |
| Palsa1 | dense | >0.8 | August | -1.770 | Palsa1 | dense | >0.8 | June/July | -0.510 |
| Palsa1 | dense | >0.8 | August | -0.741 | Palsa1 | dense | >0.8 | June/July | -0.782 |
| Palsa1 | dense | >0.8 | August | -0.258 | Palsa1 | sparse | >0.8 | June/July | 0.191 |
| Palsa1 | sparse | >0.8 | August | -0.930 | Palsa1 | sparse | >0.8 | June/July | -1.003 |
| Palsa1 | sparse | >0.8 | August | 1.873 | Palsa1 | sparse | >0.8 | June/July | 0.103 |
| Palsa1 | sparse | >0.8 | August | 0.517 | Palsa1 | sparse | >0.8 | June/July | -6.004 |
| Palsa2 | baseline | >0.8 | August | 2.147 | Palsa2 | baseline | >0.8 | June/July | 1.646 |
| Palsa2 | baseline | >0.8 | August | -0.456 | Palsa2 | baseline | >0.8 | June/July | 0.364 |
| Palsa2 | baseline | >0.8 | August | -0.394 | Palsa2 | dense | >0.8 | June/July | -0.170 |
| Palsa2 | dense | >0.8 | August | -2.550 | Palsa2 | dense | <0.8 | June/July | -0.207 |
| Palsa2 | dense | >0.8 | August | -1.500 | Palsa2 | sparse | >0.8 | June/July | -0.186 |
| Palsa2 | dense | >0.8 | August | -1.152 | Palsa2 | sparse | >0.8 | June/July | 0.031 |
| Palsa2 | sparse | >0.8 | August | 0.088 | Palsa2 | sparse | >0.8 | June/July | 0.373 |
| Palsa2 | sparse | <0.8 | August | -0.622 | Palsa2 | sparse | >0.8 | June/July | -0.494 |
| Palsa2 | sparse | >0.8 | August | -0.108 | Bog1 | baseline | <0.8 | September | -9.336 |
| Bog1 | baseline | >0.8 | June/July | -1.117 | Bog1 | baseline | >0.8 | September | -0.093 |
| Bog1 | baseline | >0.8 | June/July | -1.044 | Bog1 | dense | >0.8 | September | 5.549 |
| Bog1 | baseline | >0.8 | June/July | -0.291 | Bog1 | dense | <0.8 | September | 1.099 |
| Bog1 | dense | >0.8 | June/July | -3.764 | Bog1 | dense | >0.8 | September | -4.697 |
| Bog1 | dense | >0.8 | June/July | -3.400 | Bog1 | sparse | >0.8 | September | -2.813 |
| Bog1 | dense | >0.8 | June/July | -3.011 | Bog1 | sparse | >0.8 | September | -0.629 |
| Bog1 | sparse | >0.8 | June/July | -1.409 | Bog1 | sparse | >0.8 | September | -2.965 |

| **Location** | **Veg. density** | **R^2^** | **Season**  **month** | **NEE [mg/(minxm2)]** | **Location** | **Veg. density** | **R^2^** | **Season**  **month** | **Reco [mg/(minxm2)]** |
| --- | --- | --- | --- | --- | --- | --- | --- | --- | --- |
| Bog2 | baseline | >0.8 | September | 0.004 | Bog1 | baseline | >0.8 | August | 1.108 |
| Bog2 | baseline | >0.8 | September | -0.974 | Bog1 | baseline | >0.8 | August | 1.107 |
| Bog2 | baseline | >0.8 | September | -0.262 | Bog1 | dense | >0.8 | August | 5.953 |
| Bog2 | dense | >0.8 | September | 0.081 | Bog1 | dense | >0.8 | August | 4.198 |
| Bog2 | dense | >0.8 | September | -1.419 | Bog2 | baseline | >0.8 | August | 0.985 |
| Bog2 | dense | >0.8 | September | 1.409 | Bog2 | baseline | >0.8 | August | 0.486 |
| Bog2 | sparse | >0.8 | September | -2.543 | Bog2 | dense | >0.8 | August | 2.172 |
| Bog2 | sparse | >0.8 | September | -2.289 | Bog2 | dense | >0.8 | August | 1.915 |
| Bog2 | sparse | <0.8 | September | -0.014 | Bog3 | baseline | >0.8 | August | 1.872 |
| Bog3 | baseline | >0.8 | September | 0.926 | Bog3 | dense | >0.8 | August | 4.483 |
| Bog3 | baseline | <0.8 | September | 1.492 | Bog3 | dense | >0.8 | August | 5.805 |
| Bog3 | baseline | >0.8 | September | -3.099 | Fen1 | baseline | >0.8 | August | 9.339 |
| Bog3 | dense | >0.8 | September | -1.230 | Fen1 | baseline | >0.8 | August | 4.359 |
| Bog3 | dense | <0.8 | September | 2.141 | Fen1 | dense | >0.8 | August | 5.803 |
| Bog3 | dense | >0.8 | September | 3.845 | Fen1 | dense | <0.8 | August | 3.954 |
| Bog3 | sparse | >0.8 | September | -1.608 | Fen1 | dense | >0.8 | August | 10.229 |
| Bog3 | sparse | >0.8 | September | 6.170 | Fen2 | baseline | >0.8 | August | 4.063 |
| Bog3 | sparse | >0.8 | September | 5.538 | Fen2 | baseline | >0.8 | August | 3.074 |
| Fen1 | baseline | >0.8 | September | 3.213 | Fen2 | baseline | >0.8 | August | 3.454 |
| Fen1 | baseline | >0.8 | September | 2.994 | Fen2 | dense | >0.8 | August | 12.745 |
| Fen1 | baseline | >0.8 | September | 2.212 | Fen2 | dense | >0.8 | August | 8.812 |
| Fen1 | dense | >0.8 | September | 0.085 | Palsa1 | baseline | >0.8 | August | 1.568 |
| Fen1 | dense | >0.8 | September | 0.624 | Palsa1 | dense | >0.8 | August | 1.929 |
| Fen1 | dense | >0.8 | September | -0.653 | Palsa2 | dense | >0.8 | August | 3.085 |
| Fen1 | sparse | >0.8 | September | -0.877 | Palsa2 | baseline | >0.8 | August | 1.207 |
| Fen1 | sparse | >0.8 | September | -1.408 | Palsa2 | baseline | >0.8 | August | 1.984 |
| Fen1 | sparse | >0.8 | September | -1.964 | Fen1 | baseline | >0.8 | June/July | 1.138 |
| Fen2 | baseline | >0.8 | September | -8.340 | Fen1 | baseline | >0.8 | June/July | 3.385 |
| Fen2 | baseline | >0.8 | September | 4.244 | Fen1 | dense | >0.8 | June/July | 9.484 |
| Fen2 | baseline | <0.8 | September | -1.017 | Fen1 | dense | >0.8 | June/July | 8.165 |
| Fen2 | dense | <0.8 | September | 2.746 | Fen1 | dense | >0.8 | June/July | 9.484 |
| Fen2 | dense | >0.8 | September | 0.152 | Fen2 | baseline | >0.8 | June/July | 2.832 |
| Fen2 | dense | >0.8 | September | -3.929 | Fen2 | dense | >0.8 | June/July | 7.145 |
| Fen2 | sparse | >0.8 | September | -4.892 | Fen2 | dense | >0.8 | June/July | 8.365 |
| Fen2 | sparse | >0.8 | September | -6.372 | Palsa1 | baseline | >0.8 | June/July | 1.824 |
| Fen2 | sparse | >0.8 | September | -1.441 | Palsa1 | dense | >0.8 | June/July | 3.730 |
| Fen3 | baseline | >0.8 | September | -1.254 | Palsa1 | baseline | >0.8 | June/July | 2.340 |
| Fen3 | baseline | >0.8 | September | -1.336 | Palsa1 | dense | <0.8 | June/July | 0.000 |
| Fen3 | dense | >0.8 | September | 0.110 | Palsa2 | dense | >0.8 | June/July | 1.605 |
| Fen3 | sparse | >0.8 | September | 1.663 | Palsa2 | baseline | >0.8 | June/July | 2.551 |
| Fen3 | sparse | >0.8 | September | 2.159 | Palsa2 | dense | >0.8 | June/July | 1.512 |
| Palsa1 | baseline | >0.8 | September | 1.041 | Bog1 | baseline | >0.8 | September | -0.118 |
| Palsa1 | baseline | >0.8 | September | 1.893 | Bog1 | baseline | >0.8 | September | 0.888 |
| Palsa1 | baseline | >0.8 | September | 1.164 | Bog1 | baseline | <0.8 | September | 0.062 |
| Palsa1 | dense | >0.8 | September | 0.301 | Bog2 | baseline | <0.8 | September | 0.035 |
| Palsa1 | dense | >0.8 | September | -0.473 | Bog2 | baseline | >0.8 | September | 0.515 |
| Palsa1 | dense | >0.8 | September | -0.126 | Fen1 | baseline | >0.8 | September | 1.492 |
| Palsa1 | sparse | >0.8 | September | 0.330 | Fen1 | baseline | >0.8 | September | 2.124 |
| Palsa1 | sparse | >0.8 | September | 0.014 | Fen1 | dense | >0.8 | September | 1.304 |
| Palsa1 | sparse | >0.8 | September | 8.063 | Fen2 | baseline | >0.8 | September | 2.027 |
| Palsa2 | baseline | >0.8 | September | -0.015 | Fen2 | dense | >0.8 | September | 4.671 |
| Palsa2 | baseline | >0.8 | September | 0.497 | Fen2 | dense | >0.8 | September | 6.393 |
| Palsa2 | baseline | >0.8 | September | -0.109 |  |  |  |  |  |
| Palsa2 | dense | >0.8 | September | -1.404 |  |  |  |  |  |
| Palsa2 | dense | >0.8 | September | 0.339 |  |  |  |  |  |
| Palsa2 | dense | >0.8 | September | -1.222 |  |  |  |  |  |
| Palsa2 | sparse | >0.8 | September | -0.194 |  |  |  |  |  |
| Palsa2 | sparse | >0.8 | September | 0.391 |  |  |  |  |  |
| Palsa2 | sparse | >0.8 | September | -0.221 |  |  |  |  |  |

**Tab. S4 | CH_4_ emissions as CO_2_ equivalents across thaw stages, vegetation conditions, and growing season month.** Vegetation conditions include baseline (non-vascular plant reference), sparsely vegetated (“sparse”), and densely vegetated (“dense”) locations. Baseline plots were dominated by non-vascular vegetation (mosses/lichens) or waterlogged soil. R² indicates the goodness-of-fit of the linear regression applied to the concentration change during chamber incubation.

| **Location** | **Veg. density** | **Season**  **month** | **R²** | **CH_4_ [mg/(minxm^2^)]** | **Location** | **Veg. density** | **R^2^** | **Season**  **month** | **CH_4_ [mg/(minxm^2^)]** |
| --- | --- | --- | --- | --- | --- | --- | --- | --- | --- |
| Fen1 | baseline | August | >0.8 | 0.99 | Bog1 | sparse | June/July | >0.8 | 3.05 |
| Fen1 | baseline | August | >0.8 | 1.25 | Bog1 | sparse | June/July | >0.8 | 2.42 |
| Fen1 | baseline | August | >0.8 | 1.26 | Bog1 | sparse | June/July | >0.8 | 2.57 |
| Fen2 | baseline | August | >0.8 | 1.88 | Bog2 | sparse | June/July | >0.8 | 2.90 |
| Fen2 | baseline | August | >0.8 | 2.03 | Bog2 | sparse | June/July | >0.8 | 1.13 |
| Fen2 | baseline | August | >0.8 | 2.58 | Bog2 | sparse | June/July | >0.8 | 1.41 |
| Fen3 | baseline | August | >0.8 | 2.10 | Bog3 | sparse | June/July | >0.8 | 6.74 |
| Fen3 | baseline | August | >0.8 | 1.82 | Bog3 | sparse | June/July | >0.8 | 7.49 |
| Bog1 | baseline | August | >0.8 | 2.08 | Bog3 | sparse | June/July | >0.8 | 6.43 |
| Bog1 | baseline | August | >0.8 | 2.12 | Fen1 | dense | June/July | >0.8 | 23.95 |
| Bog1 | baseline | August | >0.8 | 2.15 | Fen1 | dense | June/July | >0.8 | 12.19 |
| Bog2 | baseline | August | >0.8 | 3.04 | Fen1 | dense | June/July | >0.8 | 11.75 |
| Bog2 | baseline | August | >0.8 | 3.28 | Fen2 | dense | June/July | >0.8 | 2.50 |
| Bog2 | baseline | August | >0.8 | 2.33 | Fen2 | dense | June/July | >0.8 | 7.37 |
| Fen1 | sparse | August | >0.8 | 4.90 | Fen2 | dense | June/July | >0.8 | 12.39 |
| Fen1 | sparse | August | >0.8 | 9.67 | Fen2 | dense | June/July | >0.8 | 13.08 |
| Fen1 | sparse | August | >0.8 | 4.36 | Bog1 | dense | June/July | >0.8 | 2.65 |
| Fen2 | sparse | August | >0.8 | 12.27 | Bog1 | dense | June/July | >0.8 | 2.46 |
| Fen2 | sparse | August | >0.8 | 7.06 | Bog2 | dense | June/July | >0.8 | 5.07 |
| Fen2 | sparse | August | >0.8 | 30.18 | Bog2 | dense | June/July | >0.8 | 5.12 |
| Bog1 | sparse | August | >0.8 | 7.55 | Bog2 | dense | June/July | >0.8 | 5.16 |
| Bog1 | sparse | August | >0.8 | 7.76 | Bog3 | dense | June/July | >0.8 | 12.45 |
| Bog1 | sparse | August | >0.8 | 7.95 | Bog3 | dense | June/July | >0.8 | 9.80 |
| Bog2 | sparse | August | >0.8 | 10.32 | Bog3 | dense | June/July | >0.8 | 9.23 |
| Bog2 | sparse | August | >0.8 | 9.95 | Bog1 | baseline | September | >0.8 | 9.74 |
| Bog2 | sparse | August | >0.8 | 10.40 | Bog1 | baseline | September | >0.8 | 3.80 |
| Fen1 | baseline | June/July | >0.8 | 2.56 | Bog2 | baseline | September | >0.8 | 1.01 |
| Fen1 | baseline | June/July | >0.8 | 1.69 | Bog2 | baseline | September | >0.8 | 0.81 |
| Fen1 | baseline | June/July | >0.8 | 1.48 | Bog3 | baseline | September | >0.8 | 2.84 |
| Fen2 | baseline | June/July | >0.8 | 1.76 | Bog3 | baseline | September | >0.8 | 0.87 |
| Fen2 | baseline | June/July | >0.8 | 2.24 | Fen1 | baseline | September | >0.8 | 7.28 |
| Fen2 | baseline | June/July | >0.8 | 3.61 | Fen1 | baseline | September | >0.8 | 20.53 |
| Fen2 | baseline | June/July | >0.8 | 3.03 | Fen1 | baseline | September | >0.8 | 3.83 |
| Bog1 | baseline | June/July | >0.8 | 0.78 | Fen2 | baseline | September | >0.8 | 8.01 |
| Bog1 | baseline | June/July | >0.8 | 0.98 | Fen2 | baseline | September | >0.8 | 4.07 |
| Bog1 | baseline | June/July | >0.8 | 0.85 | Fen3 | baseline | September | >0.8 | 16.15 |
| Bog2 | baseline | June/July | >0.8 | 0.83 | Fen3 | baseline | September | >0.8 | 3.90 |
| Bog2 | baseline | June/July | >0.8 | 0.94 | Fen1 | sparse | September | >0.8 | 5.00 |
| Bog2 | baseline | June/July | >0.8 | 1.06 | Fen1 | sparse | September | >0.8 | 3.95 |
| Fen1 | dense | August | >0.8 | 17.08 | Fen1 | sparse | September | >0.8 | 4.37 |
| Fen1 | dense | August | >0.8 | 16.12 | Fen2 | sparse | September | >0.8 | 8.15 |
| Fen1 | dense | August | >0.8 | 18.15 | Fen2 | sparse | September | >0.8 | 10.79 |
| Fen2 | dense | August | >0.8 | 21.96 | Bog1 | sparse | September | >0.8 | 7.26 |
| Fen2 | dense | August | >0.8 | 23.94 | Bog1 | sparse | September | >0.8 | 2.84 |
| Fen3 | dense | August | >0.8 | 9.22 | Bog1 | sparse | September | >0.8 | 6.30 |
| Fen3 | dense | August | >0.8 | 7.98 | Bog2 | sparse | September | >0.8 | 6.28 |
| Bog3 | baseline | June/July | >0.8 | 1.42 | Bog2 | sparse | September | >0.8 | 6.41 |
| Bog3 | baseline | June/July | >0.8 | 1.21 | Bog2 | sparse | September | >0.8 | 6.74 |
| Bog3 | baseline | June/July | >0.8 | 1.11 | Bog3 | sparse | September | >0.8 | 8.02 |
| Bog1 | dense | August | >0.8 | 4.91 | Bog3 | sparse | September | >0.8 | 7.84 |
| Bog1 | dense | August | >0.8 | 4.57 | Bog3 | sparse | September | >0.8 | 7.63 |
| Bog1 | dense | August | >0.8 | 4.86 | Fen1 | dense | September | >0.8 | 9.22 |
| Bog2 | dense | August | >0.8 | 13.08 | Fen1 | dense | September | >0.8 | 8.43 |
| Bog2 | dense | August | >0.8 | 13.49 | Fen1 | dense | September | >0.8 | 8.54 |
| Bog2 | dense | August | >0.8 | 12.11 | Fen2 | dense | September | >0.8 | 38.14 |
| Fen1 | sparse | June/July | >0.8 | 7.91 | Fen2 | dense | September | >0.8 | 8.47 |
| Fen1 | sparse | June/July | >0.8 | 4.69 | Fen2 | dense | September | >0.8 | 4.91 |
| Fen1 | sparse | June/July | >0.8 | 4.89 | Bog1 | dense | September | >0.8 | 2.18 |
| Fen2 | sparse | June/July | >0.8 | 7.54 | Bog1 | dense | September | >0.8 | 5.82 |
| Fen2 | sparse | June/July | >0.8 | 0.82 | Bog2 | dense | September | >0.8 | 8.95 |
| Fen2 | sparse | June/July | >0.8 | 1.16 | Bog2 | dense | September | <0.8 | 9.60 |
| Fen2 | sparse | June/July | >0.8 | 5.12 | Bog2 | dense | September | <0.8 | 9.39 |

**Tab. S5 ǀ Metabolites targeted in liquid and headspace (HS) gas chromatography coupled to a mass Spectrometer (GC-MS).** Targeted metabolites chosen based on AminiTabrizi et al. (2020)^(AminiTabrizi et al., 2020)^, Williams et al. (2021)^8 (Williams et al., 2021)^.

| **Metabolite** | **Method** | **Metabolite** | **Method** |
| --- | --- | --- | --- |
| Glycine | Liquide | Saccharic acid | Liquide |
| L-glutamine | Liquide | Formate/formic acid | HS |
| Glutamate | Liquide | Inostisol | Liquide |
| D L-isoleucine/leucine | Liquide | Xylose | Liquide |
| Phenylalanine | Liquide | Glucose | Liquide |
| Proline | Liquide | Galacitol | Liquide |
| Threonine | Liquide | Sucrose | Liquide |
| Histidine | Liquide | Fucose | Liquide |
| Aspargine | Liquide | Fructose | Liquide |
| Valine | Liquide | D-mannose | Liquide |
| Serine | Liquide | 4-hydroxybenzoic acid | Liquide |
| Alanine | Liquide | Hydroquinone | Liquide |
| Tyrosine | Liquide | D-galacturonic acid | Liquide |
| Cysteine | Liquide | *p*-Amino benzoic acid | Liquide |
| Pyruvic acid | Liquide | Auxin | Liquide |
| Succinic acid/succinate | Liquide | Biotin | Liquide |
| D-malic acid/malate | Liquide | Inositol | Liquide |
| Fumaric acid/fumarate | Liquide | Pantothenate | Liquide |
| Salicylic acid | Liquide | Pyridoxine | Liquide |
| Propionic acid | HS | Oxalic/oxalate | Liquide |
| Valeric acid | Liquide | Tartaric acid | Liquide |
| Malonic acid | Liquide | Acetic acid/acetate | HS |
| Butyric acid | HS | Lactic acid/lactate | Liquide/HS |
| Citric acid/citrate | Liquide |  |  |

**Tab. S6 | A univariate General Linear Model (GLM) was used to assess CO₂ emissions considering the effects of thawing stage and vegetation presence, as well as their interaction.** (A) Tests of Between-Subjects Effects. (B) Pairwise comparisons of estimated marginal means. Significant differences (p<0.05) are shown in bold.

| **A** | **Tests of “Between-Subjects” Effects** | | | | | | | | | |  |
| --- | --- | --- | --- | --- | --- | --- | --- | --- | --- | --- | --- |
| **Dependent Variable: CO_2_** | | | | | | | | | | |  |
| Source | | | | Type III Sum of Squares | | df | Mean Square | F | | Sig. |  |
| Corrected Model | | | | 283.664a | | 5 | 56.733 | 21.256 | | **<0.001** |  |
| Intercept | | | | 382.155 | | 1 | 382.155 | 143.180 | | **<0.001** |  |
| Soil | | | | 129.780 | | 2 | 64.890 | 24.312 | | **<0.001** |  |
| Vegetation presence | | | | 65.687 | | 1 | 65.687 | 24.611 | | **<0.001** |  |
| Soil * Vegetation presence | | | | 40.301 | | 2 | 20.151 | 7.550 | | **0.002** |  |
| Error | | | | 101.424 | | 38 | 2.669 |  | |  |  |
| Total | | | | 901.921 | | 44 |  |  | |  |  |
| Corrected Total | | | | 385.088 | | 43 |  |  | |  |  |
| a. R Squared = 0.737 (Adjusted R Squared = 0.702) | | | | | | | | | | |  |
| **B** | **Pairwise Comparison** | | | | | | | | | |  |
| **Independent variable: Vegetation** | | | | | | | | | | |  |
| **(I) Vegetation** | | **(J) Vegetation** | Mean Difference (I-J) | | Std. Error | Sig. | 95% Confidence Interval for Difference^b^  Upper Bound | | | |  |
|  |  |  |  |  |  |  | Lower Bound | | Upper Bound | |  |
| Bare | | Vegetated | -2.505^*^ | | 0.505 | **<0.001** | -3.527 | | -1.483 | |  |
| Vegetated | | Bare | 2.505^*^ | | 0.505 | **<0.001** | 1.483 | | 3.527 | |  |
| **Independent variable: Thaw stage** | | | | | | | | | | |  |
| (I) Thaw stage | | | (J) Thaw stage | Mean Difference (I-J) | | Std. Error | Sig. | 95% Confidence Interval for Difference^b^ | | | |
|  |  |  |  |  |  |  |  | Lower Bound | | Upper Bound | |
| Bog | | | Fen | -3.904^*^ | | 0.638 | **<0.001** | -5.195 | | -2.613 | |
|  |  |  | Palsa | -0.471 | | 0.629 | 0.459 | -1.744 | | 0.803 | |
| Fen | | | Bog  Palsa | 3.904^*^  3.433^*^ | | 0.638  0.587 | **<0.001**  **<0.001** | 2.613 | | | |
|  |  |  |  |  |  |  |  | 2.245 | | 4.622 | |
| Palsa | | | Bog | 0.471 | | 0.629 | 0.459 | -0.803 | | 1.744 | |
|  |  |  | Fen | -3.433^*^ | | 0.587 | **<0.001** | -4.622 | | -2.245 | |

**Tab. S7 | A univariate General Linear Model (GLM) was used to assess CH₄ emissions considering the effects of thawing stage and vegetation presence, as well as their interaction.** (A) Tests of Between-Subjects Effects. (B) Pairwise comparisons of estimated marginal means. Significant differences (p<0.05) are shown in bold.

| **A** | **Pairwise Comparison** | | | | | | | | | |  |
| --- | --- | --- | --- | --- | --- | --- | --- | --- | --- | --- | --- |
| **Dependent Variable: CH_4_** | | | | | | | | | | |  |
| Source | | | | Type III Sum of Squares | | df | Mean Square | F | | Sig. |  |
| Corrected Model | | | | 11.871a | | 5 | 2.374 | 11.999 | | **<0.001** |  |
| Intercept | | | | 5.433 | | 1 | 5.433 | 27.458 | | **<0.001** |  |
| Soil | | | | 3.840 | | 2 | 1.920 | 9.703 | | **<0.001** |  |
| Vegetation presence | | | | 3.041 | | 1 | 3.041 | 15.369 | | **<0.001** |  |
| Soil * Vegetation presence | | | | 2.631 | | 2 | 1.316 | 6.649 | | **0.002** |  |
| Error | | | | 13.257 | | 67 | 0.198 |  | |  |  |
| Total | | | | 34.799 | | 73 |  |  | |  |  |
| Corrected Total | | | | 25.128 | | 72 |  |  | |  |  |
| a. R Squared = 0.472 (Adjusted R Squared = 0.433) | | | | | | | | | | |  |
| **B** | **Pairwise Comparison** | | | | | | | | | |  |
| **Independent variable: Vegetation** | | | | | | | | | | |  |
| (I) **Vegetation** | | (J) **Vegetation** | Mean Difference (I-J) | | Std. Error | Sig. | 95% Confidence Interval for Difference  Upper Bound | | | |  |
|  |  |  |  |  |  |  | Lower Bound | | Upper Bound | |  |
| Bare | | Vegetated | -0.441* | | 0.113 | **<0.001** | -0.666 | | -0.217 | |  |
| Vegetated | | Bare | 0.441* | | 0.113 | **<0.001** | 0.217 | | 0.666 | |  |
| **Independent variable: Thaw stage** | | | | | | | | | | |  |
| (I) Thaw stage | | | (J) Thaw stage | Mean Difference (I-J) | | Std. Error | Sig. | 95% Confidence Interval for Difference | | | |
|  |  |  |  |  |  |  |  | Lower Bound | | Upper Bound | |
| Bog | | | Fen | -0.340* | | 0.115 | **0.004** | -0.569 | | -0.111 | |
|  |  |  | Palsa | 0.275 | | 0.148 | 0.068 | -0.020 | | 0.570 | |
| Fen | | | Bog  Palsa | 0.340*  0.615* | | 0.115  0.148 | **0.004**  **<0.001** | 0.111 | | | |
|  |  |  |  |  |  |  |  | 0.320 | | 0.910 | |
| Palsa | | | Bog | -0.275 | | 0.148 | 0.068 | -0.570 | | 0.020 | |
|  |  |  | Fen | -0.615* | | 0.148 | **<0.001** | -0.910 | | -0.320 | |

**Tab. S8 | A univariate General Linear Model (GLM) was used to assess CH₄ emissions in bog soils considering the effects of vegetation presence and emission pathway (transport vs. “stimulation” of methanogenesis), as well as their interaction. (**A) Tests of Between-Subjects Effects. (B) Pairwise comparisons of estimated marginal means. Significant differences (p<0.05) are shown in bold.

| **A** | **Tests of Between-Subjects Effects** | | | | | | | | | |
| --- | --- | --- | --- | --- | --- | --- | --- | --- | --- | --- |
| **Dependent Variable: CH_4_** | | | | | | | | | | |
| Source | | | | Type III Sum of Squares | | df | Mean Square | F | | Sig. |
| Corrected Model | | | | 0.571a | | 3 | 0.190 | 1.545 | | 0.235 |
| Intercept | | | | 13.442 | | 1 | 13.442 | 109.081 | | **<0.001** |
| Vegetation presence | | | | 0.335 | | 1 | 0.335 | 2.715 | | 0.116 |
| Emission pathway | | | | 0.138 | | 1 | 0.138 | 1.118 | | 0.304 |
| Vegetation presence * Emission pathway | | | | 0.149 | | 1 | 0.149 | 1.212 | | 0.285 |
| Error | | | | 2.341 | | 19 | 0.123 |  | |  |
| Total | | | | 16.013 | | 23 |  |  | |  |
| Corrected Total | | | | 2.913 | | 22 |  |  | |  |
| a. R Squared = 0.196 (Adjusted R Squared = 0.069) | | | | | | | | | | |
| **B** | **Pairwise Comparison** | | | | | | | | | |
| **Independent variable: Vegetation** | | | | | | | | | | |
| (I) Vegetation | | (J) Vegetation | Mean Difference (I-J) | | Std. Error | Sig. | 95% Confidence Interval for Difference | | | |
|  |  |  |  |  |  |  | Lower Bound | | Upper Bound | |
| Bare | | Vegetated | -0.242 | | 0.147 | 0.116 | -0.549 | | 0.065 | |
| Vegetated | | Bare | 0.242 | | 0.147 | 0.116 | -0.065 | | 0.549 | |
| **Independent variable: Emission pathway** | | | | | | | | | | |
| (I) Emission pathway | | (J) Emission pathway | Mean Difference (I-J) | | Std. Error | Sig. | 95% Confidence Interval for Difference  Upper Bound | | | |
|  |  |  |  |  |  |  | Lower Bound | | Upper Bound | |
| Transport | | “Stimulation” | 0.155 | | 0.147 | 0.304 | -0.152 | | 0.463 | |
| “Stimulation” | | Transport | -0.155 | | 0.147 | 0.304 | -0.463 | | 0.152 | |

**Tab. S9 | A univariate General Linear Model (GLM) was used to assess CH₄ emissions in fen soils considering the effects of vegetation presence and emission pathway (transport vs. “stimulation” of methanogenesis), as well as their interaction.** (A) Tests of Between-Subjects Effects. (B) Pairwise comparisons of estimated marginal means. Significant differences (p<0.05) are shown in bold.

| **A** | **Tests of Between-Subjects Effects** | | | | | | | | | |
| --- | --- | --- | --- | --- | --- | --- | --- | --- | --- | --- |
| **Dependent Variable: CH_4_** | | | | | | | | | | |
| Source | | | | Type III Sum of Squares | | df | Mean Square | F | | Sig. |
| Corrected Model | | | | 22.917^a^ | | 3 | 7.639 | 54.028 | | **<0.001** |
| Intercept | | | | 34.595 | | 1 | 34.595 | 244.679 | | **<0.001** |
| Vegetation presence | | | | 21.429 | | 1 | 21.429 | 151.559 | | **<0.001** |
| Emission pathway | | | | 0.798 | | 1 | 0.798 | 5.641 | | **0.028** |
| Vegetation presence * Emission pathway | | | | 0.690 | | 1 | 0.690 | 4.883 | | **0.039** |
| Error | | | | 2.828 | | 20 | 0.141 |  | |  |
| Total | | | | 60.339 | | 24 |  |  | |  |
| Corrected Total | | | | 25.744 | | 23 |  |  | |  |
| a. R Squared = 0.890 (Adjusted R Squared = 0.874) | | | | | | | | | | |
| **B** | **Pairwise Comparison** | | | | | | | | | |
| **Independent variable: Vegetation** | | | | | | | | | | |
| (I) Vegetation | | (J) Vegetation | Mean Difference (I-J) | | Std. Error | Sig. | 95% Confidence Interval for Difference | | | |
|  |  |  |  |  |  |  | Lower Bound | | Upper Bound | |
| Bare | | Vegetated | -1.890^*^ | | 0.154 | **<0.001** | -2.210 | | -1.570 | |
| Vegetated | | Bare | 1.890^*^ | | 0.154 | **<0.001** | 1.570 | | 2.210 | |
| **Independent variable: Emission pathway** | | | | | | | | | | |
| (I) Emission pathway | | (J) Emission pathway | Mean Difference (I-J) | | Std. Error | Sig. | 95% Confidence Interval for Difference  Upper Bound | | | |
|  |  |  |  |  |  |  | Lower Bound | | Upper Bound | |
| Transport | | “Stimulation” | 0.365^*^ | | 0.154 | **0.028** | 0.044 | | 0.685 | |
| “Stimulation” | | Transport | -0.365^*^ | | 0.154 | **0.028** | -0.685 | | -0.044 | |

**Tab. S10 | T-test-based p-values for CO_2_ and CH_4_ fluxes in palsa, bog, and fen in baseline (non-vascular plant reference) and vegetated locations.** Baseline plots were dominated by non-vascular vegetation (mosses/lichens) or waterlogged soil. (A) Chamber CO_2_ and CH_4_ emissions. (B) “Two-chamber” CO_2_ and CH_4_ for bog. (C) “Dual chamber” CO_2_ and CH_4_ for fen. Veg = vegetated, “plant” = CH_4_ emitted via the plant, “soil” = CH_4_ transported through the soil. Means are compared using the unpaired t-test at a 95% confidence interval. Normal distribution was tested before using a Shapiro-Wilk test for normality. If the data significantly deviated from a normal distribution, a non-parametric test (Wilcoxon rank sum test) was used. Significant differences (p<0.05) are shown in bold.

| **A** | **Chamber CO_2_** | | | | | | |
| --- | --- | --- | --- | --- | --- | --- | --- |
|  | | Palsa bare | Bog base | Fen base | Palsa veg | Bog veg | Fen veg |
| Palsa base | |  | **0.027** | **0.020** | 0.381 |  |  |
| Bog base | |  |  | **0.003** |  | **0.007** |  |
| Fen base | |  |  |  |  |  | **0.003** |
|  | **Chamber CH_4_** | | | | | | |
|  | | Palsa base | Bog base | Fen base | Palsa veg | Bog veg | Fen veg |
| Palsa base | |  | **<0.001** | **<0.001** | 0.619 |  |  |
| Bog base | |  |  | 0.157 |  | **<0.001** |  |
| Fen base | |  |  |  |  |  | **<0.001** |
| **B** | **Bog two-chamber CO_2_ flux** | | | | | | |
|  | | Base “soil” | Base “bag” | Veg “soil” | Veg “plant” |  |  |
| Base “soil” | |  | 0.886 | 0.254 | 0.353 |  |  |
| Base ”bag” | |  |  | 0.191 | 0.525 |  |  |
| Veg “soil” | |  |  |  | **0.015** |  |  |
|  | **Bog two-chamber CH_4_ flux** | | | | | | |
|  | | Base “soil” | Base “bag” | Veg “soil” | Veg “plant” |  |  |
| Base “soil” | |  | 0.978 | 0.550 | 0.060 |  |  |
| Base ”bag” | |  |  | 0.727 | 0.161 |  |  |
| Veg “soil” | |  |  |  | 0.132 |  |  |
| **C** | **Fen two-chamber CO_2_ flux** | | | | | | |
|  | | Base “soil” | Base “bag” | Veg “Soil” | Veg “plant” |  |  |
| Base “soil” | |  | 0.442 | 0.096 | 0.335 |  |  |
| Base ”bag” | |  |  | 0.259 | 0.156 |  |  |
| Veg “soil” | |  |  |  | **0.041** |  |  |
|  | **Fen two-chamber CH_4_ flux** | | | | | | |
|  | | Base “soil” | Base “bag” | Veg “soil” | Veg “plant” |  |  |
| Base “soil” | |  | 0.542 | **<0.001** | **<0.001** |  |  |
| Base ”bag” | |  |  | **<0.001** | **<0.001** |  |  |
| Veg “soil” | |  |  |  | **0.052** |  |  |

**Tab. S11 | T-test-based p-values for seasonal CO_2_:CH_4_ fluxes in bog obtained from dark (CO_2_) and light chamber measurements in baseline (non-vascular plant reference) and vegetated locations.** Baseline plots were dominated by non-vascular vegetation (mosses/lichens) or waterlogged soil. Means are compared using the unpaired t-test at a 95% confidence interval. Normal distribution was tested before using a Shapiro-Wilk test for normality. If the data significantly deviated from a normal distribution, a non-parametric test (Wilcoxon rank sum test) was used. Significant differences (p<0.05) are shown in bold.

| **Bog CO_2_:CH_4_** | | | | | | |
| --- | --- | --- | --- | --- | --- | --- |
|  | June base | June veg | Aug base | Aug veg | Sept base | Sept veg |
| Aug base |  |  |  | 0.485 | 0.217 |  |
| Aug veg |  |  |  |  |  | 0.694 |
| Sept base |  |  |  |  |  | 0.385 |
| **Fen CO_2_:CH_4_** | | | | | | |
|  | June bare | June veg | Aug base | Aug veg | Sept base | Sept veg |
| June base |  | 0.794 | 0.304 |  | 0.122 |  |
| June veg |  |  |  | 0.190 |  | 0.392 |
| Aug base |  |  |  | **0.041** | **0.026** |  |
| Aug veg |  |  |  |  |  | 0.628 |
| Sept base |  |  |  |  |  | 0.400 |

**Tab. S12 | A univariate General Linear Model (GLM) was used to assess CO_2_ emissions considering the effects of seasonality, thaw stage, and vegetation presence, as well as their interaction.** (A) Tests of Between-Subjects Effects. (B) Pairwise comparisons of estimated marginal means. Significant differences (p<0.05) are shown in bold.

| **A** | **Tests of Between-Subjects Effects** | | | | | | | | | |  |
| --- | --- | --- | --- | --- | --- | --- | --- | --- | --- | --- | --- |
| **Dependent Variable: CO_2_** | | | | | | | | | | |  |
| Source | | | | Type III Sum of Squares | | df | Mean Square | F | | Sig. |  |
| Corrected Model | | | | 278.093a | | 17 | 16.358 | **2.791** | | **<0.001** |  |
| Intercept | | | | 14.107 | | 1 | 14.107 | 2.407 | | 0.124 |  |
| **Season** | | | | 35.183 | | 2 | 17.591 | 3.002 | | **0.055** |  |
| **Vegetation** | | | | 70.528 | | 1 | 70.528 | 12.035 | | **<0.001** |  |
| **Thaw stage** | | | | 39.950 | | 2 | 19.975 | 3.409 | | **0.037** |  |
| **Season * Vegetation** | | | | 68.950 | | 2 | 34.475 | 5.883 | | **0.004** |  |
| Season * Thaw stage | | | | 33.018 | | 4 | 8.254 | 1.409 | | 0.237 |  |
| Vegetation * Thaw stage | | | | 29.821 | | 2 | 14.910 | 2.544 | | 0.084 |  |
| Season * Vegetation * Thaw stage | | | | 38.625 | | 4 | 9.656 | 1.648 | | 0.169 |  |
| Error | | | | 539.135 | | 92 | 5.860 |  | |  |  |
| Total | | | | 826.757 | | 110 |  |  | |  |  |
| Corrected Total | | | | 817.228 | | 109 |  |  | |  |  |
| a. R Squared = 0.340 (Adjusted R Squared = 0.218) | | | | | | | | | | |  |
| **B** | **Pairwise Comparison** | | | | | | | | | |  |
| ***Independent variable: Thaw stage*** | | | | | | | | | | |  |
| (I) Thaw stage | | (J) Thaw stage | Mean Difference (I-J) | | Std. Error | Sig.^b^ | 95% Confidence Interval for Difference^b^ | | | |  |
|  |  |  |  |  |  |  | Lower Bound | | Upper Bound | |  |
| Bog | | Fen | -0.895 | | 0.564 | 0.116 | -2.015 | | 0.226 | |  |
|  |  | Palsa | -1.462^*^ | | 0.576 | **0.013** | -2.606 | | -0.318 | |  |
| Fen | | Bog | 0.895 | | 0.564 | 0.116 | -.226 | | 2.015 | |  |
|  |  | Palsa | -0.567 | | 0.623 | 0.365 | -1.804 | | 0.670 | |  |
| Palsa | | Bog | 1.462^*^ | | 0.576 | **0.013** | 0.318 | | 2.606 | |  |
|  |  | Fen | 0.567 | | 0.623 | 0.365 | -0.670 | | 1.804 | |  |
| ***Independent variable: Vegetation*** | | | | | | | | | | |  |
| (I) Vegetation | | | (J) Vegetation | Mean Difference (I-J) | | Std. Error | Sig.^b^ | 95% Confidence Interval for Difference^b^ | | | |
|  |  |  |  |  |  |  |  | Lower Bound | | Upper Bound | |
| Bare | | | Vegetated | 1.666^*^ | | 0.480 | **<0.001** | 0.712 | | 2.620 | |
| Vegetated | | | Bare | -1.666^*^ | | 0.480 | **<0.001** | -2.620 | | -0.712 | |
| ***Independent variable: Season*** | | | | | | | | | | | |
| (I) Season | | | (J) Season | Mean Difference (I-J) | | Std. Error | Sig.^b^ | 95% Confidence Interval for Difference^b^ | | | |
|  |  |  |  |  |  |  |  | Lower Bound | | Upper Bound | |
| August | | | June | -0.123 | | 0.618 | 0.843 | -1.351 | | 1.106 | |
|  |  |  | September | -1.237^*^ | | 0.569 | **0.032** | -2.367 | | -0.107 | |
| June | | | August | 0.123 | | 0.618 | 0.843 | -1.106 | | 1.351 | |
|  |  |  | September | -1.114 | | 0.576 | **0.056** | -2.258 | | 0.030 | |
| September | | August | 1.237^*^ | | 0.569 | **0.032** | 0.107 | | 2.367 | |  |
|  |  | June | 1.114 | | 0.576 | **0.056** | -0.030 | | 2.258 | |  |

**Tab. S13 | A univariate General Linear Model (GLM) was used to assess CH_4_ emissions considering the effects of seasonality, thaw stage, and vegetation presence, as well as their interaction.** (A) Tests of Between-Subjects Effects. (B) Pairwise comparisons of estimated marginal means. Significant differences (p < 0.05) are shown in bold.

| **A** | **Tests of Between-Subjects Effects** | | | | | | | | | |  |
| --- | --- | --- | --- | --- | --- | --- | --- | --- | --- | --- | --- |
| **Dependent Variable: CH_4_** | | | | | | | | | | |  |
| Source | | | | Type III Sum of Squares | | df | Mean Square | F | | Sig. |  |
| Corrected Model | | | | 7.121a | | 11 | 0.647 | 4.705 | | **<0.001** |  |
| Intercept | | | | 15.245 | | 1 | 15.245 | 110.814 | | **<0.001** |  |
| Season | | | | 0.291 | | 2 | 0.145 | 1.057 | | 0.352 |  |
| Vegetation | | | | 3.475 | | 1 | 3.475 | 25.260 | | **<0.001** |  |
| Soil | | | | 1.117 | | 1 | 1.117 | 8.122 | | **0.006** |  |
| Season * Vegetation | | | | 1.325 | | 2 | 0.662 | 4.815 | | **0.011** |  |
| Season * Thaw stage | | | | 0.002 | | 2 | 0.001 | 0.007 | | 0.993 |  |
| Vegetation * Thaw stage | | | | 0.079 | | 1 | 0.079 | 0.575 | | 0.451 |  |
| Season * Vegetation * Thaw stage | | | | 0.958 | | 2 | 0.479 | 3.481 | | **0.036** |  |
| Error | | | | 11.006 | | 80 | 0.138 |  | |  |  |
| Total | | | | 32.808 | | 92 |  |  | |  |  |
| Corrected Total | | | | 18.127 | | 91 |  |  | |  |  |
| a. R Squared = 0.393 (Adjusted R Squared = 0.309) | | | | | | | | | | |  |
| **B** | **Pairwise Comparison** | | | | | | | | | |  |
| **Independent variable: Soil** | | | | | | | | | | |  |
| (I) Soil | | (J) Soil | Mean Difference (I-J) | | Std. Error | Sig.^b^ | 95% Confidence Interval for Difference^b^ | | | |  |
|  |  |  |  |  |  |  | Lower Bound | | Upper Bound | |  |
| Bog | | Fen | -0.223^*^ | | 0.078 | **0.006** | -0.378 | | -0.067 | |  |
| Fen | | Bog | 0.223^*^ | | 0.078 | **0.006** | 0.067 | | 0.378 | |  |
| **Independent variable: Vegetation** | | | | | | | | | | |  |
| (I) Vegetation | | (J) Vegetation | Mean Difference (I-J) | | Std. Error | Sig.^b^ | 95% Confidence Interval for Difference | | | |  |
|  |  |  |  |  |  |  | Lower Bound | | Upper Bound | |  |
| Bare | | Vegetated | -0.392^*^ | | 0.078 | **<0.001** | -0.548 | | -0.237 | |  |
| Vegetated | | Bare | 0.392^*^ | | 0.078 | **<0.001** | 0.237 | | 0.548 | |  |
| **Independent variable: Season** | | | | | | | | | | |  |
| (I) Season | | | (J) Season | Mean Difference (I-J) | | Std. Error | Sig.^b^ | 95% Confidence Interval for Difference^b^ | | | |
|  |  |  |  |  |  |  |  | Lower Bound | | Upper Bound | |
| August | | | June | 0.127 | | 0.098 | 0.198 | -0.068 | | 0.321 | |
|  |  |  | September | 0.016 | | 0.097 | 0.872 | -0.177 | | 0.208 | |
| June | | | August  September | -0.127  -0.111 | | 0.098  0.092 | 0.198  0.233 | -0.321 | | | |
|  |  |  |  |  |  |  |  | -0.295 | | 0.073 | |
| September | | | August | -0.016 | | 0.097 | 0.872 | -0.208 | | 0.177 | |
|  |  |  | June | 0.111 | | 0.092 | 0.233 | -0.073 | | 0.295 | |

**Tab. S14 | T-test-based p-values for net CO_2_ equivalent fluxes (CO_2_ flux added to CO_2_ equivalents of CH_4_ fluxes) from bog and fen separately for each growing season month (June (J), August (A), September (S)) in baseline (non-vascular plant reference) (1) and vegetated (2) locations.** Baseline plots were dominated by non-vascular vegetation (mosses/lichens) or waterlogged soil. Palsa not included since CH_4_ was negligible. Means are compared using the unpaired t-test at a 95% confidence interval. Normal distribution was tested before using a Shapiro-Wilk test for normality. If the data significantly deviated from a normal distribution, a non-parametric test (Wilcoxon rank sum test) was used. Significant differences (p<0.05) are shown in bold.

| **CO_2_ equivalent fluxes** | | | | | | | | | | | | | | | |
| --- | --- | --- | --- | --- | --- | --- | --- | --- | --- | --- | --- | --- | --- | --- | --- |
|  | J palsa2 | J bog2 | J fen2 | A palsa1 | A palsa2 | A bog1 | A bog2 | A fen1 | A fen2 | S palsa1 | S palsa2 | S bog1 | S bog2 | S fen1 | S fen2 |
| J palsa1 | **0.015** |  |  | 0.444 |  |  |  |  |  | 0.550 |  |  |  |  |  |
| J palsa2 |  |  |  |  | **0.040** |  |  |  |  |  | 0.970 |  |  |  |  |
| J bog1 |  | **0.052** |  |  |  | 0.373 |  |  |  |  |  | 0.170 |  |  |  |
| J bog2 |  |  |  |  |  |  | 1.00 |  |  |  |  |  | 0.993 |  |  |
| J fen1 |  |  | 0.277 |  |  |  |  | 0.121 |  |  |  |  |  | 0.293 |  |
| J fen2 |  |  |  |  |  |  |  |  | 0.200 |  |  |  |  |  | **0.012** |
| A palsa1 |  |  |  |  | **0.005** |  |  |  |  | 0.793 |  |  |  |  |  |
| A palsa2 |  |  |  |  |  |  |  |  |  |  | 0.960 |  |  |  |  |
| A bog1 |  |  |  |  |  |  | 0.132 |  |  |  |  | 0.284 |  |  |  |
| A bog2 |  |  |  |  |  |  |  |  |  |  |  |  | 1.000 |  |  |
| A fen1 |  |  |  |  |  |  |  |  | **0.032** |  |  |  |  | 0.486 |  |
| A fen2 |  |  |  |  |  |  |  |  |  |  |  |  |  |  | **0.042** |
| S palsa1 |  |  |  |  |  |  |  |  |  |  | **0.023** |  |  |  |  |
| S bog1 |  |  |  |  |  |  |  |  |  |  |  |  | 0.599 |  |  |
| S fen1 |  |  |  |  |  |  |  |  |  |  |  |  |  |  | 0.628 |

**Tab. S15 | T-test-based p-values for redox potential with depths and among thawing stages.** Means are compared using the unpaired t-test at a 95% confidence interval. Normal distribution was tested before using a Shapiro-Wilk test for normality. If the data significantly deviated from a normal distribution, a non-parametric test (Wilcoxon rank sum test) was used. Numbers indicate depth: 1=10 cm, 2=20 cm, 3=30 cm, 4=40 cm below water table surface. Significant differences (p<0.05) are shown in bold.

| **Redox potentials thawing stages** | | | | | | | | | | | | |
| --- | --- | --- | --- | --- | --- | --- | --- | --- | --- | --- | --- | --- |
|  | Palsa1 | Palsa2 | Palsa3 | Palsa4 | Bog1 | Bog2 | Bog3 | Bog4 | Fen1 | Fen2 | Fen3 | Fen4 |
| Palsa1 |  | **<0.001** |  |  | 0.545 |  |  |  |  |  |  |  |
| Palsa2 |  |  | **0.047** |  |  | **<0.001** |  |  |  |  |  |  |
| Palsa3 |  |  |  | **0.044** |  |  | **<0.001** |  |  |  |  |  |
| Palsa4 |  |  |  |  |  |  |  | **<0.001** |  |  |  |  |
| Bog1 |  |  |  |  |  | **<0.001** |  |  | **<0.001** |  |  |  |
| Bog2 |  |  |  |  |  |  | **<0.001** |  |  | **<0.001** |  |  |
| Bog3 |  |  |  |  |  |  |  | 0.386 |  |  | **<0.001** |  |
| Bog4 |  |  |  |  |  |  |  |  |  |  |  | **<0.001** |
| Fen1 |  |  |  |  |  |  |  |  |  | 0.478 |  |  |
| Fen2 |  |  |  |  |  |  |  |  |  |  | **<0.001** |  |
| Fen3 |  |  |  |  |  |  |  |  |  |  |  | 0.099 |
| Fen4 |  |  |  |  |  |  |  |  |  |  |  |  |

**Tab. S16 | T-test-based p-values for redox potential with depths and within a growing season.** Means are compared using the unpaired t-test at a 95% confidence interval. Normal distribution was tested before using a Shapiro-Wilk test for normality. If the data significantly deviated from a normal distribution, a non-parametric test (Wilcoxon rank sum test) was used. Numbers indicate depth: 1=10 cm, 2=20 cm, 3=30 cm, 4=40 cm below water table surface. Significant differences (p<0.05) are shown in bold.

| **Redox potentials Season** | | | | | | | | | | | | |
| --- | --- | --- | --- | --- | --- | --- | --- | --- | --- | --- | --- | --- |
|  | June1 | June2 | June3 | June4 | Aug1 | Aug2 | Aug3 | Aug4 | Sept1 | Sept2 | Sept3 | Sept4 |
| June1 |  | **0.014** |  |  | **0.002** |  |  |  |  |  |  |  |
| June2 |  |  | **<0.001** |  |  | **<0.001** |  |  |  |  |  |  |
| June3 |  |  |  | 0.232 |  |  | **0.007** |  |  |  |  |  |
| June4 |  |  |  |  |  |  |  | **0.013** |  |  |  |  |
| Aug1 |  |  |  |  |  | **<0.001** |  |  | **0.005** |  |  |  |
| Aug2 |  |  |  |  |  |  | **<0.001** |  |  | **<0.001** |  |  |
| Aug3 |  |  |  |  |  |  |  | 0.306 |  |  | **<0.001** |  |
| Aug4 |  |  |  |  |  |  |  |  |  |  |  | **<0.001** |
| Sept1 |  |  |  |  |  |  |  |  |  | **<0.001** |  |  |
| Sept2 |  |  |  |  |  |  |  |  |  |  | **<0.001** |  |
| Sept3 |  |  |  |  |  |  |  |  |  |  |  | 0.935 |
| Sept4 |  |  |  |  |  |  |  |  |  |  |  |  |

**Tab. S17 | T-test-based p-values for seasonal root-released C from *A. polifolia* (shrub) growing in palsas and graminoids growing in fens.** Means are compared using the unpaired t-test at a 95% confidence interval. Normal distribution was tested before using a Shapiro-Wilk test for normality. If the data significantly deviated from a normal distribution, a non-parametric test (Wilcoxon rank sum test) was used. Significant differences (p<0.05) are shown in bold.

| **Root-released C** | | | | | | |
| --- | --- | --- | --- | --- | --- | --- |
|  | Shrub June | Shrub Aug | Shrub Sept | Gram June | Gram Aug | Gram Sept |
| Shrub June |  | 0.495 | **0.015** | **<0.001** |  |  |
| Shrub Aug |  |  | **0.041** |  | **0.004** |  |
| Shrub Sept |  |  |  |  |  | **0.001** |
| Graminoid June |  |  |  |  | 0.182 | **0.020** |
| Graminoid Aug |  |  |  |  |  | **0.004** |

**References**

AminiTabrizi, R., Wilson, R. M., Fudyma, J. D., Hodgkins, S. B., Heyman, H. M., Rich, V. I., Tfaily, M. M. (2020). Controls on soil organic matter degradation and subsequent greenhouse gas emissions across a permafrost thaw gradient in Northern Sweden. *Frontiers in Earth Science, 8*, 557961.

Fiehn, O. (2016). Metabolomics by gas chromatography–mass spectrometry: Combined targeted and untargeted profiling. *Current protocols in molecular biology, 114*(1), 30.34. 31-30.34. 32.

Fiorini, D., Boarelli, M. C., Gabbianelli, R., Ballini, R., & Pacetti, D. (2016). A quantitative headspace–solid-phase microextraction–gas chromatography–flame ionization detector method to analyze short chain free fatty acids in rat feces. *Analytical biochemistry, 508*, 12-14.

ICOS Sweden, Lundin, E., Meire, A., Rakos, N. (2023). Ecosystem fluxes time series (ICOS Sweden) from Abisko-Stordalen Palsa Bog, 2019-12-31–2020-12-31, Swedish National Network, <https://hdl.handle.net/11676/hGV8GEHJL7sZNgr6OcQb-qYk>.

ICOS Sweden, Lundin, E., Rinne, J. (2022). Ecosystem fluxes time series (ICOS Sweden) from Abisko-Stordalen Palsa Bog, 2020-12-31–2021-12-31, Swedish National Network, <https://hdl.handle.net/11676/iRPe9DF30YbbyJQCOu3SBNwV>.

ICOS Sweden, Nilsson, M. (2021). Ecosystem fluxes time series (ICOS Sweden) from Abisko-Stordalen Palsa Bog, 2018-12-31–2019-12-31, Swedish National Network, <https://hdl.handle.net/11676/4n60Z_cdlL9PAd7C226rhpMe>.

ICOS Sweden, Rinne, J. (2019). Ecosystem fluxes time series (ICOS Sweden) from Abisko-Stordalen Palsa Bog, 2016-12-31–2017-12-31, Swedish National Network, <https://hdl.handle.net/11676/jGBBiZrsgz19J47noGGPzpPf>.

ICOS Sweden, Lundin, E., Rinne, J. (2021a). Ecosystem meteo time series (ICOS Sweden) from Abisko-Stordalen Palsa Bog, 2016-12-31–2017-12-31, Swedish National Network, <https://hdl.handle.net/11676/RsEekosuKKJQ_DfhhFdEQKOa>.

ICOS Sweden, Lundin, E., Rinne, J. (2021b). Ecosystem meteo time series (ICOS Sweden) from Abisko-Stordalen Palsa Bog, 2017-12-31–2018-12-31, Swedish National Network, <https://hdl.handle.net/11676/CLkw56hpo6p_Zy59eBnn5hY2>.

ICOS Sweden, Lundin, E., Rinne, J. (2021c). Ecosystem meteo time series (ICOS Sweden) from Abisko-Stordalen Palsa Bog, 2018-12-31–2019-12-31, Swedish National Network, <https://hdl.handle.net/11676/OxWpxiic7m5hvaErFHBzTS1q>.

ICOS Sweden, Lundin, E., Rinne, J. (2021d). Ecosystem meteo time series (ICOS Sweden) from Abisko-Stordalen Palsa Bog, 2019-12-31–2020-12-31, Swedish National Network, <https://hdl.handle.net/11676/pyzzIVYElaSZedqHz2DyjjWe>.

ICOS Sweden, Lundin, E., Rinne, J. (2022). Ecosystem meteo time series (ICOS Sweden) from Abisko-Stordalen Palsa Bog, 2020-12-31–2021-12-31, Swedish National Network, <https://hdl.handle.net/11676/xWk7ApA26gjb9o3hT0y98cCS>.

ICOS Sweden, Lundin, E., Rinne, J. (2023). Ecosystem meteo time series (ICOS Sweden) from Abisko-Stordalen Palsa Bog, 2021-12-31–2022-12-31, Swedish National Network, <https://hdl.handle.net/11676/qk6Tpkpn5vkR4n5RvJRZKF-G>.

Laasonen, A., Buzacott, A., Kohonen, K.-M., Lundin, E., Meire, A., Pihlatie, M., & Mammarella, I. (2025). Radiation and surface wetness drive carbon monoxide fluxes from an Arctic peatland. *EGUsphere, 2025*, 1-24.

Lundin, E., Crill, P., Grudd, H., Gustafsson, J., Holst, J., Kristoffersson, A., Meire, A., Molder, M., Rakos, N. (2025). ETC L2 Meteo from Abisko-Stordalen Palsa Bog, 2021-12-31–2025-09-30, ICOS RI, <https://hdl.handle.net/11676/FyDlCb9BGrVautCxouxhyENd>.

Lundin, E., Crill, P., Grudd, H., Holst, J., Kristoffersson, A., Meire, A., Mölder, M., Rakos, N. (2025). ETC L2 Fluxes from Abisko-Stordalen Palsa Bog, 2021-12-31–2024-12-31, ICOS RI, <https://hdl.handle.net/11676/8iCfE5-7bQy6AtQcJgs5U1nD>.

Williams, A., Langridge, H., Straathof, A. L., Muhamadali, H., Hollywood, K. A., Goodacre, R., & de Vries, F. T. (2021). Root functional traits explain root exudation rate and composition across a range of grassland species. *Journal of Ecology, 110*(1), 21-33. doi:10.1111/1365-2745.13630

Zhang, C., Tang, P., Xu, H., Weng, Y., Tang, Q., & Zhao, H. (2018). Analysis of short-chain fatty acids in fecal samples by headspace-gas chromatography. *Chromatographia, 81*, 1317-1323.
